# Incidence of Malaria and Efficacy of Combination Antimalarial Therapies over 4 Years in an Urban Cohort of Ugandan Children

**DOI:** 10.1371/journal.pone.0011759

**Published:** 2010-07-30

**Authors:** Tamara D. Clark, Denise Njama-Meya, Bridget Nzarubara, Catherine Maiteki-Sebuguzi, Bryan Greenhouse, Sarah G. Staedke, Moses R. Kamya, Grant Dorsey, Philip J. Rosenthal

**Affiliations:** 1 Department of Medicine, University of California San Francisco, San Francisco, California, United States of America; 2 Makerere University Medical School, Kampala, Uganda; 3 London School of Hygiene and Tropical Medicine, London, United Kingdom; Mahidol University, Thailand

## Abstract

**Background:**

Combination therapies are now recommended to treat uncomplicated malaria. We used a longitudinal design to assess the incidence of malaria and compare the efficacies of 3 combination regimens in Kampala, Uganda.

**Methodology/Principal Findings:**

Children aged 1–10 years were enrolled from randomly selected households in 2004–05 and 2007, and were followed at least monthly through 2008. Insecticide-treated bednets (ITNs) were provided in 2006. Children were randomized upon their first episode, and then treated for all episodes of uncomplicated malaria with amodiaquine/sulfadoxine-pyrimethamine (AQ/SP), artesunate/amodiaquine (AS/AQ), or artemether/lumefantrine (AL). Risks of parasitological failure were determined for each episode of uncomplicated malaria and clinical parameters were followed.

A total of 690 children experienced 1464 episodes of malaria. 96% of these episodes were uncomplicated malaria and treated with study drugs; 94% were due to *Plasmodium falciparum*. The rank order of treatment efficacy was AL > AS/AQ > AQ/SP. Failure rates increased over time for AQ/SP, but not the artemisinin-based regimens. Over the 4-year course of the study the prevalence of asymptomatic parasitemia decreased from 11.8% to 1.4%, the incidence of malaria decreased from 1.55 to 0.32 per person year, and the prevalence of anemia (hemoglobin <10 gm/dL) decreased from 5.9% to 1.0%. No episodes of severe malaria (based on WHO criteria) and no deaths were seen.

**Conclusions/Significance:**

With ready access to combination therapies and distribution of ITNs, responses were excellent for artemisinin-containing regimens, severe malaria was not seen, and the incidence of malaria and prevalence of parasitemia and anemia decreased steadily over time.

**Trial Registration:**

isrctn.org ISRCTN37517549

## Introduction

Malaria is one of the most important infectious diseases in the world. The problem is greatest in sub-Saharan Africa, where infection with *Plasmodium falciparum*, the most virulent human malaria parasite, is responsible for hundreds of millions of illnesses and about one million deaths each year [1]. Recent advances have led to some optimism regarding the control of malaria, and there have been documented decreases in malarial incidence in a number of parts of Africa [2]–[6]. However, the problem remains great, with little improvement in much of Africa, and improved malaria control interventions are needed. One important component of malaria control is the prompt administration of highly effective therapies to those ill with malaria. With increasing resistance to older therapies, current WHO recommendations call for the treatment of uncomplicated malaria in Africa with artemisinin-based combination therapy (ACT). Multiple studies have shown excellent efficacy for the two widely advocated ACTs for Africa, artesunate/amodiaquine (AS/AQ) and artemether/lumefantrine (AL) [7]–[12]. In addition, the non-ACT combination regimen amodiaquine/sulfadoxine-pyrimethamine has shown good antimalarial efficacy in many [13]–[16], but not all [7], [12] trials in Africa, and is recommended by the WHO as an alternative therapy for malaria when ACTs are unavailable.

The epidemiology of malaria varies greatly across Africa, with large variations in transmission intensity and both seasonal and year-round transmission in different areas. The greater the transmission intensity, the more rapidly humans acquire the partial immunity to malaria that allows older children and adults to be relatively spared from malarial incidence and risks of severe malaria. Thus, in high transmission areas children acquire effective immunity over the first few years of life, and malarial illness is uncommon in older children and adults. In areas with lower levels of transmission, including many cities, the acquisition of immunity occurs more slowly. The level of immunity in a population directly impacts upon responses to antimalarial therapies, especially in the context of sub-optimal treatments, as the immune response plays an important role in control of infection, and there is a clear correlation between transmission intensity, level of antimalarial immunity, and response to therapy with partially effective agents [17]–[18].

We studied the comparative efficacy of 3 antimalarial combination therapies in an urban cohort of children in Kampala, a city with a level of malaria transmission that is relatively low for sub-Saharan Africa. An interim analysis identified a clear rank-order of treatment efficacy (AL > AS/AQ > AQ/SP) [12]. Malarial incidence was heterogeneous, and associated with host genetics, use of bednets, and distance of a child's residence from a mosquito breeding site [19]. The efficacy of AQ/SP diminished over time, but this change appeared to be due to changes in host immunity rather than increasing parasite resistance to the components of AQ/SP [20]. We now present a final evaluation of the relative efficacies of the 3 combination antimalarial therapies, changes in the prevalence of malarial parasitemia and incidence of malaria over time, and other clinical features in this urban cohort of Ugandan children.

## Methods

The protocol for this trial and supporting CONSORT checklist are available as supporting information; see Checklist S1 and Protocol S1.

### Enrollment of cohort

A detailed description of the cohort has been published previously [12]. The study was conducted in the Mulago III parish of Kampala, where malaria is reportedly mesoendemic, occurring throughout the year, with peaks during and after the two rainy seasons. A census was carried out from July to October 2004, enumerating a population of 16,172 [21]. All children from randomly selected households were screened for enrollment. Enrollment took place between November 2004 and April 2005 and again from January–May 2007 to enroll younger children from study households who had reached an eligible age. Eligibility criteria were: age 1 to 10 years, agreement to come to the study clinic for any febrile episode or illness, agreement to avoid medications administered outside the study, agreement to remain in Kampala during the study period, no known adverse reactions to the study medications, weight 10 kg or more, absence of severe malnutrition or known serious chronic disease, absence of life threatening screening laboratory results, and willingness of parent or guardian to provide written informed consent. Children with symptomatic malaria on the day of screening were treated with quinine and enrolled only after documentation of a negative blood smear 7 days after initiation of therapy. The study received ethical approval from the Uganda National Council of Science and Technology, the Makerere University Research and Ethics Committee, and the University of California, San Francisco Committee on Human Research.

### Follow-up of study participants

Parents and guardians were asked to bring their children to the study clinic for all medical care. The clinic was open daily from 8 AM to 5 PM, and after-hours care was available. Participants who presented with new medical problems underwent a standardized evaluation. Malaria was diagnosed if a child had complicated malaria (presence of severe malaria or danger signs [22]) or fever (documented tympanic temperature 38.0°C or history of fever in the previous 24 hours) and any parasitemia. Children underwent routine clinical assessment at least monthly. Blood smears were performed monthly until April 2006, and every 3 months thereafter. Asymptomatic parasitemia was not treated. Medications with antimalarial activity were avoided for the treatment of nonmalarial illnesses if possible.

### Treatment allocation and study drug administration

Participants were randomly assigned to receive 1 of 3 oral regimens at their first episode of uncomplicated malaria and then for all subsequent episodes diagnosed during the study. Study medications were administered by the nurses according to modified WHO recommendations for weight-based administration of fractions of tablets. The regimens were: AQ/SP (AQ, 10 mg/kg on the first 2 days and 5 mg/kg on the third day; sulfadoxine, 25 mg/kg and pyrimethamine, 1.25 mg/kg on the first day); AS/AQ (AQ as above and artesunate, 4 mg/kg on all 3 days); and artemether 20 mg/lumefantrine 120 mg twice daily for 3 days (5 through 14 kg, 1 tablet; 15 through 24 kg, 2 tablets; and 25 through 34 kg, 3 tablets per dose). Administration of first daily doses were directly observed by study nurses; second daily doses of medication or placebo (for AQ/SP and AS/AQ arms) were given to the parents or guardians to administer in the evening. All study personnel involved in outcome assessment were blinded to treatment allocation. Study participants and their caregivers were not informed of their assigned treatment regimens. Placebos were administered to match the twice-daily schedule of AL. However, because the study drugs were not identical in appearance and taste, the study was considered single-blind. After treatment in the clinic, patients were observed for 30 minutes and the dose was readministered if vomiting occurred. Patients who vomited persistently were referred for treatment with parenteral quinine. Participants with severe malaria or danger signs were also treated with quinine. The AQ/SP treatment arm was discontinued in March 2007 after our interim analysis demonstrated inferior efficacy [12], and children in this arm were randomized to one of the other two arms. The AS/AQ arm was discontinued in January 2008 due to loss of availability of the study formulation of AS, and children in this arm were moved to the AL arm.

### Follow-up and outcome classification

Participants diagnosed with malaria were asked to return on days 1, 2, 3, 7, 14, and 28 or any other day they felt ill. Follow-up evaluation consisted of a standardized medical history and physical examination. Blood was obtained by finger prick for thick blood smears and storage on filter paper on all follow-up days, except day 1. Treatment outcomes were classified according to 2005 WHO guidelines; for this analysis all failures (early treatment failure, late clinical failure, and late parasitological failure) were combined. Patients with failure within 14 days of initiation of therapy were treated with quinine (10 mg/kg three times a day for 7 days, unsupervised), beginning a new 28-day follow-up schedule. Symptomatic malaria diagnosed more than 14 days after a previous episode was managed according to the study protocol. Safety outcomes have been reported previously [12], [23].

### Laboratory methods

Blood smears and molecular genotyping methods to distinguish recrudescences from new infections were performed as previously described [12]. When *P. falciparum* DNA was not identified by genotyping in a sample with a positive blood smear, parasite species was determined by amplification of 18S small subunit ribosomal DNA, as previously described [24].

### Statistical analysis

The sample size for this study was calculated and outcomes were analyzed as previously described [12]. Based on an interim analysis, the data and safety monitoring board recommended that the AQ/SP treatment arm be dropped in March, 2007. Efficacy data were evaluated with an intention-to-treat analysis including all patients with falciparum malaria who were randomized to study drug therapy [12]. Risks of treatment failure were estimated with the Kaplan-Meier product limit formula. Data were censored for patients who did not complete follow-up or were reinfected with non-falciparum species and for new infections according to outcomes adjusted by genotyping. Changes in hemoglobin over time were analyzed using generalized estimating equations to account for repeated measures within individuals.

## Results

### Characteristics of study participants

**Figure 1:**
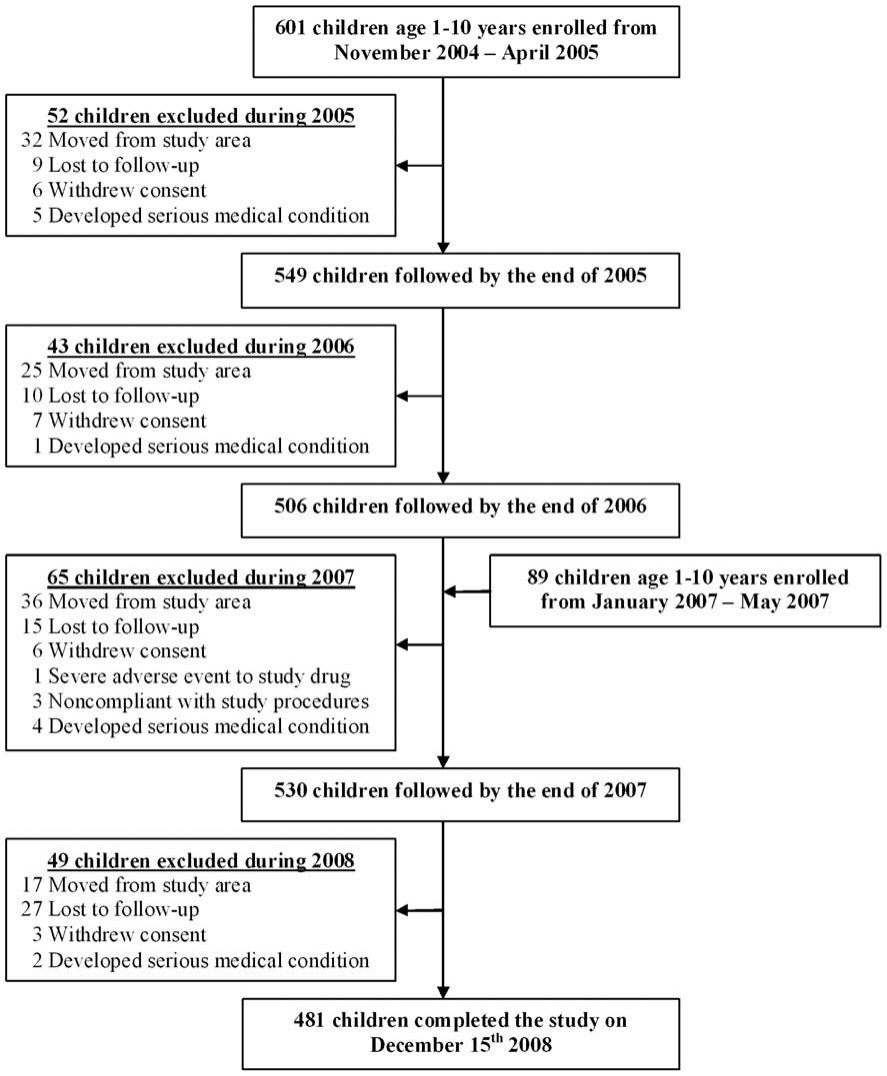
Trial profile.

The cohort included 690 children aged 1 to 10 years at enrollment; 601 were enrolled from November 2004-April 2005, and an additional 89 from January–May 2007 (figure 1). All participants were from randomly selected households in the Mulago III parish of Kampala [21]. The second period of enrollment included only children from households already taking part in the study, to include younger children who had reached an eligible age since the initial period of enrollment. Characteristics of study participants are shown in table 1. Of these, 209 children were withdrawn before the end of study follow-up in December 2008 for the following reasons: movement out of study area (n = 110), inability to locate participant after 60 consecutive days (n = 61), withdrawal of informed consent (n = 22), inability to comply with the study procedures (n = 3), serious adverse event due to study medication (n = 1), and diagnosis of serious chronic disease requiring frequent medical care (n = 12). The percentage of participants lost to follow-up was similar during each year of the study (7.8% to 9.5% per year), and it did not differ between treatment arms. A total of 481 (69.7%) children completed the study in December 2008. At enrollment bednet use was reported in 43% of the cohort, but only 6% reported using an insecticide-treated bednet (ITN) [21]. Between May and June 2006, ITNs were distributed to all study participants.

**Table 1:** Description of patient population and malaria incidence by year.

Variable	Year				
	2004	2005	2006	2007	2008
Number of children observed	240	601	549	595	530
Female, n (%)	130 (54%)	287 (48%)	259 (47%)	291 (49%)	257 (48%)
Person years of observation	23	520	523	543	478
Median age in years (IQR)	6.0 (4.1–8.1)	6.5 (4.1–8.5)	7.5 (5.1–9.5)	7.9 (5.3–10.3)	8.9 (6.2–11.2)
ITN coverage^1^	7.2%	6.2%	64.7%	99.4%	100%
Episodes of malaria	36	530	451	294	153
New episodes of malaria	36 (100%)	499 (94.2%)	420 (93.1%)	276 (93.9%)	150 (98.0%)
Recrudescent episodes of malaria	0	31 (5.8%)	31 (6.9%)	18 (6.1%)	3 (2.0%)
Parasite species^2^					
*P. falciparum*	35 (97.2%)	497 (93.8%)	431 (95.6%)	272 (92.5%)	142 (92.8%)
*P. malariae*	1 (2.8%)	25 (4.7%)	14 (3.1%)	18 (6.1%)	9 (5.9%)
*P. ovale*	0	6 (1.1%)	4 (0.9%)	2 (0.7%)	1 (0.7%)
*P. vivax*	0	2 (0.4%)	2 (0.4%)	2 (0.7%)	1 (0.7%)
Treatments for malaria					
AQ+SP^3^	13 (36.1%)	179 (33.8%)	164 (36.4%)	40 (13.6%)	0
AS+AQ^4^	11 (30.6%)	164 (30.9%)	136 (30.2%)	105 (35.7%)	18 (11.8%)
AL	12 (33.3%)	153 (28.9%)	140 (31.0%)	142 (48.3%)	132 (86.3%)
Quinine	0	28 (5.3%)	10 (2.2%)	5 (2.0%)	3 (2.0%)
Quinine + Clindamycin	0	6 (1.1%)	1 (0.2%)	2 (0.7%)	0
Asymptomatic parasitemia	44/374 (11.8%)	401/5351 (7.5%)	97/3228 (3.0%)	52/1935 (2.7%)	24/1681 (1.4%)
Mean hemoglobin gm/dL (SD)	12.1 (1.3)	12.0 (1.3)	12.3 (1.2)	12.3 (1.1)	12.7 (1.1)
Hemoglobin <10 gm/dL	18/306 (5.9%)	200/3002 (6.7%)	85/2687 (3.2%)	83/2666 (3.1%)	21/2057 (1.0%)
Incidence all episodes of malaria (95% CI)^5^	1.55 (1.09–2.14)	1.02 (0.94–1.11)	0.86 (0.79–0.95)	0.54 (0.48–0.61)	0.32 (0.27–0.38)
Incidence new episodes of malaria (95% CI)^5^	1.55 (1.09–2.14)	0.96 (0.88–1.05)	0.80 (0.73–0.88)	0.50 (0.45–0.57)	0.31 (0.27–0.37)

1 percentage of days of observation with ITN coverage.

2 considering only symptomatic infections.

3 discontinued in March, 2007.

4 discontinued in January, 2008.

5 per person years of observation.

### Species of infecting parasites

As expected most episodes of malaria (94%) were caused by *P. falciparum*. When *P. falciparum* was identified, we did not search for other species, so mixed infections were not identified. However, the three other human malaria parasite species were all seen, with disease caused solely by *P. malariae* in 66 episodes, *P. ovale* in 13 episodes, and *P. vivax* in 7 episodes.

### Asymptomatic parasitemia and incidence of malaria

Asymptomatic parasitemia was common at study enrollment in 2004–05 (17%) [21]. The prevalence of asymptomatic parasitemia decreased over the course of the study to only 1.4% in 2008 (table 1). A total of 1464 episodes of malaria, defined as documented fever or history of fever with any parasitemia on blood smear, occurred over the study period. Of the 690 children enrolled in the study, 430 had at least one episode of malaria (range 1–30 episodes), whereas 260 experienced no episodes.

**Figure 2:**
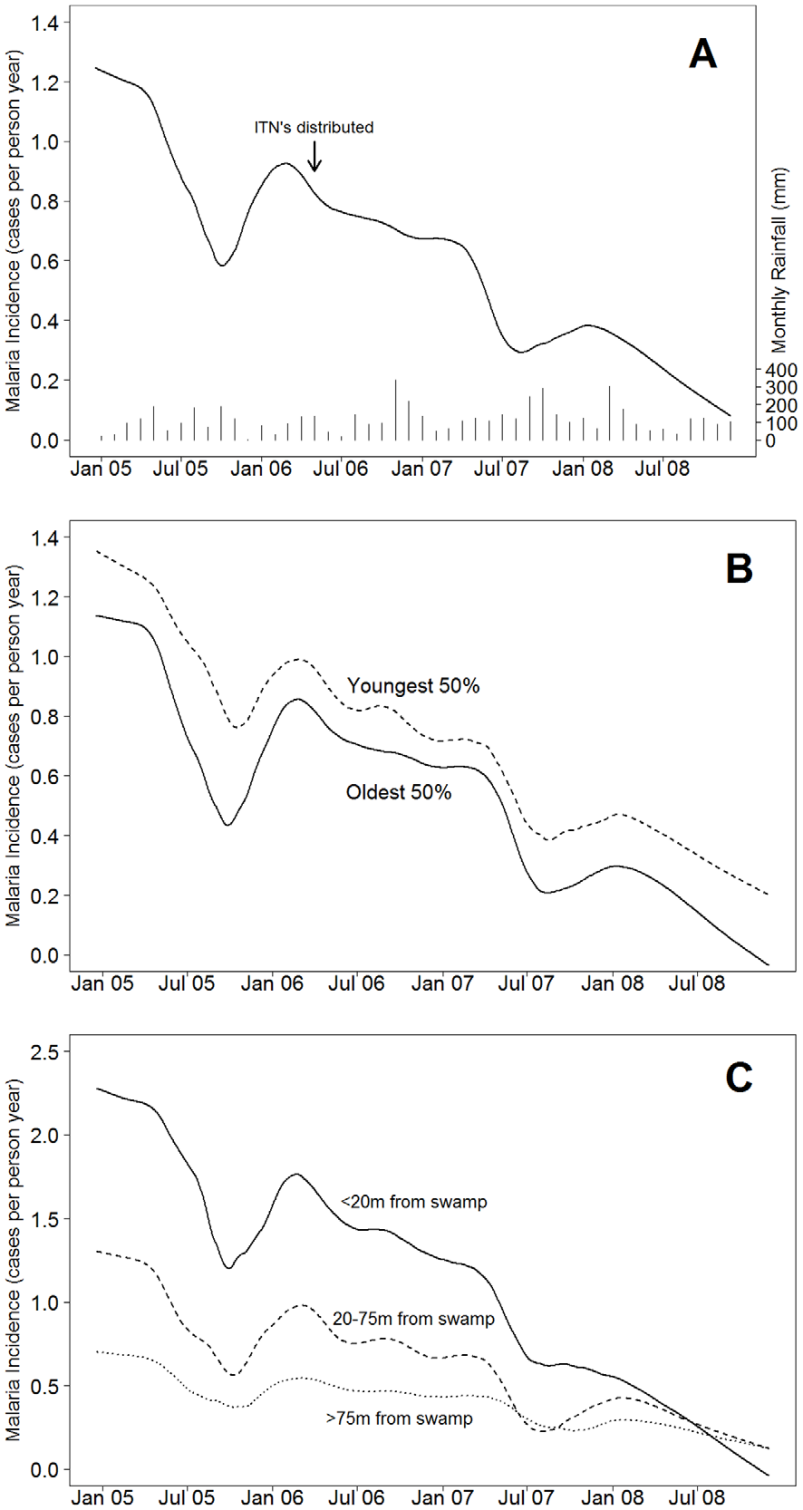
Incidence of malaria over time. Incidence of malaria in the full cohort (A), stratified for age (B), and stratified based on distance of residence from a swamp (C) are shown. Smoothed lines were produced in R (version 2.9.0) using Friedman's SuperSmoother and considering 1339 new episodes of malaria over 710,004 person-days of follow up. This analysis did not include the 89 children enrolled in 2007. Monthly rainfall data are from the Uganda Department of Meteorology; measurements were at Makerere University, <2 km from the Mulago III parish, where study subjects resided.

The incidence of malaria was year-round, with large variation, consistent with variable year-round rainfall and malaria transmission in Kampala (figure 2A). Incidence decreased steadily over the 4 full years of follow-up, from 1.02 episodes per person year in 2005 to 0.32 episodes per person year in 2008 (table 1). Malaria incidence decreased with increasing age, with the highest incidence in children aged 0 to 5 years (0.87 per person year) and lowest in those 11 to 15 years (0.48 per person year). However, age-related differences may be overstated in this analysis, since older ages contributed more person time late in the study, when incidence was lower. In any event, differences in incidence among these age groups were less than in reports from other areas of Africa with high transmission intensity [25], consistent with the moderate level of malaria transmission in Kampala. Changes in incidence over time were very similar for younger and older children in the cohort (figure 2B). Malaria risk was greater in children residing closer to a mosquito breeding site [19], but differences based on place of residence decreased over time, such that by the end of the study these differences were lost (figure 2C). ITN use was associated with a reduction in the incidence of malaria from 1.08 to 0.51 per person year. However, ITN use did not fully explain the decreasing incidence of malaria, as marked decreases were seen both before and after the widespread initiation of ITN use in May–June 2006.

### Treatment outcomes

**Figure 3:**
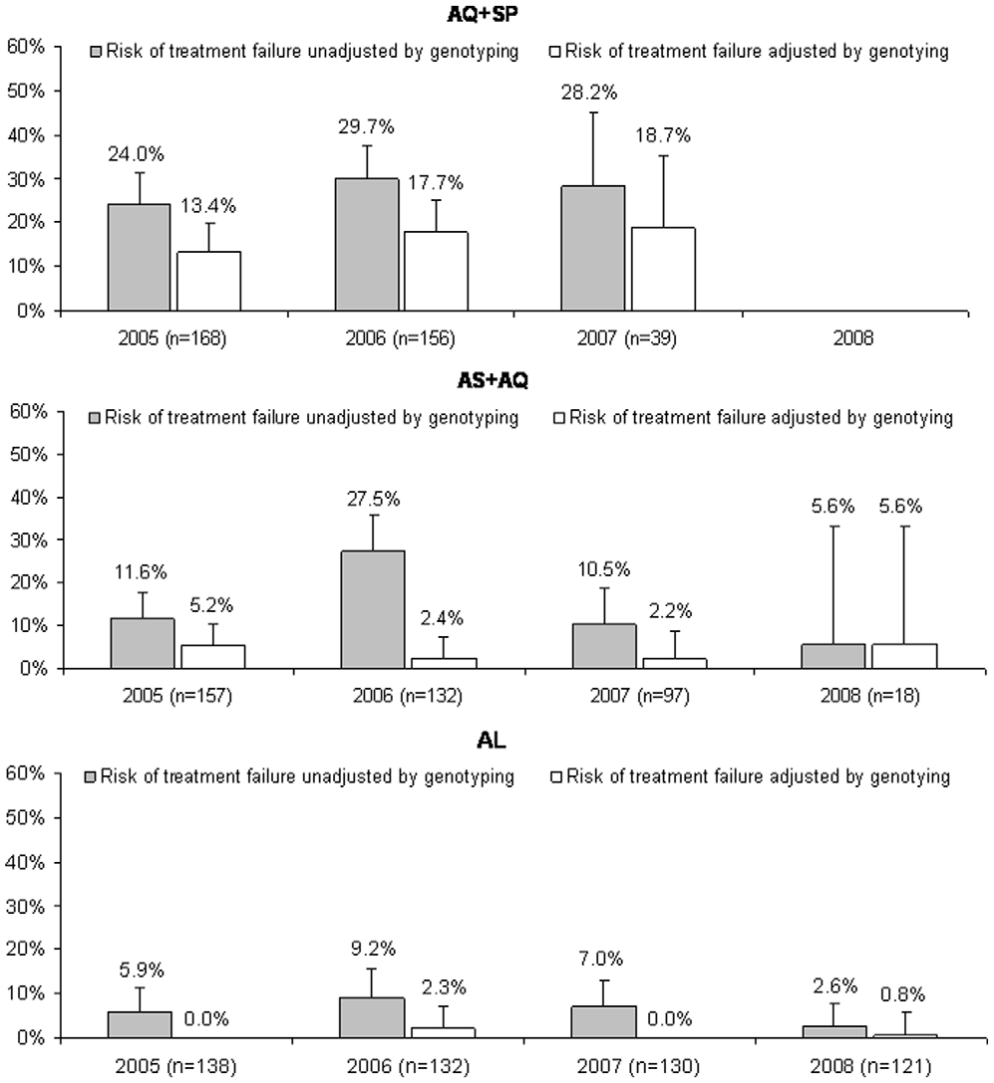
Drug efficacy over time. The annual 28-day risk of failure of treatment of uncomplicated falciparum malaria with the three study regimens, both unadjusted (considering all recurrences) and adjusted by genotyping (considering only recrudescences) is shown. This analysis did not include episodes of malaria that occurred in 2004. Error bars represent upper limits of the 95% confidence intervals.

Final data from our study offer an update over a prior report [12]. Comparisons were over varied intervals, as the AQ/SP treatment arm was discontinued in March 2007 after our interim analysis demonstrated inferior efficacy and the AS/AQ arm was discontinued in January 2008 due to loss of availability of the study formulation of AS. Of the 1464 episodes of malaria 1409 (96%) were uncomplicated malaria treated using one of the 3 study medications (table 1). In addition, 31 treatments with quinine were given for complicated malaria, 12 for uncomplicated malaria after failure of a study drug within 14 days, and 3 for potential adverse events due to study drugs; 9 treatments with quinine plus clindamycin were provided after failure of a course of quinine within 14 days. Genotype-corrected failures at 28 days were uncommon with the two ACT regimens, but significantly more common with AQ/SP (figure 3) [12]. The failure rate with AQ/SP, but not the ACT regimens, increased over time. In a prior analysis this increase appeared to be due to decreasing immunity in our cohort, rather than increasing resistance of parasites to AQ and SP, as the prevalence of known resistance-mediating parasite polymorphisms did not change, but decreases in markers of host immunity were associated with increased risk of failure [20]. As noted previously, parasite clearance was much more rapid with the ACT regimens than with AQ/SP [12]. The proportion of subjects that remained parasitemic 2 or 3 days after the initiation of treatment did not increase over time for any treatment arm. Overall, the efficacies of the ACT study regimens remained excellent (less than 5% rates of genotype-corrected failure) through the course of the study.

### Anemia

Measures of anemia improved through the course of the study. For the 4 full years of observation, the annual overall prevalence of anemia, considering all measurements over the course of the year and anemia defined as hemoglobin <10 gm/dl, decreased from 6.7% in 2005 to 1.0% in 2008 (table 1). Both the increase in mean hemoglobin (0.24 g/dL per year, p<0.001) and decrease in the probability of having hemoglobin <10 gm/dl (relative risk 0.54 per year, p<0.001) were statistically significant. Over the course of the study only 7 of 10,718 hemoglobin measurements were <7 gm/dL (minimum value 6.3 gm/dL).

### Hospital admissions and severe disease

Remarkably, no cases of severe malaria [26] and no deaths occurred during the 4-year duration of our study. A total of 73 hospital admissions occurred, of which 21 were due to malaria. The majority of all admissions and of malaria-related admissions occurred in the first year of the study. All cause hospital admissions decreased from 40 in 2005 to 7 in 2008, and admissions due to malaria decreased from 15 in 2005 to 1–3 per year in 2006–08. The 21 malaria-related hospitalizations, all leading to treatment with quinine, were for single seizures (15 subjects), hyperparasitemia (parasite density greater than 500,000/µL; 3 subjects), inability to sit or stand (2 subjects), and persistent vomiting (1 subject). Therapy with quinine was required for 28 subjects in 2005 (including 14 treatments after a single convulsion, 5 for early treatment failure, and 3 for hyperparasitemia); 10 subjects in 2006 (3 after a single convulsion and 3 for late clinical failure); 5 subjects in 2007 (3 for a single convulsion); and 3 subjects in 2008 (all for a single convulsion).

## Discussion

We followed 690 children in Kampala, Uganda for up to 4 years and identified 1464 episodes of malaria. Our study design included ready access to care for all medical problems. Thus, treatment outside of our study protocol was very uncommon, and we obtained accurate measures of malarial incidence and antimalarial drug efficacy. Children were randomized to one of 3 combination regimens with the first episode of uncomplicated malaria, and we subsequently followed them through up to 30 episodes of malaria. Although further comparisons were limited due to the sequential drop-out of two treatment arms, our results add to our interim analysis [12], showing a clear rank order in the efficacy of our combination treatment arms. In addition, our study offered important insights into the incidence of malaria in Kampala in the context of high quality health care. With available prompt therapy for each episode of malaria and adoption of ITNs, study children experienced remarkable decreases in the prevalence of malarial parasitemia and incidence of malaria over time, and no episodes of severe malaria and no deaths were seen.

Additional efficacy comparisons beyond those previously reported were limited by our need to eliminate the AQ/SP treatment arm due to inferior efficacy in 2007 and the AS/AQ arm when the study formulation of AS was no longer available in early 2008. Consistent with earlier analyses, both ACTs demonstrated excellent efficacy, but AL was superior to AS/AQ [12]. This result is not surprising, as AQ resistance is fairly common in East Africa, and AS/AQ was also inferior to AL in studies from Tanzania [7]–[8] and Republic of Congo [10]. Our results and others suggest that, at least for East Africa, AL is a more appropriate choice than AS/AQ for the treatment of malaria.

We identified remarkable decreases in the incidence of malaria and the prevalences of asymptomatic malarial parasitemia and anemia over the course of our 4 year study. Undoubtedly, these decreases were due to multiple factors. First, a decrease in malarial incidence is expected with increasing age, although in this area of relatively low malaria transmission, changes in incidence with age were modest [19]. Second, we provided ITNs to all study participants in mid-2006. Third, the highly efficacious ACT AL became the standard therapy for uncomplicated malaria in Uganda in 2006. Although AL distribution was limited in Kampala, its use presumably contributed to decreased malaria transmission. Indeed, due to malaria control measures including ITNs and improved therapies, the incidence of malaria has been documented to decrease in some other areas of Africa [2]–[6]. A fourth factor contributing to the dramatic decrease in malarial incidence in our cohort was likely the prompt provision of effective therapy for each episode of malaria. With prompt effective therapy, children were rapidly cured, recrudescences leading to recurrent illness were uncommon, and progression to severe malaria was eliminated. Although interpretations are complex due to multiple changing factors, our results offer support for ready availability of ACTs for the treatment of malaria as a critical component of malaria control efforts.

The most ominous feature of falciparum malaria is progression to severe malaria, with subsequent high mortality. It seems likely that prompt evaluation of febrile illnesses and prompt therapy for all illnesses diagnosed as malaria will decrease the incidence of severe malaria. In our study 1464 episodes of malaria were seen, 21 were complicated, mostly due to single seizures soon after presentation, and no episodes of severe malaria and no deaths were seen. These results further support emphasis on availability of prompt effective therapy for those with malaria as a key control strategy.

It is useful to consider our results from a cohort of children in Kampala in the context of current malaria control efforts across Africa. Resources for malaria control have increased greatly in recent years, and initial gains in control have been seen, with marked decreases in malarial parasitemia and incidence in some areas. At the same time, many areas of Africa continue to have very high levels of malaria transmission and disease incidence. Our results suggest that, in an area of intermediate transmission, prompt therapy with effective agents greatly limits progression to severe disease or death. Thus, distribution of new effective therapies across Africa is a critical priority. Nonetheless, with prompt and effective therapy the incidence of malaria, although decreased, remained quite high in our cohort, and so additional control measures, including ITNs, rational use of insecticides, and, when available, an effective vaccine, are also needed for control of malaria in Africa.

## Supporting Information

Protocol S1 Trial Protocol.
(1.12 MB PDF)

Checklist S1 CONSORT Checklist.
(0.19 MB DOC)
