# Supplementary material for: Incidence of Malaria and Efficacy of Combination Antimalarial Therapies over 4 Years in an Urban Cohort of Ugandan Children
Source: PLoS One. 2010 Jul 30;5(7):e11759. doi: 10.1371/journal.pone.0011759 (PMC2912768; doi:10.1371/journal.pone.0011759)
Supplement: Protocol S1 — Trial Protocol. (1.12 MB PDF) [file pone.0011759.s001.pdf]

LONGITUDINAL COMPARISON OF COMBINATION ANTIMALARIAL THERAPIES IN UGANDAN CHILDREN: EVALUATION OF SAFETY, TOLERABILITY, AND EFFICACY

Funding by:

The National Institutes of Allergy
And Infectious Diseases

Principal Investigator: Philip J. Rosenthal, MD

Funding Source: NIH 1 U01 AI052142

FDA IND Number: 69,577

DMID Protocol Number: 04-068

UCSF-CHR Number: H2397-25789

Version 2.4 October 20, 2008

PROTOCOL CONTENTS

LIST OF ABBREVIATIONS AND ACRONYMS 6

1.0 INVESTIGATORS 7

2.0 FUNDING AGENCY 7

3.0 PARTICIPATING SITES 8

4.0 INSTITUTIONAL REVIEW BOARDS 9

5.0 STUDY PERSONNEL 10

6.0 PROTOCOL SYNOPSIS 11

7.0 STUDY AIM/PURPOSE 13

8.0 BACKGROUND 13

8.1 INTRODUCTION 13
8.2 JUSTIFICATION OF LONGITUDINAL STUDY DESIGN 13
8.3 STUDY DRUGS 14
8.3.1 AMODIAQUINE (AQ) 14
8.3.2 SULFADOXINE-PYRIMETHAMINE (SP) 15
8.3.3 ARTEMISININS 15
8.3.4 LUMEFANTRINE 16
8.3.5.1 Amodiaquine plus Sulfadoxine-Pyrimethamine (AQ+SP) 17
8.3.5.2 Amodiaquine plus Artesunate (AQ+AS) 17
8.3.5.3 Artemether plus Lumefantrine (Co-formulated as Coartemether) 17
8.4 JUSTIFICATION OF MOLECULAR STUDIES 17
8.5. EARLY DATA ANALYSIS AND TRANSITION TO PHASE II 18

9.0 SIGNIFICANCE 18

10.0 TRIAL DESIGN 19

10.1 GENERAL STUDY DESIGN (PHASE I) 19
10.1.1. GENERAL STUDY DESIGN (PHASE II) 19
10.2 STUDY POPULATION 20
10.2.1 STUDY SITE 20
10.2.2 MAPPING OF THE STUDY SITE- COMPLETED 20
10.2.3 PROBABILITY SAMPLING- COMPLETED 20
10.3 SUBJECT RECRUITMENT 20
10.3.1 INITIAL CONTACT METHOD– PHASE I- COMPLETED 20
Figure 1. Subject recruitment 21
10.3.1.1 Initial contact method – Phase II- completed 21
10.3.2 SELECTION CRITERIA – PHASE I- COMPLETED 22
10.3.2.1 Selection criteria – Phase II- completed 22
10.3.3 INITIAL SCREENING CRITERIA- COMPLETED 23
10.4 INFORMED CONSENT 23
10.5 INITIAL SCREENING/BASELINE EVALUATION- PHASE I- COMPLETED 23
10.5.1 INITIAL SCREENING/BASELINE EVALUATION PHASE II-COMPLETED 24

10.6 FINAL ASSESSMENT OF ELIGIBILITY CRITERIA AND ENROLLMENT-COMPLETED ... 24
10.6.1 CHILDREN WITHOUT SYMPTOMATIC MALARIA AT TIME OF INITIAL SCREENING ... 24
10.6.2 CHILDREN WITH SYMPTOMATIC MALARIA AT THE TIME OF INITIAL SCREENING ... 24
10.7 HOUSEHOLD SURVEY- PHASE I- COMPLETED ... 24
10.7.1 HOUSEHOLD SURVEY- PHASE II- COMPLETED ... 24
10.8 RANDOMIZATION ... 25
Figure 2. Subject enrollment ... 26
10.9 TREATMENT ALLOCATION ... 27
10.10 SUBJECT FOLLOW-UP ... 27
10.10.1 INITIAL VISIT ... 27
10.10.2 DIAGNOSIS OF MALARIA ... 28
10.10.3 MANAGEMENT OF UNCOMPLICATED MALARIA ... 28
10.10.3.1 Baseline evaluation ... 28
10.10.3.2 Administration of study medications ... 28
10.10.3.3 Additional treatment ... 28
10.10.3.4 Follow-up for uncomplicated malaria ... 29
Table 1. Follow-up schedule for uncomplicated malaria episodes ... 29
10.10.3.5 Visit windows for follow-up of uncomplicated malaria ... 29
10.10.4 MANAGEMENT OF COMPLICATED MALARIA ... 29
10.10.5 OUTCOME CLASSIFICATION SYSTEM FOR PATIENT MANAGEMENT ... 30
10.10.6 MANAGEMENT OF CLINICAL TREATMENT FAILURES ... 30
10.10.7 MANAGEMENT OF NON-MALARIA ILLNESSES ... 30
10.10.8 AFTER HOURS VISITS ... 30
Figure 3. Management of study participants in the Acute Care Unit ... 31
10.10.9 ROUTINE VISITS- PHASE I- COMPLETED ... 31
10.10.9.1 Routine visits- Phase II ... 32
10.10.9.2 Visit windows for routine visits- Phase I- completed ... 32
10.10.9.3 Visit windows for routine visits- Phase II ... 32
10.10.10 MEDICAL CARE OUTSIDE OF THE STUDY CLINIC ... 32
Figure 4. Subject follow-up ... 33
10.11 SUBJECT WITHDRAWAL CRITERIA ... 33
11.0 STUDY INTERVENTIONS ... 34
11.1 STUDY MEDICATION FORMULATION AND LABELING ... 34
Table 2. Individual Study Medications (for Phase IIa, only AL will be used) ... 34
11.2 STUDY SUPPLY ACQUISITION ... 34
11.3 STORAGE ... 34
11.4 INVESTIGATIONAL DRUG ACCOUNTABILITY ... 34
11.5 BLINDING- PHASE I - COMPLETED ... 34
11.5.1 BLINDING- PHASE II ... 34
11.6 ADMINISTRATION OF STUDY MEDICATIONS ... 34
11.7 OTHER MEDICATIONS ... 35
Table 3. Other medications ... 35
11.8 CONTRAINDICATED DRUGS ... 35
11.9 INSECTICIDE TREATED BED NETS (ITN) ... 35
12.0 DIAGNOSTICS ... 35
12.1 MICROSCOPY ... 35
12.2 CLINICAL LABORATORY STUDIES ... 36
12.3 MOLECULAR STUDIES ... 36
12.4 FUTURE LABORATORY RESEARCH ... 36

12.5 PARASITOLOGY AND IMMUNOLOGY STUDIES – PHASE I ... 36
12.5.1 PARASITOLOGY AND IMMUNOLOGY STUDIES – PHASE IIA ... 37

13.0 DATA COLLECTION AND MANAGEMENT ... 38
13.1 DATA MANAGEMENT ... 38
13.2 DATA QUALITY ASSURANCE AND MONITORING ... 38
13.3 RECORD KEEPING ... 38

14.0 STATISTICAL CONSIDERATIONS ... 38
14.1 PRIMARY OUTCOME ... 38
14.2 SECONDARY OUTCOMES ... 39
14.2.1 DRUG EFFICACY ... 39
14.2.2 SAFETY AND TOLERABILITY ... 39
14.2.3 OTHER LONG-TERM OUTCOMES ... 39
14.3 SAMPLE SIZE CALCULATIONS ... 39
14.4 ANALYTICAL PLAN ... 40
14.4.1 PRIMARY OUTCOME ... 40
14.4.2 SECONDARY OUTCOMES ... 40
14.5 EARLY ANALYSIS OF DATA FROM PHASE I - COMPLETED ... 41

15.0 ADVERSE EVENT MONITORING ... 41
15.1 INTRODUCTION ... 41
15.2 DEFINITIONS ... 42
15.3 BASELINE ASSESSMENT ... 43
15.4 FOLLOW-UP ASSESSMENT ... 43
15.5 REPORTING OF ADVERSE EVENTS ... 43
15.5.1 REPORTING OF SEIZURES ... 44
SAE REPORTING ALGORITHM ... 44
15.6 FOLLOW-UP OF ADVERSE EVENTS ... 45
15.7 STUDY TREATMENT HOLD ... 46
15.8 ROUTINE VISITS ... 46

16.0 DATA AND SAFETY MONITORING ... 46
16.1 DATA AND SAFETY MONITORING BOARD – PHASE I & II ... 47
16.1.1 DATA AND SAFETY MONITORING BOARD – PHASE IIA ... 47
16.2 MONITORING PLAN – PHASE I & II ... 47
16.3 STOPPING GUIDELINES FOR PHASE I- COMPLETED ... 47
Table 5. Stopping rules for results of interim analyses ... 48

17.0 HUMAN SUBJECTS CONSIDERATIONS ... 48
17.1 SUBJECT SELECTION CRITERIA ... 48
17.2 RISKS AND DISCOMFORTS ... 48
17.2.1 PRIVACY ... 48
17.2.2 RISKS OF RANDOMIZATION ... 48
17.2.3 FINGER STICKS AND VENIPUNCTURE ... 48
17.2.4 RISKS OF MEDICATIONS ... 48
17.2.4.1 Introduction ... 48
17.2.4.2 Amodiaquine (AQ) ... 49
17.2.4.3 Sulfadoxine-pyrimethamine (SP) ... 49
17.2.4.4 Artemisinins ... 49
17.2.4.5 Artemether-Lumefantrine (Coartemether) ... 50
17.2.4.6 Combination antimalarial therapy ... 51

Table 6. Adverse Events reported in previous studies for antimalarial study combinations 52
17.2.4.7 Quinine 52
17.2.4.8 Clindamycin 52
17.2.4.9 Acetaminophen 52
17.2.4.10 Mebendazole 53
17.2.4.11 Iron sulfate 53
17.3 TREATMENT AND COMPENSATION FOR INJURY 53
17.4 ALTERNATIVES 53
17.5 COSTS TO THE SUBJECT 53
17.6 REIMBURSEMENT OF SUBJECTS 53
17.7 CONFIDENTIALITY OF RECORDS 53

18.0 QUALIFICATIONS OF INVESTIGATORS 53

19.0 SPECIAL REQUIREMENTS 54

20.0 REFERENCES 54

APPENDICES 60

APPENDIX A	INFORMED CONSENT:	STUDY INFORMED CONSENT	60
		SPECIMEN INFORMED CONSENT	65
APPENDIX A2	INFORMED CONSENT FOR IMMUNOLOGY STUDIES		68
APPENDIX B1	WEIGHT-BASED ADMINISTRATION OF STUDY MEDICATIONS FOR PHASE I		70
APPENDIX B2	WEIGHT-BASED ADMINISTRATION OF STUDY MEDICATIONS FOR PHASE II		73
APPENDIX C.1	CLINICAL OUTCOME CLASSIFICATION		75
APPENDIX C.2	SEVERE FALCIPARUM MALARIA		75
APPENDIX D	GRADING GUIDELINES		76
TABLE I	GUIDELINES FOR GRADING PATIENT SYMPTOMS		76
TABLE II	GUIDELINES FOR GRADING PHYSICAL EXAMINATION SIGNS		77
TABLE III	GUIDELINES FOR GRADING LABORATORY ABNORMALITIES		79
APPENDIX E	EXPECTED ADVERSE EVENTS (AS LISTED IN PACKAGE INSERTS)		80
APPENDIX F	HOME VISITOR SCRIPT FOR INITIAL CONTACT METHOD FOR STUDY RECRUITMENT		82
APPENDIX G	HOUSEHOLD SURVEY		83
APPENDIX H	SAE REPORTING FORM		91
	SERIOUS EVENT REPORTING FORM		93
	SAE/ SERIOUS EVENT REPORTING FORM		95
APPENDIX I	CASE RECORD FORMS		96
	SCREEENING FORM		96
	PHASE II INITIATION FORM		97
	HOSPITAL ADMISSION FORM		98
	HOSPITAL FOLLOW UP FORM		99
	HOUSEHOLD CONTACT FORM		100
	TERMINATION FORM		101
	MALARIA FOLLOW UP FORM		102

	INITIAL VISIT FORM	106
	NON MALARIA FOLLOW UP FORM	108
	ENROLMENT FORM	111
	MALARIA AT ENROLMENT FORM	113
	ROUTINE FORM	116
	ADVERSE EVENT RECORD FORM	117
APPENDIX J	SEIZURE 6-MONTLY SAFETY LOG	118

LIST OF ABBREVIATIONS AND ACRONYMS

AE	adverse event
ACU	Acute Care Unit, pediatric emergency care unit at Mulago Hospital
ALT	alanine aminotransferease
AS	artesunate
AQ	amodiaquine
CBC	complete blood count
CQ	chloroquine
DHFR	dihydrofolate reductase
DHPS	dihydropteroate synthase
DMID	Division of Microbiology and Infectious Diseases (NIH)
DSMB	data safety and monitoring board
GCP	good clinical practices
GPS	global positioning system
IMCI	integrated management of childhood illnesses
ITN	insecticide treated net
IRB	institutional review board
HIV	human immunodeficiency virus
KAP	knowledge, attitude, and practice
MHRA	Medicines and Healthcare products Regulatory Agency (UK)
MoH	Ministry of Health (Uganda Government)
MU	Makerere University (Kampala, Uganda)
NIH	National Institutes of Health (U.S. Government)
PCR	polymerase chain reaction
SAE	serious adverse event
SP	sulfadoxine-pyrimethamine
UCSF	University of California, San Francisco
UNCST	Uganda National Council of Science and Technology
WHO	World Health Organization

1.0 INVESTIGATORS

Principal Investigator/IND Investigator-Sponsor: Philip J. Rosenthal, University of California, San Francisco
Address: UCSF, Box 0811, San Francisco, CA 94143
Phone Number: 415-206-8845
Fax Number: 415-648-8425
Email: prosenthal@medsfgh.ucsf.edu

Co-Investigator: Moses R. Kamya, Makerere University, Kampala, Uganda
Address: Department of Medicine, P.O. Box 7072, Kampala, Uganda
Phone Number: 256-414-533200
Fax Number: 256-414-540524
Email: mkamya@infocom.co.ug
Co-Investigator: Grant Dorsey, University of California, San Francisco
Address: UCSF, Box 0811, San Francisco, CA 94143
Phone Number: 415-206-8845
Fax Number: 415-648-8425
Email: gdorsey@medsfgh.ucsf.edu

Co-Investigator: Sarah G. Staedke, London School of Hygiene & Tropical Medicine
Address: MU-UCSF Research Collaboration, PO Box 7475, Kampala, Uganda
Phone Number: +256-782507132
Fax Number: +256-41540524
Email: sarah.staedke@lshtm.ac.uk

Co-Investigator: Bryan Greenhouse, University of California, San Francisco
Address: UCSF, Box 0811, San Francisco, CA 94143
Phone Number: 415-206-8844
Fax Number: 415-476-9364
Email: bgreenhouse@medsfgh.ucsf.edu

Co-Investigator: Chandy John, Associate Professor, University of Minnesota
Address: Mayo Mail Code 296, 420 Delaware St SE, Minneapolis, MN 55455
Phone Number: 612-624-1966
Fax Number: 612-624-8927
Email: ccj@umn.edu

2.0 FUNDING AGENCY

Institution: The National Institutes of Allergy and Infectious Diseases / Division of Microbiology and Infectious Diseases (NIAID/DMID)
Address: 6610 Rockledge Drive, Bethesda, MD 20892
Contact Persons: Dr. Philip Coyne (Program Officer)

Dr. Mirjana Nesin (Medical Monitor)
Phone Number: 301-451-3024
Fax Number: 301-480-0728
Email: pcoyne@niaid.nih.gov
nesinm@niaid.nih.gov

3.0 PARTICIPATING SITES

Institution: University of California, San Francisco
Address: San Francisco General Hospital, 1001 Potrero Avenue, Building 30, Room 402/408, San Francisco, CA 94110
Contact Person: Peter Padilla
Phone Number: 415-206-5438
Fax Number: 415-648-8425
Email: ppadilla@medsfgh.ucsf.edu

Institution: Makerere University Medical School
Address: MU-UCSF Research Collaboration, PO Box 7475, Kampala, Uganda
Contact Person: Catherine Tugaineyo
Phone Number: 256-414-530692
Fax Number: 256-414-540524
Email: ctugaineyo@muucsf.org

Study Site: MU-UCSF Malaria Research Collaboration Clinic
Address: Mulago Assessment Center, Mulago Hospital Complex, PO Box 7475, Kampala, Uganda
Contact Person: Denise Njama-Meya
Phone Number: 256-41-530380
Email: denise.meya@gmail.com

Clinical Laboratory: MUWRP Makerere University Walter Reed Project
Address: PO Box 16524, Kampala, Uganda
Contact Person: Michael Eller, Leigh Anne Eller, Laboratory Co-Directors
Phone Number: 256-772-221-188
Fax Number: 256-41-534-586

Clinical Laboratory: Ebenezer Limited Clinical Laboratory
Address: Plot 1 Bombo Road, 1st Floor Sure House, PO Box 22096, Kampala, Uganda
Contact Person: Dr. S. Kiyimba Kaggwa, Managing Director
Phone Number: 256-41-342-255
Fax Number: 256-41-230-674

4.0 INSTITUTIONAL REVIEW BOARDS

Institution: Uganda National Council of Science and Technology (UNCST)
Address: Plot 3/5/7 Nasser Road, PO Box 6884, Kampala, Uganda
Contact Person: Leah Nawegulo
Phone Number: 256-414-250499
Fax Number: 256-414-234579

Institution: Makerere University, Research and Ethics Committee (MUREC)
Address: Makerere University, Faculty of Medicine, Office of the Dean,
PO Box 7072, Kampala, Uganda
Contact Person: Dr. Charles Ibingira
Phone Number: 256-414-530020
Fax Number: 256-414-531091

Institution: University of California, San Francisco, Committee of Human Research (UCSF CHR)
Address: Office of Research, Box 0962, San Francisco, CA 94110
Contact Person: Dr. Reese T. Jones
Phone Number: 415-476-1814

Institution: London School of Hygiene and Tropical Medicine, Ethics Committee
Address: Keppel Street, London WC1E 7HT
Contact Person: Professor Tom Meade
Email: Ethics@lshtm.ac.uk

5.0 STUDY PERSONNEL

Name	Title	University
Philip J. Rosenthal, MD	Principal Investigator	UCSF
Moses R. Kamya, MBChB, MMed, MPH	Co-Investigator	MU
Grant Dorsey, MD, PhD	Co-Investigator	UCSF
Sarah G. Staedke, MD	Co-Investigator	LSHTM
Jeffrey N. Martin, MD, MPH	Co-Investigator	UCSF
Lisa Butler, PhD	Co-Investigator	UCSF
Bryan Greenhouse, MD	Co-Investigator	UCSF
Chandy John, MD, MS	Co-Investigator	U.Minnesota
Tamara Clark, MHS	Project Coordinator	UCSF
Denise Njama, MBChB, MSc	Senior Study Coordinator	MU
Bridget Nzarubara, MBChB	Junior Study Coordinator	MU
Catherine Maiteki, MBChB	Junior Study Coordinator	MU
Joaniter Nankabirwa, MBChB	Study Physician	MU
Pascal Kitwonda, MBChB	Study Physician	MU
Immaculate Ampiere, MBChB	Study Physician	MU
Leatitia Kampiire, MBChB	Study Physician	MU
Levy Mugenyi	Data Manager	MU
Richard Bariyge	Data Entry Officer	MU
Basaliza M. Karakire, RN	Senior Study Nurse	MU
Naome Kilama, RN	Study Nurse	MU
Norah Asaba, RN	Study Nurse	MU
Sam Nsobya, MSc	Senior Lab Technologist	MU
Regina Nakafeero	Senior Lab Technologist	MU
Moses Kiggundu	Lab Technologist	MU
Christopher Bongole	Lab Technician	MU
Maxwell B. Kilama	Lab Technician	MU
Felix Jurua	Lab Technician	MU
Marx Dongo	Director of Community Services	MU
William Musoke	Home Visitor/Nurse	MU
Caroline Bako	Home Visitor/Nurse	MU
Peter Padilla	Administrator	UCSF
Catherine Tugaineyo	Administrator	MU
Kenneth Mwebaze	Administrative Assistant	MU

6.0 PROTOCOL SYNOPSIS

**Title**	**Longitudinal comparison of combination antimalarial therapies in Ugandan children: evaluation of safety, tolerability, and efficacy**
**Description**	Randomized, single-blinded, longitudinal clinical trial comparing different combination regimens for treatment of uncomplicated malaria.
**Clinical Site**	The study will be conducted in Kampala, Uganda at the Makerere University – University of California, San Francisco (MU-UCSF) malaria study clinic, which was established in 1998. The study clinic is located in the outpatient department of Mulago Hospital, the primary referral hospital in Uganda. The study clinic is open daily from 8:30 am to 5:00 pm and after-hours care is available 24 hours at the Acute Care Unit, the pediatric unit of Mulago Hospital. Related molecular studies will be conducted at affiliated laboratories at Makerere University and at the University of California, San Francisco.
**Malaria Case Definitions**	Uncomplicated malaria (all of the following)<br>1. Fever (> 38.0ºC tympanic) or history of fever in the previous 24 hours<br>2. Positive thick blood smear<br>3. Absence of complicated malaria<br>Complicated malaria (any of the following)<br>1. Evidence of severe disease with a positive thick blood smear<br>2. Danger signs with a positive thick blood smear<br>3. Parasite density > 500,000/ul

Phase I. (Enrollment November 2004 – April 2005)

**Participants and Sample Size**	Representative sample of 600 Ugandan children aged 1 to 10 years living in the Mulago III parish of Kampala.
**Selection Criteria**	***Inclusion criteria***<br>1. Age 1 to 10 years<br>2. Agreement to come to the study clinic for any febrile episode or other illness<br>3. Agreement to avoid medications administered outside the study<br>4. Willingness of parents or guardians to provide informed consent<br>***Exclusion criteria***<br>1. History of any known serious chronic disease requiring frequent medical attention (e.g. AIDS, sickle cell disease, malignancy)<br>2. Intention to move from Kampala during the follow-up period<br>3. Any history of serious side effects to study medications<br>4. Weight < 10 kg<br>5. Severe malnutrition<br>6. Life-threatening screening laboratory test result
**Treatment Intervention**	Uncomplicated malaria: Treatment with pre-assigned combination regimen (amodiaquine + sulfadoxine/pyrimethamine, amodiaquine + artesunate, or artemether + lumefantrine, each for 3 days)<br>Complicated malaria or treatment failure: Quinine (10 mg/kg TID x 7d)
**Follow-up and Diagnosis of Malaria**	Participants will be followed for 4 years for all of their routine medical care in our study clinic. Routine home visits will be done on any participant not seen in our study clinic after any consecutive 30-day period. Patients presenting with a new episode of fever will undergo standard evaluation for the diagnosis of malaria. For each participant, all episodes of uncomplicated malaria will be treated with the same treatment regimen, randomly assigned at the time their first episode is diagnosed.
**Primary outcome**	Treatment incidence density (treatments per time at risk) for each treatment arm
**Secondary outcomes**	**Short-term (14-day):** (1) clinical and parasitological outcome, (2) rates of fever and parasite clearance, (3) change in hemoglobin level from day 0 to 14, (4) presence of gametocytes following treatment, and (5) safety and tolerability of study medications<br>**Long-term (beyond 14-day)**: (1) risk of recrudescence and (2) risk of reinfection using Kaplan-Meier product limit estimates of risk at various time intervals, (3) change in hemoglobin level, and (4) safety and tolerability of study medications<br>**Longitudinal:** (1) prevalence of asymptomatic parasitemia and (2) mean hemoglobin

Phase II. (May 2006 – January 2008), Phase IIa. (February 2008 – April 2009)

**Participants and Sample Size**	Children currently enrolled in Phase I of the study (approximately 550) continued with Phase II of the study beginning in May 2006. New study participants aged 1 to 10 years living in the same households as current study participants (90 children) were enrolled in Phase II of the study beginning upon approval of protocol version 2.1.
**Selection Criteria**	***Inclusion criteria***<br>1. Child and Guardian/Parent are from a household currently in the study<br>2. Age 1 to 10 years<br>3. Agreement to come to the study clinic for any febrile episode or other illness<br>4. Agreement to avoid medications administered outside the study<br>5. Willingness of parents or guardians to provide informed consent<br>***Exclusion criteria***<br>1. History of any known serious chronic disease requiring frequent medical attention (e.g. AIDS, sickle cell disease, malignancy)<br>2. Intention to move from Kampala during the follow-up period<br>3. Any history of serious side effects to study medications<br>4. Weight < 10 kg<br>5. Severe malnutrition<br>6. Life-threatening screening laboratory test result
**Initiation of Phase II**	Distribution of insecticide treated nets (ITNs) was conducted in May-June, 2006. For study participants enrolled in Phase I of the study, Phase II began the day they were given ITNs. New study participants enrolled in Phase II of the study were given ITNs on the day of enrollment. One additional ITN per household was also distributed.
**Phase II. Treatment Intervention**	Uncomplicated malaria: Treatment with pre-assigned combination regimen (amodiaquine + artesunate or artemether + lumefantrine, each for 3 days)<br>Complicated malaria or treatment failure: Quinine (10 mg/kg TID x 7d)
**Phase IIa. Treatment Intervention**	Uncomplicated malaria: Treatment with artemether + lumefantrine, for 3 days<br>Complicated malaria or treatment failure: Quinine (10 mg/kg TID x 7d)
**Follow-up and Diagnosis of Malaria**	Participants enrolled in Phase I of the study will complete 4 years of follow-up (extended one year beyond the originally planned 3 years). Participants enrolled in Phase II of the study will be followed until the last child enrolled in Phase I of the study from the same household has completed follow-up. Routine home visits will be done on any participant not seen in our study clinic after any consecutive 30-day period. Patients presenting with a new episode of fever will undergo standard evaluation for the diagnosis of malaria. For each participant, all episodes of uncomplicated malaria will be treated with the same treatment regimen, randomly assigned at the time their first episode is diagnosed. Beginning on February 1, 2008, all participants will receive artemether + lumafantrine for uncomplicated malaria episodes.
**Primary outcome**	Treatment incidence density (treatments per time at risk) for each treatment arm
**Secondary outcomes**	**Short-term (14-day):** (1) clinical and parasitological outcome, (2) rates of fever and parasite clearance, (3) change in hemoglobin level from day 0 to 14, (4) presence of gametocytes following treatment, and (5) safety and tolerability of study medications<br>**Long-term (beyond 14-day)**: (1) risk of recrudescence and (2) risk of reinfection using Kaplan-Meier product limit estimates of risk at various time intervals, (3) change in hemoglobin level, and (4) safety and tolerability of study medications<br>**Longitudinal:** (1) prevalence of asymptomatic parasitemia and (2) mean hemoglobin

7.0 STUDY AIM/PURPOSE

Phase I of this study was a randomized, single-blinded longitudinal clinical trial, comparing the efficacies of different combination antimalarial regimens for the treatment of uncomplicated malaria in a cohort of Ugandan children. The clinical study is linked to an epidemiological survey and molecular analyses of parasite and human genetic polymorphisms. We hypothesize that longitudinal evaluation will identify important differences between the efficacies and selective pressures of leading antimalarial combination therapies even if short-term activities are similar. We further hypothesize that identifiable parasite and host polymorphisms will help predict outcomes after antimalarial therapy and that the characterization of these polymorphisms will identify factors that contribute to malaria incidence and responses to therapy. To test these hypotheses, the aims of the study are: 1) to compare the safety, tolerability, and efficacy of combination antimalarial therapies using a longitudinal design, 2) to follow plasmodial genetic polymorphisms as longitudinal markers of antimalarial drug resistance, and 3) to evaluate the roles of host genetic polymorphisms in antimalarial drug resistance and the incidence of clinical malaria.

In Phase II, the study was changed to a randomized, open-label longitudinal clinical trial, comparing two combination antimalarial regimens (amodiaquine+artesunate (AQ+AS) and artemether+lumefantrine (AL, Coartem). In addition, all study participants received an insecticide treated bed net (ITN). The study aims were unchanged.

In Phase IIa, the AQ+AS treatment arm will be discontinued. This change is necessitated by the lack of availability of artesunate (Arsumax™) after January, 2008, a change that follows WHO calls to eliminate artemisinin monotherapies due to worries regarding selection of drug resistant parasites. AQ+AS is now available as a co-formulated product, but this product is not FDA-approved, is not produced according to good manufacturing practices, and would necessitate changes in dosing for the two components of the drug. As approval of AQ+AS is not possible in a timely manner, the combination will be discontinued. All participants will receive AL for episodes of uncomplicated malaria. Although no longer a drug efficacy comparison, our study will have continued value to study the safety of AL, the most widely recommended artemisinin-based combination therapy in Africa, to provide parasites for laboratory correlation with clinical measures of drug resistance, and to serve as a control for a parallel study of malaria in HIV-infected children in Kampala, which also treats malaria with AL.

8.0 BACKGROUND

8.1 Introduction

Malaria is one of the most important infectious diseases worldwide. In Africa, which bears the greatest burden of this disease, control efforts have been largely unsuccessful. Major factors contributing to this lack of success have been the necessary reliance of malaria control on effective therapy and the increasing resistance of parasites to available drugs. New therapies are urgently needed, and it is generally agreed that combination therapy for uncomplicated malaria offers the best opportunity for effective therapy and for the prevention of selection of resistant parasites. However, there is no consensus on the optimal combination regimen or the best study design for comparing the safety, tolerability, and efficacy of antimalarial regimens.

8.2 Justification of longitudinal study design

The study of malarial therapy poses a unique challenge in much of Africa, where the disease is highly endemic, often resulting in several clinical attacks per year, especially in children [1]. Additionally, antimalarial drug resistance is often detected quite late after therapy, such that patients are at risk for

treatment failure several weeks after therapy is initiated [2]. Patients are also continuously at risk for disease due to new infections. Thus, African children are often repeatedly treated for malaria over a relatively short period of time. Typically, studies of antimalarial drug efficacy have focused on individual episodes of disease, and limited follow-up to two to four weeks. However, recent results from our group and others suggest that traditional short-term evaluations of efficacy are not adequate to estimate the true impact of drug resistance, particularly for longer-acting agents and more effective combination therapies [3]. Rather, important differences may only be apparent after extended evaluation. Longitudinal evaluation also provides a “real world” comparison of different regimens, with assessments of the effects of repeated dosing on malarial incidence, selection of drug-resistant parasites, and drug toxicity. Longitudinal evaluation benefits from new molecular techniques, which allow the accurate assessment of treatment responses and help characterize the roles of parasite and host polymorphisms in disease incidence and responses to therapy.

In addition, our experience indicates there are important practical advantages to the use of a longitudinal design for our study population. Management of malaria in our urban population is characterized by the utilization of a wide range of health services, including frequent self-treatment at home. The longitudinal design will enable us to maximize compliance with our study protocol and minimize the use of non-study antimalarial therapy. We will also be able to collect valuable prospective data on disease incidence and other important epidemiological measures. Lastly, the longitudinal data set will provide a valuable collection of reagents for molecular epidemiology studies. In our previous longitudinal study with a cohort of 316 children, we diagnosed 577 episodes of malaria, with successful outcome classification in over 99% of episodes [3]. This study has already provided valuable insights into the true efficacies of studied drugs, the epidemiology of malaria in Kampala, and the molecular epidemiology of antimalarial drug resistance.

8.3 Study drugs

In Phase I of this study, we studied the safety, tolerability, and efficacy of three combination therapies that have recently been adopted as first-line treatment of uncomplicated malaria in Africa. Children recruited from the community were randomized to one of three treatment regimens at the time of their first episode of uncomplicated malaria. Study participants received the same treatment regimen for all subsequent episodes of uncomplicated malaria diagnosed during the study period. Treatment arms included the following combination regimens:

1. amodiaquine plus sulfadoxine-pyrimethamine (AQ+SP)
2. amodiaquine plus artesunate (AQ+AS)
3. artemether plus lumefantrine (AL, co-formulated as coartemether, Coartem)

Beginning in Phase II, amodiaquine plus sulfadoxine-pyrimethamine (AQ+SP) was discontinued as a treatment arm for uncomplicated malaria. Data analysis provided for a meeting of the DSMB in July 2006 found this regimen to be less efficacious when compared to the other two treatment arms, AQ+AS and artemether + lumefantrine (Coartem). Based on recommendations of the DSMB and sponsor, children in the AQ+SP arm were re-randomized to one of the other two treatment arms. The study continued as an open-label clinical trial of two treatment arms. The use of placebo was discontinued. For Phase IIa, AQ+AS will be discontinued on February 1, 2008 due to the discontinuation of the AS product.

8.3.1 Amodiaquine (AQ)
Use of this drug will be discontinued with the start of Phase IIa.

Amodiaquine (AQ) is very similar in structure to chloroquine (CQ), and the two drugs probably share mechanisms of action. However, AQ routinely retains activity for the treatment of uncomplicated malaria in settings where clinical CQ treatment failures are very common [4]. Use of AQ has been

limited since the 1980's due to concerns over toxicity, primarily hepatotoxicity and blood dyscrasias in individuals receiving long-term chemoprophylaxis [4]. However, AQ has remained available and inexpensive in Africa, and it has been fairly widely used as a replacement for CQ. Recent studies have suggested that AQ is, indeed, efficacious and safe when used for the treatment of uncomplicated malaria, even in areas of high-level CQ resistance [4-8] (see Section 17). At our study site in Uganda, no serious toxicities were observed with AQ monotherapy (131 treatments). Efficacy with AQ monotherapy was markedly superior compared to previous studies of CQ in the same target population (14-day treatment failure rate 7% vs. 47%) [8, 9]. Based on data from multiple studies showing limited toxicity and marked improvement in efficacy over CQ, AQ is now increasingly advocated, alone or in combination regimens, as an appropriate low-cost alternative for CQ in the treatment of uncomplicated malaria in Africa.

8.3.2 Sulfadoxine-pyrimethamine (SP)

Use of this drug was discontinued with the start of Phase II.

Sulfadoxine-pyrimethamine (SP), a synergistic combination of a dihydrofolate reductase (DHFR) inhibitor (pyrimethamine) and a dihydropteroate synthase (DHPS) inhibitor (sulfadoxine), has been widely used for the treatment of uncomplicated malaria in Africa. SP benefits include a single-dose treatment regimen and low cost, but it is relatively slow-acting and has been subject to increasing resistance. As with AQ, the use of SP for chemoprophylaxis was discontinued due to rare but serious dermatological toxicity. However, the serious toxicities seen with chronic therapy appear to be very rare with routine short-term use of SP (see Section 17). Due to its availability, low cost, and ease of use, SP has generally been the preferred replacement for CQ for the treatment of uncomplicated malaria in Africa [10]. Several African countries, including Malawi, Kenya, and Tanzania, have already changed their recommendation for the first-line treatment of uncomplicated malaria from CQ to SP. However, SP resistance already appears to be fairly common in many countries in East Africa, and there is growing concern that its useful therapeutic lifespan will be short [11]. In our recent longitudinal study in Uganda, SP monotherapy failed after 14 days in 18% of children treated for uncomplicated malaria, but long-term failure rates were actually much higher (31% at 28 days and 38% at 42 days, both adjusted for true recrudescence by genotyping) [3]. Based on data from multiple sites, an emerging consensus argues that combination regimens, perhaps including SP, but not SP monotherapy, should be standard for the treatment of malaria. The recommendation for combination therapy is particularly appropriate for regions, such as East Africa, where high levels of resistance to SP are already present. Recently, four countries in Africa (Uganda, Ethiopia, Eritrea, and Zimbabwe) have selected the combination of CQ+SP and three countries (Rwanda, Mozambique, and Senegal) have selected the combination of AQ+SP as first-line therapy [12]. Of note, the AQ+SP arm of the study is being dropped with this protocol revision, and SP will no longer be received by study participants in Phase II of the study.

8.3.3 Artemisinins

Artemisinins are a relatively new class of antimalarials that are natural products or semi-synthetic compounds derived from the plant *Artemesia annua* [13]. The artemisinins offer very rapid and potent treatment for malaria, and they benefit from a lack of known drug resistance. However, artemisinin derivatives are expensive, and because of their short half-life ideally should be given for extended courses or in combination with other drugs. Concerns have been raised about the safety of artemisinins due to evidence of neurotoxicity in animals, primarily associated with large parenteral doses of oil-soluble artemisinins (artemether and arteether). However, oral doses of water-soluble compounds, such as artesunate (AS), appear to be very well tolerated and coartemether, an orally administered combination regimen, which includes artemether, has a good safety profile (see Section 17). Artemisinins have been widely used in Southeast Asia [14-16] where effective regimens generally combine these short-acting agents with another compound.

Careful consideration of available efficacy and toxicity information has led to a broad recommendation for orally administered artemisinins in combination with another agent as standard

therapy for the treatment of uncomplicated malaria in Africa [17-20]. Nine countries in Africa (Cameroon, Equatorial Guinea, Ghana, Liberia, Sao Tome and Principe, Sierra Leone, Burundi, Gabon, and Zanzibar) have selected the combination of AQ+AS as first-line therapy [12], and Benin, Ethiopia, Kenya, Uganda, Tanzania, South Africa, Zambia, and Comoros have selected coartemether, although implementation of these guidelines may be complicated by cost. Indeed, many experts now advocate immediate widespread use of artemisinins as components of combination first line antimalarial therapy [21]. Despite the emerging consensus that artemisinin-containing combination regimens are the treatment of choice for malaria in Africa, there remain concerns about efficacy (treatment courses are commonly followed by late recrudescence or new infections [3]) and potential toxicity. One major value of this study will be the opportunity to definitively test the long-term efficacy and safety of artemisinin combination regimens.

8.3.4 Lumefantrine

Lumefantrine (also known as benflumetol) is an aryl alcohol that is related to halofantrine. Halofantrine is available for antimalarial therapy in Africa, but it is very expensive, and its use is also limited by reports of rare severe cardiac toxicity. Lumefantrine is a highly effective antimalarial that appears to be well tolerated, and does not appear to cause the cardiac toxicity seen with halofantrine [22-24]. However, data on the use of the drug as monotherapy are limited [25]. Lumefantrine is available as a component of coartemether, in combination with artemether. Most available safety data are for the combination of lumefantrine and artemether (Section 17).

8.3.5 Combination antimalarial therapy

Combination therapy has become the standard for the treatment of many infectious diseases. In general, the advantages of combination therapy are improved efficacy and decreased selection of resistant organisms. Two infections for which combination therapy is the standard of care are tuberculosis and HIV infection. For tuberculosis, it was seen by the early 1950's that single-drug therapy was commonly followed by the selection of resistant organisms. Combinations of anti-mycobacterial drugs are required to achieve high rates of cure. Standard regimens include isoniazid and rifampin, usually with additional agents. For the treatment of HIV, initial antiretroviral drugs offered some successes in the 1980's, but by the mid-1990's it was clear that the selection of resistant virus was very common after monotherapy, and that this selection could often be prevented by combination therapy. At present, the standard of care for advanced HIV infection includes therapy with multiple antiretroviral agents.

Combination antimalarial therapy has already become standard in some areas. AS plus mefloquine is the standard regimen for falciparum malaria in areas of Thailand with high-level drug resistance [17, 18]. However, the safety and efficacy of potentially appropriate and less expensive antimalarial combinations have been under-studied. There is broad consensus that combination therapies offer the optimal means of treating malaria in Africa, and many insist that all new antimalarial regimens should include multiple drugs [20]. Many specifically advocate combination regimens including artemisinins, but cost may preclude widespread use of these regimens [26], and their efficacy has not yet been widely studied. The WHO currently recommends use of combination therapy for treatment of uncomplicated malaria in areas with drug resistance, and considers coartemether, AQ+AS, AS+SP, and AQ+SP to be appropriate therapeutic options [27].
Of note, SP is technically a drug combination, but since its efficacy appears to be dependent on synergism between the two components, it loses effectiveness once resistance to either drug develops. SP is usually considered a single agent in considerations of combination antimalarial therapy.

In 2000, the Uganda Ministry of Health (MoH) selected the combination of CQ+SP to replace CQ as first-line treatment for uncomplicated malaria. Studies have since been conducted in Uganda to evaluate the efficacy and safety of CQ+SP, and to compare this regimen to potential alternative

combinations for first-line therapy (Uganda Malaria Surveillance Project, unpublished data). Available data were recently evaluated at a national consensus meeting to review Uganda's antimalarial drug policy. Given substantial evidence that CQ+SP was failing, a decision was made to abandon this as the first-line regimen, and artemether-lumefantrine (coartemether) was provisionally adopted as the new first-line treatment for uncomplicated malaria. AQ+AS and AQ+SP may also have a role in the revised drug policy.

8.3.5.1 Amodiaquine plus Sulfadoxine-Pyrimethamine (AQ+SP)
This combination was discontinued with start of Phase II.

We recently showed that CQ+SP was significantly inferior to AQ+SP for the treatment of uncomplicated malaria in Kampala [28, 29]. AQ+SP is a combination of two currently available and inexpensive drugs, and in three studies conducted at our site in Uganda [3, 8, 28] and in studies from Tanzania [30] and Rwanda [31], AQ+SP showed excellent efficacy. In our prior longitudinal study, with measurements of either long-term success of individual therapies or of malaria treatment incidence density over one year, the efficacy of AQ+SP was significantly superior to that of SP monotherapy and to that of AS+SP (which performed well initially, but had many failures after 14 days) [3]. In our three published studies from Kampala, no serious toxicity was seen after 438 treatments with AQ+SP [3, 8, 28]. In a more recent study, which evaluated an additional 132 patients treated with AQ+SP, the regimen was well-tolerated by most patients (Staedke, unpublished data). However, a few concerning adverse events were identified in AQ+SP patients, although the association between these events and the study treatment was not clear (Section 17). As noted above, the AQ+SP arm will be discontinued in Phase II of the study.

8.3.5.2 Amodiaquine plus Artesunate (AQ+AS)
This combination was discontinued with start of Phase II.

In a recent comparative study conducted in Kampala, AQ+AS was well-tolerated and showed superior efficacy over CQ+SP, with the added benefit of rapid action associated from the artemisinin component. Compared to AQ+SP, AQ+AS had a lower risk of clinical treatment failure but a higher risk of reinfection (Staedke, unpublished data). In addition, a multi-center study of 470 treatments with AQ+AS at sites in Kenya, Senegal, and Gabon showed that the regimen provided excellent efficacy for uncomplicated malaria and was well tolerated [32].

8.3.5.3 Artemether plus Lumefantrine (Co-formulated as Coartemether)
Coartemether is an oral preparation containing the artemisinin derivative, artemether, and lumefantrine (previously known as benflumetol). It is currently the only co-formulated combination of unrelated antimalarial drugs available for use in Africa and is on the WHO Essential Drugs List [24]. As with other artemisinins, artemether is characterized by rapid antimalarial action, however, recrudescence is frequent when artemether is provided as a single agent, unless given for at least 5-7 days [33, 34]. Lumefantrine also has a high cure rate, but parasite and fever clearance is slower than with artemether [22]. Coartemether benefits from coformulation, to improve compliance, and is a highly effective antimalarial.

8.4 Justification of molecular studies

Our primary outcome will be a clinical endpoint (treatment incidence density) and power calculations are based only on this endpoint. Molecular genotyping is required to categorize clinical outcomes, and so these studies will be a core component of our drug efficacy analysis. In addition, the longitudinal study affords us an excellent opportunity to study associations between parasite and host genetic polymorphisms and malaria. Polymorphisms in certain parasite genes (pfcrt, pfmdr1, dhfr, dhps) have been associated with treatment responses to aminoquinolines and antifolates, but the importance of different mutations remains incompletely understood. Additional polymorphisms may impact upon

host responses, and therefore potentially on the incidence of malaria. Polymorphisms in some host genes (globin, glucose-6-phosphate dehydrogenase) and promoters (tumor necrosis factor-α, inducible nitric oxide synthase) are associated with severe malaria, but associations with the incidence of malaria or drug responses are uncertain. We will evaluate polymorphisms in parasite genes, including those noted above, to search for associations between genotypes and treatment responses. We will evaluate host polymorphisms of interest, including those noted above, and evaluate associations between host polymorphisms and both the incidence of malaria and responses to antimalarial therapy.

8.5. Early data analysis and transition to Phase II

Phase II of the study incorporated six major changes, including early analysis of efficacy and safety data, distribution of insecticide-impregnated bednets to all study subjects starting in May 2006, recruitment of additional young children from current study households, discontinuation of one treatment arm (AQ+SP), and changing to an open-label study design and re-consent of all current study participants. Each change was justified by current malaria research needs and standard-of-care, as follows.

With rapidly changing malaria treatment recommendations and practices in Africa, it is imperative that drug efficacy and safety data be shared with public health authorities as expeditiously as possible. This is particularly true in the case of the three regimens compared in this trial, all of which have important current roles in the treatment of uncomplicated malaria in Africa. In Phase II we unblinded treatment assignments, completed analyses by comparing genotypes of samples from recurrent infections with those at presentation, and determined the comparative efficacies and safeties of the three antimalarial treatments for purposes of sharing with the Uganda Ministry of Health and publication.

With the initiation of Phase II we supplied bed-nets to all study participants. The provision of bed-nets has become the standard of care in Africa, and so it was deemed ethically inappropriate to continue to withhold them. The implementation of bednets in our study population likely decreased malaria incidence in all study arms. However, as different treatments differentially impact upon the risk of new infection after treatment, the effects of bednets on subjects in different treatment arms may differ, and it was of value to compare relative efficacies after this intervention. In addition, the implementation of bednets allowed comparison of overall malaria incidence in our population before and after bednet use, although this comparison was necessarily limited by the use of historical controls.

With Phase II we also re-consented all current study participants and re-opened enrollment to children, aged 1 to 10 years, belonging to a household that already had a child enrolled in the study. This change was considered appropriate, as our cohort is aging, and it was important to include younger children, the group at greatest risk of malaria, in our Phase II analysis. Enrollment is now complete; no additional enrollment of study subjects will take place in Phase IIa.

Based on our interim analysis and guidance from our DSMB, the AQ+SP treatment arm, which was less efficacious than the other two arms, was dropped with the start of Phase II. With unblinding of study personnel for the purpose of data analysis and with increasing patient awareness of the appearance of study drugs, continued blinding ~~is~~ was not possible, and the study was continued with an open-label design.

9.0 SIGNIFICANCE

Malaria is one of the most important infectious diseases, and its control is greatly threatened by drug resistance. Current methods for evaluating drug efficacy, including assessing outcome 14 days after treating a single malaria episode, likely significantly underestimate the level of drug resistance.

African countries are being forced to revise antimalarial drug policy in the absence of adequate data on the efficacy and safety of candidate regimens for first-line therapy. Assessment of the long-term implications of antimalarial drug resistance, evaluation of new combination regimens, and the molecular evaluation of the role of parasite and host genetic polymorphisms in drug resistance are all important research needs that will be addressed in this study.

10.0 TRIAL DESIGN

(Note: Text referring to older phases of the study is unchanged from earlier versions. With this protocol version 2.2, we enter Phase IIa).

10.1 General study design (Phase I)

This will be a randomized, single-blinded, longitudinal clinical trial comparing the safety, tolerability, and efficacy of three different combination antimalarial regimens for the treatment of uncomplicated malaria. The clinical study will recruit participants from a defined and mapped source population, conduct a survey of epidemiological factors on each study participant, and follow clinical care and outcomes for this cohort over an extended period of time. Molecular analyses of parasite and human genetic polymorphisms will evaluate the impact of parasite mutations on treatment efficacy, the effects of repeated treatments on selection for resistance-mediating genotypes, and the impact of host polymorphisms on the incidence of malaria and responses to therapy.

Probability sampling of a census of households within a geographically defined community will be used to select a random sample of 600 Ugandan children between the ages of 1 to 10 years. Participants will be followed for 3 years for all routine medical care in our study clinic at Mulago Hospital. All patients presenting to our study clinic with a new episode of fever will undergo standard evaluation (history, physical examination and Giemsa-stained blood smear) for the diagnosis of malaria. Participants will be randomized to one of three combination treatment regimens at the time of their first diagnosis of uncomplicated malaria. All subsequent episodes of uncomplicated malaria will be treated with each participant's assigned treatment regimen. All clinical treatment failures occurring within 14 days of diagnosis and all episodes of complicated malaria will be treated with quinine, the standard therapy for malaria after treatment failure in Uganda. All episodes diagnosed more than 14 days after a previous episode will be considered new episodes for treatment purposes. Study investigators involved in patient evaluation and treatment outcome classification will be blinded to treatment assignment. Study subjects will not be informed of their treatment assignments. Dosing regimens will be the same for all treatment groups, including the use of placebo tablets where applicable. The taste and color of study medications will not be identical. Only nurses responsible for administration of study drugs will remain unblinded to participant's assigned treatment regimens. Routine home visits will be made for any participant not seen in our study clinic after any consecutive 30-day period. Routine home visits will be made every 90 days and will include review of study protocol with study participants, assessment for any outside medical care, a focused history and physical examination and routine laboratory testing.

10.1.1. General Study Design (Phase II)

Following the first year of follow up, recruitment will be re-opened for children belonging to the same households previously recruited to the study, for children not previously enrolled aged 1 to 10 years. Also at this time, all participants will receive an insecticide treated bed net, instructions will be given to the guardians on its use, and phase II follow up will begin. All other aspects of subject evaluation, management, and follow-up will be the same as in Phase I, except that we will discontinue every 30 day finger sticks (subjects will still be visited if they have not been seen in the clinic for 30 days, but finger sticks for blood smears and filter paper will only be performed during clinic evaluations, when clinically indicated or if the patient has not been evaluated in the clinic over a 90 day period).

Upon signing a new consent form, children not previously randomized will be randomized to two study treatment arms, amodiaquine + artesunate (AQ+AS) and Coartemether (AL), upon their first episode of uncomplicated malaria. All children previously assigned to the amodiaquine + sulfadoxine-pyrimethamine (AQ+SP) treatment arm will be re-randomized to one of the other two treatment arms. In Phase II, the study will be open-label, placebos will not be used, and study staff and participants will not be blinded to treatment assignments. Beginning on February 1, 2008 (Phase IIa), AQ+AS will be dropped due to the discontinuation of the AS product and all episodes of uncomplicated malaria will be treated with AL.

10.2 Study population

10.2.1 Study site

The study population will be recruited from the Mulago III Parish within the Kawempe District, the region in which our study clinic at Mulago Hospital is located. Information on the Mulago III Parish comes from preliminary 2001 national census data and an urban planning report recently completed by the Department of Geography at Makerere University. Mulago III has an estimated population of 12,411 (3845 households) and is divided into five zones covering 226 acres. The area is primarily residential, characterized by high population density with low-income, single- or two-room housing units. The local economy is primarily dependent on petty commercial activities and small-scale subsistence farming. The Mulago III Parish has a large swamp area with poor drainage and frequent flooding during the rainy seasons. In a previous community based study, this parish had a high incidence of malaria with over 2 treatments per person year in children under the age of five years [35]. Local health care facilities are limited to one maternity home and 7 private clinics. Mulago Hospital is 200 meters away and is the source of health care for most residents.

10.2.2 Mapping of the study site- completed

All households in our geographically defined population (Mulago III Parish) will be enumerated to provide a sampling frame for the selection of children to be included in the study. A map of the Mulago III Parish has been produced previously by our research group, detailing boundaries, roads, footpaths, and pertinent landmarks. Using this map as a reference, study investigators will cover the entire area within the boundaries of the Mulago III Parish to identify and enumerate all households. A household will be defined as any permanent or semi-permanent structure acting as the primary residence for any family unit. All households identified will be assigned sequential numbers and have their geographic coordinates measured using hand-held global positioning systems (GPS). This information will subsequently be used to generate a detailed digitized map of all households using geographic information system software. The locations of potential mosquito breeding sites, drug shops, pharmacies, clinics, and main roads will also be mapped. Mapping of the study site will be done in collaboration with Jonathan Cox (London School of Hygiene and Tropical Medicine), an expert on mapping using GPS technology. A more complete protocol for our census, mapping, and basic demographics survey of the Mulago III Parish has been prepared separately.

10.2.3 Probability sampling- completed

All households enumerated during the census will be assigned sequential numbers. Using computerized number generation; a random sample of 600 households will be selected to generate a list of households to be screened for subject recruitment.

10.3 Subject recruitment

10.3.1 Initial contact method– Phase I- completed

Experienced home visitors will approach households in the order they were randomly selected and conduct door-to-door screening to identify those households with at least one child aged 1 to 10 years (Figure 1). Households without a child of appropriate age will be removed from the household recruitment list. When a household with at least one child of the correct age range is identified, home visitors will briefly describe the purpose of the study in the appropriate language (usually Luganda) with parent(s) or guardian(s). For those parents/guardians who are interested in the study, the home

visitors will set up an appointment date to bring their children to our study clinic for an evaluation of study eligibility. Residents not home during the initial contact will be re-visited on at least 3 occasions over a 6-week period before eliminating them from our sample selection process. All children between the ages of 1 to 10 years from a single household will be eligible for evaluation for study enrollment.

Figure 1. Subject recruitment

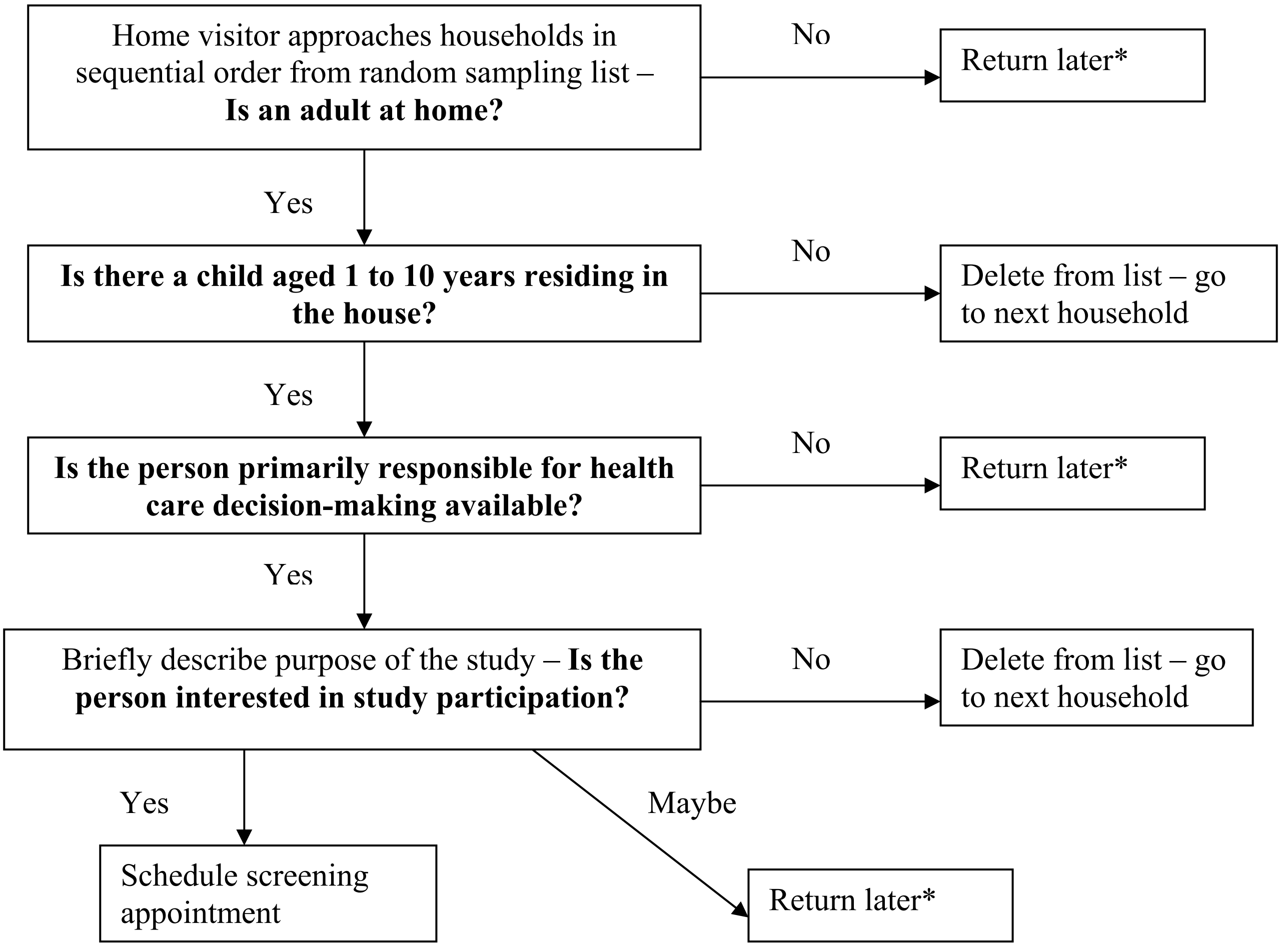

* If unable to make contact with the person primarily responsible for health care decision-making after at least 3 visits or 6 weeks after initial contact, delete from list.

10.3.1.1 Initial contact method – Phase II- completed

Experienced home visitors will approach all households currently in the study beginning in May 2006. Using a standardized script (Appendix F), the home visitors will discuss the addition of insecticide treated bed nets (ITN) to the study design and complete a short questionnaire and observation of current bed net use. Distribution of the ITNs (1 per participant in the household + 1 additional) and a short informative training of the ITN will be completed and the date recorded as a record of when each participant completed Phase I and began Phase II of the study. Lastly, the home visitor will inquire about children between the ages 1 and 10 years, living in the household under the guardian's care, that are not currently enrolled in the study. If such children are identified, the home visitor will set up an appointment date with the guardian to bring these children to the study clinic for evaluation of study eligibility.

10.3.2 Selection criteria – Phase I- completed

Inclusion and exclusion criteria are based on the goal of recruiting a representative sample of children from our target population who can be expected to follow the study protocol and not require an unusually high degree of health care needs due to non-malarial illnesses.

Inclusion criteria

1. Age 1 to 10 years.
2. Agreement to come to the study clinic for any febrile episode or other illness.
3. Agreement to avoid medications administered outside the study.
4. Willingness of parents or guardians to provide informed consent.

Exclusion criteria

1. History (obtained from the parent/guardian) of any known serious chronic disease requiring frequent medical care (e.g. AIDS, sickle cell disease, malignancy).
2. Intention to move from Kampala during the follow-up period.
3. History (obtained from the parent/guardian) of serious side effects to study medications or sulfa drugs.
4. Weight < 10 kg.
5. Severe malnutrition defined as weight-for-height or height-for-age Z-score <-3
6. Homozygous hemoglobin SS (sickle cell) result by hemoglobin electrophoresis
7. Life-threatening screening laboratory value in the absence of malaria
    - Absolute neutrophil count: < 250/mm^3
    - Hemoglobin: < 5.0 g/dL
    - Platelet count: < 25,000/mm^3
    - Creatinine: < 2 years: > 1.5 mg/dL, ≥ 2 years: > 2.0 mg/dL
    - ALT: > 15.0 x ULN
    - Bilirubin: > 7.5 x ULN

10.3.2.1 Selection criteria – Phase II- completed

Inclusion criteria

1. Child and Guardian/Parent belong to household currently enrolled in the study.
2. Age 1 to 10 years.
3. Agreement to come to the study clinic for any febrile episode or other illness.
4. Agreement to avoid medications administered outside the study.
5. Willingness of parents or guardians to provide informed consent.

Exclusion criteria

1. History (obtained from the parent/guardian) of any known serious chronic disease requiring frequent medical care (e.g. AIDS, sickle cell disease, malignancy) including history of seizures.
2. Intention to move from Kampala during the follow-up period.
3. History (obtained from the parent/guardian) of serious side effects to study medications or sulfa drugs.
4. Weight < 10 kg.
5. Severe malnutrition defined as weight-for-height or height-for-age Z-score <-3
6. Homozygous hemoglobin SS (sickle cell) result by hemoglobin electrophoresis
7. Life-threatening screening laboratory value in the absence of malaria
    - Absolute neutrophil count: < 250/mm^3
    - Hemoglobin: < 5.0 g/dL
    - Platelet count: < 25,000/mm^3
    - Creatinine: < 2 years: > 1.5 mg/dL, ≥ 2 years: > 2.0 mg/dL

- ALT: > 15.0 x ULN
- Bilirubin: > 7.5 x ULN

10.3.3 Initial screening criteria- completed

Assessment for eligibility will be done by the study physicians in our study clinic. Interviews will be conducted in the appropriate language with parents or guardians. During the screening process, the study physicians will assess for initial eligibility criteria through conversation with the parent/guardian (including age of the child, willingness of the parent/guardian to participate in the study, avoid medications outside of the study protocol, and to provide informed consent, intention to move from Kampala, any history of known serious chronic illness or serious side effects to study medications or sulfa drugs in the child). If the initial screening criteria are met, the parent/guardian will be asked to provide informed consent for their child to participate in the study. If parents/guardians are undecided about consenting for their child (or children) to participate in the study at the initial screening visit, they will be allowed up to one week to make a final decision about study participation.

10.4 Informed Consent

Study staff will conduct the informed consent discussion in the study clinic with the subject and their parent(s) or guardian(s). Informed consent will be conducted in the appropriate language (usually Luganda) and a translator will be used if necessary. Consent forms will be available in both English and Luganda. Following the informed consent discussion, parents (or guardians) will be asked by the study staff to sign a written consent form approved by the NIH / DMID International Clinical Studies Review Committee, UCSF Committee for Human Research, Makerere University Research and Ethical Committee, and the Ugandan National Council for Science and Technology (UNCST) for their child to participate in a research study, a second approved consent form for the future use of biological specimens obtained during the course of the study (Appendix A), and a third approval for participation in immunology studies (Appendix A2). If the parent or guardian is unable to read or write, their fingerprint will substitute for a signature, and a signature from a witness to the informed consent procedures will be obtained.
In phase II of the study all current and all new study participants will sign a revised consent for participation in a research study. In phase IIa, all participants will sign a revised consent for participation in a research study to explain the change to a single treatment arm (AL) for uncomplicated malaria and extension of follow up through April 2009 (Appendix A).

10.5 Initial Screening/Baseline Evaluation- Phase I- completed

Children who fulfill the initial eligibility criteria and provide informed consent will be assigned a study number in ascending order. Children will undergo a history and physical examination, including measurement of temperature, height, weight, and a standardized assessment that will be used as baseline for monitoring of future potential adverse events. Children who meet our definition of severe malnutrition (weight-for-height or height-for-age Z-score <-3) will be excluded and referred for appropriate management at the Mulago Hospital malnutrition clinic. Children who meet eligibility criteria by history and physical examination will have blood collected by venipuncture (5-10 cc's) for thick blood smear, routine baseline laboratory testing (complete blood count [CBC], creatinine, alanine aminotransferase (ALT), bilirubin, hemoglobin electrophoresis, glucose-6-phosphate dehydrogenase level), and for storage for future research related to malaria and other childhood diseases in Africa (e.g., herpesviruses, such as human herpesvirus 8; hepatitis viruses such as hepatitis B virus; gastrointestinal pathogens; and respiratory pathogens). Children who have history of fever in previous 24 hours or a temperature ≥ 38.0ºC (tympanic) will have their thick blood smear read urgently in the study clinic.

10.5.1 Initial Screening/Baseline Evaluation Phase II-completed

Potential new study participants being evaluated for entry into Phase II of the study will be managed as described for Phase I. In addition, they will be given an ITN and short informative training on its proper use at the end of the initial screening visit/baseline evaluation.

10.6 Final Assessment of Eligibility Criteria and Enrollment-completed

Study follow-up will begin when a subject is documented to be free of symptomatic malaria and when final eligibility criteria based on laboratory testing have been met (Figure 2).

10.6.1 Children without symptomatic malaria at time of initial screening

Children without symptomatic malaria, including those who are afebrile and have no history of fever within the previous 24 hours, and those who have history of fever in previous 24 hours or a temperature $\geq$ 38.0ºC (tympanic) and a negative blood smear, will be asked to return to the study clinic within 72 hours for review of their screening/baseline laboratory tests. If any of the results of the screening laboratory tests are graded as life-threatening (Section 10.3.2), the child will be excluded from the study. In addition, if the child is found to be homozygous for hemoglobin SS (sickle cell disease) by hemoglobin electrophoresis, the child will be excluded. If all laboratory values are within the acceptable range, the child will be enrolled in the study, and study follow-up will begin at that time.

10.6.2 Children with symptomatic malaria at the time of initial screening

Children who are noted to have a history of fever in the previous 24 hours or documented temperature of $\geq$ 38.0˚C (tympanic), and are found to have a positive thick blood smear will be diagnosed with symptomatic malaria and treated with quinine (10 mg/kg TID x 7d). A blood smear will be repeated after 7 days. If the blood smear is negative, and the initial screening/baseline laboratory tests were within the acceptable range, the child will be enrolled in the study and study follow-up will begin at that time. If any of the initial screening/baseline laboratory tests were graded as life-threatening (Section 10.3.2), the tests will be repeated (as the abnormal test results could have been related to the malaria infection), and the child will be asked to return to the study clinic within 72 hours for review of their repeat screening/baseline laboratory tests. If any of the results of the repeat laboratory tests are graded as life-threatening, the child will be excluded from the study. If all laboratory values are within the acceptable range, the child will be enrolled in the study, and study follow-up will begin at that time.

In the unlikely event that a patient fails quinine (with a positive blood smear after 7 days of treatment), therapy will be repeated with quinine (3 days) and clindamycin (5 mg/kg QID for 7 days). A blood smear will again be repeated after 7 days, and the same algorithm for follow-up of laboratory tests will be followed as outlined above. In the very unlikely event that patients remain parasitemic after the two treatments, they will be excluded from the study and referred to the Acute Care Unit in Mulago Hospital, adjacent to our study clinic, for further management.

10.7 Household survey- Phase I- completed

Within approximately a two-week period from the date of enrollment, a household survey will be performed at the participant's household. Using a standardized questionnaire we will interview primary care givers of study participants. Information will be collected on household characteristics, socio-economic demographics, malaria-related knowledge, attitudes, and practices (KAP), and use of preventative measures for malaria. This information will be used for descriptive epidemiological purposes and to prospectively identify predictors of malaria incidence.

10.7.1 Household survey- Phase II- completed

The initial household survey will not be repeated. However, a new survey about household and study subject bednet usage will be completed (Appendix I, P. 94).

10.8 Randomization

Participants will be randomized to their assigned treatment regimen at the time of their first diagnosis of uncomplicated malaria. A computer generated randomization list will be prepared by a member of the project who will not be directly involved in the conduct of the study. The randomized code will correspond to the 3 treatment groups using variable sized blocks of 6 and 9. Sealed copies of the original randomization list and documentation of the procedure used to generate the lists will be stored in the project administrative offices in San Francisco and Kampala. Prior to the onset of the study, a set of sequentially numbered, opaque, sealed envelopes will be prepared. Each envelope will be marked with the treatment allocation number and will contain the treatment group assignment and a carbon paper inside the envelope.

In Phase II of the study newly enrolled children will be randomized to one of two treatment arms, AQ+AS or AL, at the time of their first episode of uncomplicated malaria. All children previously assigned to the AQ+SP treatment arm will be re-randomized to one of the other two treatment arms at the time re-consent is given by the parent/guardian. Beginning on February 1, 2008, AQ+AS will be dropped due to the discontinuation of the AS product. All uncomplicated malaria will be treated with AL.

Figure 2. Subject enrollment

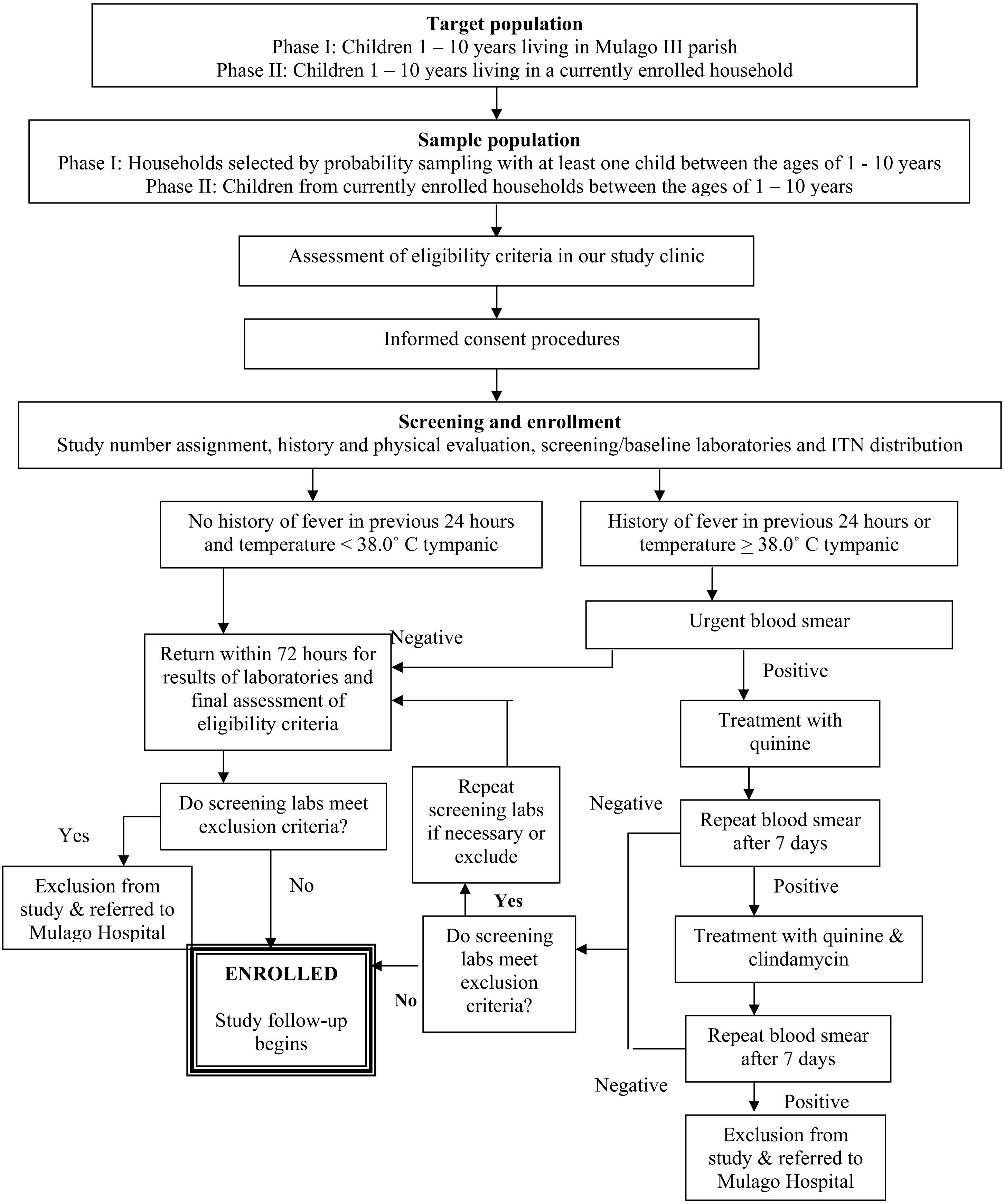

10.9 Treatment allocation

Following the diagnosis of their first episode of uncomplicated malaria, participants will be referred to the study nurse who will be responsible for the treatment group assignment and allocation of the study medications. The study nurse will assign treatment groups as follows:

1. Select next available envelope
2. Note the treatment number on the outside of the envelope
3. Write date, time, and study number on the outside of the envelope
4. Open envelope
5. Remove form containing code for treatment group and date, time, and study number (transferred to form via carbon paper inside of envelope)
6. Store form in file box in study clinic
7. Record onto the treatment allocation master list the study number, enrollment date, treatment assignment code, and study medications to be given.

Subjects will be treated with the same study medications for each episode of uncomplicated falciparum malaria that is diagnosed during the entire follow-up period. The treatment allocation list will link study numbers, treatment numbers, and study regimens. This list will be accessible only to study nurses and senior personnel in Kampala (Dr. Kamya) and San Francisco (Dr. Rosenthal). A copy of the treatment allocation list will be kept in the Acute Care Unit in the event that a patient is diagnosed with uncomplicated malaria outside of study clinic hours (Section 10.10.8). This list will be updated by a study nurse each time a participant is diagnosed with their first episode of uncomplicated malaria.

Phase II of the study will be conducted as an open-label trial and treatment allocation will no longer be blinded to members of the study team or participants.

Beginning in February 2008 (Phase IIa), the AQ+AS treatment arm will be dropped due to the discontinuation of the AS product. Since all uncomplicated malaria will be treated with AL, the treatment allocation envelopes and procedures will be discontinued.

10.10 Subject follow-up

The study clinic will be open at Mulago Hospital 7 days a week from 8 a.m. to 5 p.m. Parents/guardians will be asked to bring their child to the study clinic for all medical care. (See Figure 4 for outline of Subject Follow Up.) Visits will be classified into the following four categories:

1. **Initial visit**: Any visit to the study clinic for a new medical problem.
2. **Malaria follow-up visit**: Any scheduled or unscheduled visit at the study clinic, following the diagnosis of malaria, during the standardized 14-day follow-up period.
3. **Non-malaria follow-up visit**: Any scheduled or unscheduled visit at the study clinic, for a previously diagnosed non-malaria illness.
4. **Routine visit (Phase I)**: Conducted at the study clinic or the participant's home, to assure protocol compliance and obtain blood smear for participants not seen for malaria in a consecutive 30-day period of time.
5. **Routine visit (Phase II):** Conducted at the study clinic or the participant's home, to assure; 1) protocol compliance for participants not seen for malaria in a consecutive 30-day period of time or 2) to collect laboratory specimens in the study clinic if not seen for malaria in a consecutive 90-day period of time.

10.10.1 Initial visit

At each initial visit, subjects who are febrile (tympanic temperature ≥ 38.0˚C) or report history of fever in the past 24 hours will have blood obtained by fingerprick for a thick blood smear. If the thick blood smear is positive, the patient will be diagnosed with malaria. If the thick blood smear is negative, the patient will be managed by study physicians for a non-malarial febrile illness (Section 10.10.7). If the patient is afebrile and does not report a recent fever, a thick blood smear will not be obtained, except for routine purposes (Section 10.10.9). When appropriate, patients will be managed according to treatment algorithms designed to provide standardized guidelines for the treatment of common non-malarial illnesses.

10.10.2 Diagnosis of malaria

All episodes of malaria will be classified as uncomplicated or complicated based on the following criteria:

Uncomplicated malaria (all of the following)

1. Fever (≥ 38.0ºC tympanic) or history of fever in the previous 24 hours
2. Positive thick blood smear
3. Absence of complicated malaria

Complicated malaria (any of the following in the presence of parasitemia)

1. Evidence of severe disease (per WHO definitions, Appendix C.2) [36]
2. Danger signs present (inability to stand or drink, lethargy, recent convulsions, and persistent vomiting)[37]
3. Parasite density ≥ 500,000/ul

10.10.3 Management of uncomplicated malaria

10.10.3.1 Baseline evaluation

On Day 0 of each malarial episode, patients will undergo a standardized clinical history and physical examination and have blood samples obtained by phlebotomy (2-5 cc blood) for thin blood smears, CBC (leukocyte count, differential, hemoglobin, platelet count), ALT measurement, and for storage for future research related to malaria and other childhood diseases in Africa. Any patient who is suspected of having severe anemia on Day 0, based on clinical findings, will have a hemoglobin level measured urgently in the study clinic using a portable spectrophotometer (Hemocue, Anglom, Sweden).

10.10.3.2 Administration of study medications

Patients will be treated with the appropriate study medications based on their assigned treatment regimen (Sections 10.8 and 10.9)a; with initiation of Phase IIa, all uncomplicated malaria will be treated with AL. Medications will be prepared and dosed by study nurses according to weight-based guidelines for administration of fractions of tablets (Appendix B). Study medications administered in study clinic to young children will be crushed, and mixed with water and sugar. Study medications administered in study clinic to older children will be given as tablets or fractions of tablets to take orally. The study nurse will directly observe consumption of study medications in study clinic. Any patient who vomits the medication within 30 minutes of administration will be retreated with a second dose. Any patient who vomits repeatedly (> 3 times) will be treated with parenteral quinine and recoded as having complicated malaria. Study medications to be administered at home will be packaged in opaque envelopes with dosing instructions written on the inside. Parents/guardians will be instructed to bring their child to the study clinic (if prior to 5 pm) or the Acute Care Unit (if after 5 pm, see Section 10.10.8) in the event that their child vomits study medication administered at home. All study drug treatments given in study clinic and for dosing at home will be recorded in a treatment administration book accessible only by assigned and authorized study nurses.

10.10.3.3 Additional treatment

Patients will receive acetaminophen (10mg/kg) for use every 6 hours until the resolution of fever. Patients found to have uncomplicated malaria and a concomitant illness will be treated for both and followed up according to the study protocol. For patients with anemia (Hb < 10 g/dL), we will follow Integrated Management of Childhood Illness (IMCI) guidelines: anemic children will be treated with iron sulfate (100 mg po qD for 2 weeks) and mebendazole (only children > 1 year of age; 250 mg age 1-2 years; 500 mg > 2 years age; treated no more frequently than every 6 months).

10.10.3.4 Follow-up for uncomplicated malaria

For each episode of malaria, patients will be evaluated clinically on Days 1, 2, 3, 7, 14, and on any unscheduled day that they feel ill (Table 1). Patients who do not return for a scheduled visit will be visited at home and, if necessary, transported to the study clinic. Blood will be obtained by fingerprick on Days 2, 3 and 7 for thick blood smears (for parasite density and gametocytes) and filter paper collection. Blood will be obtained by fingerprick on any unscheduled day, only when a fever is documented or reported in the previous 24 hours, for thick blood smears (for parasite density and gametocytes) and filter paper collection. Blood will be obtained by phlebotomy (2-5 cc blood) on Day 14 for thick blood smears, CBC (leukocyte count, differential, hemoglobin, platelet count), ALT measurement, and for storage for future research related to malaria and other childhood diseases in Africa. Malaria diagnosed more than 14 days after a previous episode will be considered a new episode in accordance with standard local practice. If a patient is not diagnosed with a new episode of uncomplicated malaria within 2 weeks of an adequate clinical response, they will have a finger prick done at home on follow-up day 28 for thick blood smear (for parasite density and gametocytes) and filter paper collection. The result of this blood smear will be used for classifying 28-day parasitological outcomes and will not impact on patient management.

Table 1. Follow-up schedule for uncomplicated malaria episodes

	Day 0	Day 1	Day 2	Day 3	Day 7	Day 14	Day 28*	Unscheduled
History	X	X	X	X	X	X		X
Physical exam	X	X	X	X	X	X		X
Temperature measurement	X	X	X	X	X	X		X
Case record form	X	X	X	X	X	X		X
Thick blood smear	X	X[‡]	X	X	X	X	X	X[†]
Thin blood smear	X							
Filter paper sample	X		X	X	X	X	X	X[†]
Blood sample storage	X					X		
Complete blood count, ALT	X					X		

* Visit done at home if new episode of uncomplicated malaria not diagnosed between days 15-28
‡ Done only in cases where complicated malaria develops
† Thick smear and filter paper sample obtained only if temperature elevated (≥ 38.0˚C tympanic) or history of fever in previous 24 hours

10.10.3.5 Visit windows for follow-up of uncomplicated malaria

If a patient does not come to their visit on Days 1 or 2, it will be considered a missed visit. If a patient does not come to their visit on Day 3, we will allow for 24 hours before this will be considered a missed visit. For all other visits (Days 7, 14, and 28) we will allow up to 48 hours to find the patient before it is considered a missed visit.

10.10.4 Management of complicated malaria

Any patient who is diagnosed with severe malaria, danger signs, or hyperparasitemia (defined as > 500,000 asexual parasites / µl), either on an initial visit or during follow-up, will be referred to Mulago Hospital for consideration for admission and treatment with parenteral quinine. Patients not admitted to the Mulago Hospital will receive oral quinine to complete a 7-day course of therapy and will continue follow-up at the study clinic. Hospitalized patients will be seen daily by study personnel including scheduled study follow-up procedures. After hospital discharge, follow-up will involve the same schedule as that described for those with uncomplicated malaria.

10.10.5 Outcome classification system for patient management

Short-term (14-day) treatment outcomes not adjusted for genotyping will be measured using the new standard WHO classification system (early treatment failure, late clinical failure, late parasitological failure, and adequate clinical and parasitological response) for the purposes of patient management (Appendix C). Outcomes will be classified into the following categories for the purposes of patient management:

1) Early or late clinical failure → treatment with quinine (Section 10.10.6).
2) Adequate clinical and parasitological response or late parasitological failure → no additional treatment
3) "Excluded during follow-up" → treatment outcome not classified. Criteria and patient management below.
    i. use of antimalarials outside of the study protocol – treatment with quinine if criteria for early or late clinical failure met.
    ii. lost to follow-up (defined as unable to locate patient on Days 1-3 or unable to locate patient within 48 hours of Day 7 or 14) - treatment with quinine if criteria for early or late clinical failure met.
    iii. withdrawal of informed consent – no further follow-up.

Longer-term treatment outcomes (after day 14) will also be assessed after adjustment by genotyping; however this information will not be used for patient management (Section 14.2.1)

10.10.6 Management of clinical treatment failures

Patients treated for uncomplicated malaria that are classified as early or late clinical failures (within 14 days of treatment) will be treated with quinine (10 mg/kg TID x 7d). The first day of quinine therapy will be considered Day 0 for a new 14-day follow-up period. In the unlikely event that a patient fails to have an adequate clinical response to quinine (either as first-line therapy for complicated disease or second-line therapy), treatment will be repeated with quinine (3 days) and clindamycin (5 mg/kg QID for 7 days), again restarting the standard 14-day follow-up protocol. Only the first dose of medication on Day 0-3 will be directly observed. In our experience, failure to respond to quinine therapy is generally an indication of non-compliance. In the very unlikely event that a patient fails to have an adequate clinical response to quinine plus clindamycin therapy, they will be withdrawn from the study and referred to Mulago Hospital for further evaluation.

10.10.7 Management of non-malaria illnesses

Patients who are found to have illnesses other than malaria will receive standard-of-care treatment in the study clinic, according to standardized algorithms, or will be referred to the appropriate facility at the Mulago Hospital complex. We will avoid the routine use of non-study medications with antimalarial activity, including tetracycline, antifolate, and macrolide antibiotics, when acceptable alternatives are available. During follow-up for non-malarial illnesses, blood smears will be done at the discretion of the study physician if the subjects are febrile (tympanic temperature $\geq$ 38.0˚C) or report history of fever in the past 24 hours. If the blood smear is positive, the patient will be diagnosed with a new episode of malaria and managed per study protocol. If a patient comes to the study clinic for a non-malarial illness and 30 days have passed since the last blood smear, a Routine Visit will be completed in the study clinic (Section 10.10.9). If a patient is diagnosed with a non-malarial illness at the same time as malaria or during malaria follow-up, treatment will be at the discretion of the physician, but this will have no impact on the management of malaria.

10.10.8 After hours visits

Study participants will be encouraged to visit the Mulago Hospital pediatric emergency department (Acute Care Unit; ACU), which is adjacent to the study clinic, when urgent care is needed outside of study clinic hours (Figure 3). Participants will be instructed to inform ACU personnel of their involvement in the study at the time of registration and to visit the study clinic on the following day.

Identified ACU personnel will be educated about the study protocol and a nurse, employed by the project, will oversee adherence to the study protocol.

If a patient is diagnosed with uncomplicated malaria and has already been assigned a treatment regimen or has vomited a dose of study medication at home, he/she will receive treatment from an ACU supply of study medications based on a treatment allocation list maintained in the ACU. If a patient is diagnosed with severe malaria, he/she will receive quinine following standard treatment guidelines of both Mulago Hospital and this protocol. Patients with non-malarial illnesses will be managed at the discretion of the ACU staff. Upon discharge from the ACU, patients will receive follow-up at the study clinic as outlined above. Study personnel will visit the ACU daily to inquire about visits from study subjects and facilitate follow-up in the study clinic.

Figure 3. Management of study participants in the Acute Care Unit

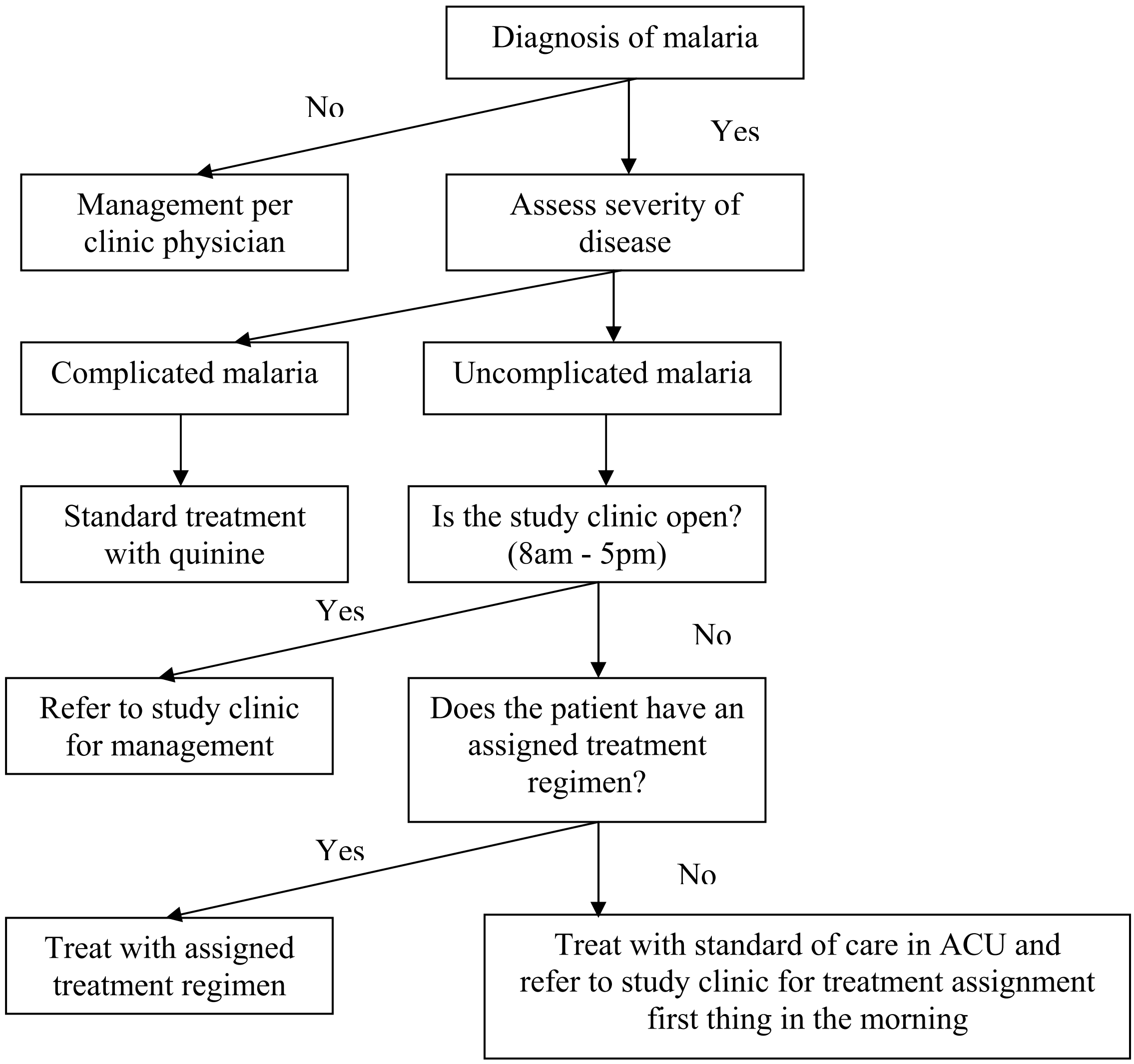

10.10.9 Routine visits- Phase I- completed

Routine evaluations to be done in afebrile patients will include collection of blood for thick blood smear, CBC and ALT measurement, and for storage for future research related to malaria and other childhood diseases in Africa. Subjects who have not had a blood smear done for any consecutive 30-day period will be visited at home for routine assessment. Routine assessment may also be done in the study clinic when the patient presents with a non-febrile illness. Subjects will be asked about visits to outside health facilities and the use of any medications outside the study protocol. The study protocol

will be reinforced with discussion regarding the need to come to the study clinic promptly with the onset of any illness and to avoid use of outside medications. A routine history and physical will be performed using a standardized clinical assessment form. If a patient reports a fever in the last 24 hours or has a documented temperature of > 38.0˚C tympanic, they will be brought promptly to our study clinic for a full assessment. For participants without fever, blood will be collected by finger prick for thick smear and filter paper samples to assess for asymptomatic parasitemia. Routine smears will be read within 48 hours, and asymptomatic children with parasitemia ≥100,000/μl will be considered to have new episodes of malaria, brought to the study clinic within 24 hours, and treated with their assigned treatment. In our experience, asymptomatic patients with a parasite density ≥ 100,000/μl have a high risk of developing symptomatic malaria in the next few days (Njama, unpublished data). Asymptomatic patients with a parasite density of < 100,000/μl will not be treated in accordance with standard practices in endemic areas of Africa. If laboratory tests have not been performed during the previous 3 months, patients will be brought to the study clinic for routine assessment (in place of a monthly routine visit at home) where blood will be obtained by phlebotomy (instead of by fingerprick) for thick blood smears, CBC and ALT measurement, and storage for future research related to malaria and other childhood diseases in Africa.

10.10.9.1 Routine visits- Phase II

Routine visits will be the same for Phase II as for Phase I, except that, for Phase II, subjects will be visited at home if they have not been seen in the clinic or at home for any 30-day period, but they will not be subject to finger-pricks on these home visits. Routine evaluations at the clinic, performed when subjects have not been evaluated in the clinic for any 90-day period, will be conducted as described for Phase I.

10.10.9.2 Visit windows for routine visits- Phase I- completed

If, upon any visit to the study clinic, a participant last had a thick blood smear done 24 or more days previously, we will collect a thick blood smear and filter paper sample for routine purposes. If a participant has not been seen at the study clinic for 28 days after a prior visit, we will begin to actively look for them at home for routine assessment (section 10.10.9).

For routine 3-monthly visits for clinic assessment and laboratory testing (CBC/ALT), if upon any visit to the study clinic, a participant last had CBC/ALT performed 84 days or more day previously, we will collect blood for CBC/ALT for routine purposes. If a patient has not been seen at the study clinic for 88 days after a prior visit, we will begin to actively look for them at home for routine evaluation and CBC/ALT.

There will be no specific endpoint for visit windows for routine visits. If we are unable to locate a study participant at home, and have no contact with them for > 60 days, they will be withdrawn from the study as described in section 10.11.

10.10.9.3 Visit windows for routine visits- Phase II

The management plan will be as described for Phase I, except that home finger sticks will not be performed. Clinic phlebotomy will be performed as described for Phase I.

10.10.10 Medical care outside of the study clinic

We will provide all routine medical care, including evaluations, medications available in our clinic, and cost of any transportation free of charge. In addition, we will reimburse the cost of consultation for referrals made by study physicians to other clinics and services within the Mulago Hospital Complex, and visits to the Acute Care Unit. We anticipate reimbursing the cost of most diagnostic tests (including laboratory test, X-rays, and ultrasounds) and medications resulting from these referrals, using available funds. However, reimbursement of all diagnostic tests and treatment

recommended outside the study clinic cannot be guaranteed in all circumstances because neither the University of California, San Francisco nor the U.S. Federal Government have a program to cover these costs. Decisions on reimbursement will be made by the study coordinator and the investigators, in conjunction with the funding agency if necessary.

Figure 4. Subject follow-up

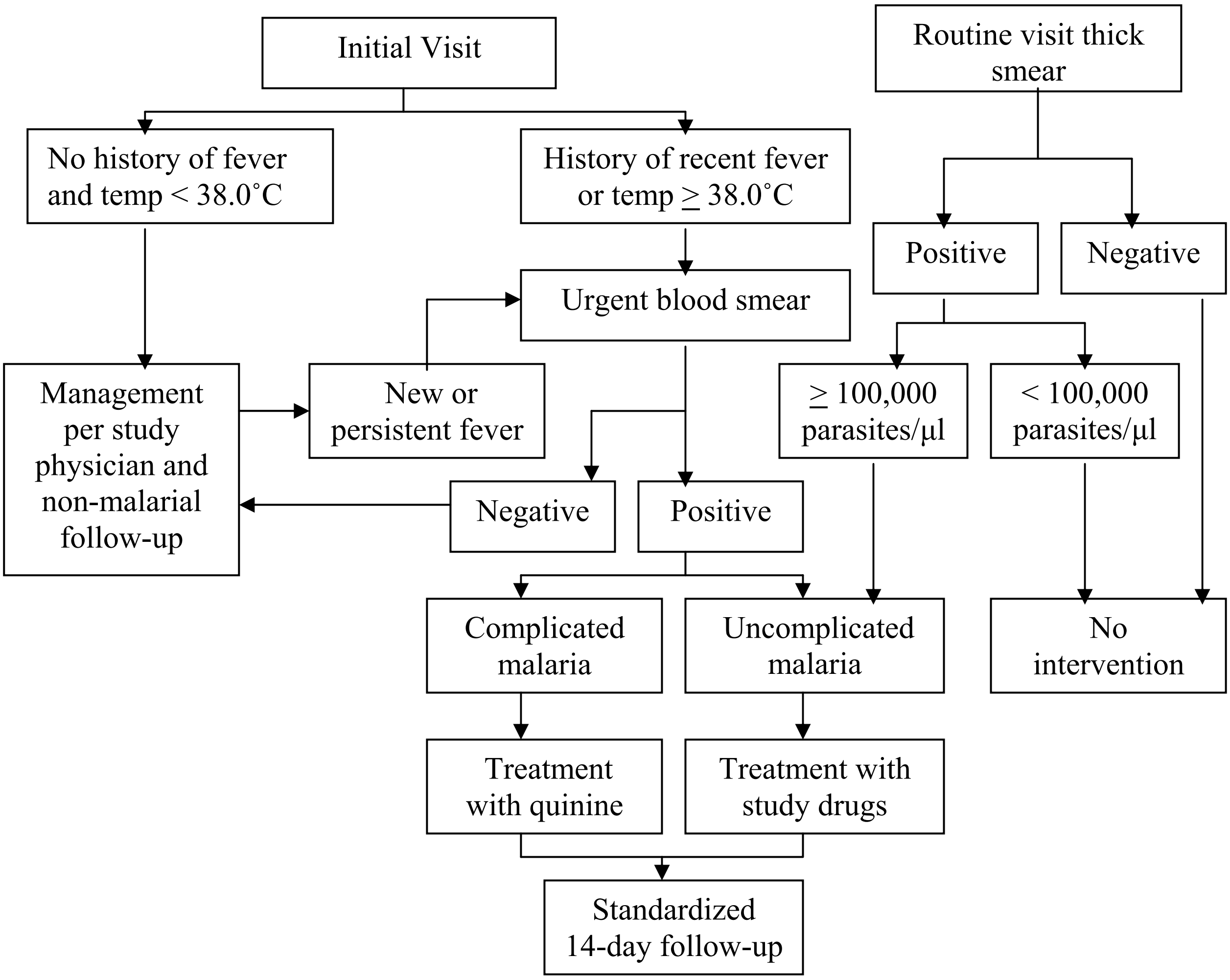

10.11 Subject withdrawal criteria

Enrolled subjects will be withdrawn from the study for the following reasons:

1. Movement out of Kampala for > 60 consecutive days
2. Inability to be located for > 60 consecutive days
3. Withdrawal of informed consent
4. Development of any serious adverse event felt to be probably or definitely related to study medications
5. Failure to achieve an adequate clinical response after treatment with quinine plus clindamycin
6. Unable to comply with the study schedule and procedures
7. Diagnosis of a serious chronic disease requiring frequent medical care.

If a subject is withdrawn for reasons # 1, 2, or 3, we will be unable to perform any additional study procedures, and will not plan to obtain any follow-up tests. If a subject is withdrawn for reasons # 4, 5 or 7, because a serious health problem developed (including a serious adverse event which is probably or definitely related to the study medication, failure to achieve clinical cure following three rounds of antimalarial treatment, or diagnosis of a serious chronic medical illness), plans to obtain

appropriate follow-up tests will be individualized to each subject. If the subject is withdrawn for reason # 6, plans to obtain appropriate follow-up tests, will be individualized to each subject, depending on the health status of the subject at the time of withdrawal, and the willingness of the participant and their parent/guardian to proceed with additional testing.

11.0 STUDY INTERVENTIONS

11.1 Study medication formulation and labeling

Table 2. Individual Study Medications (for Phase IIa, only AL will be used)

**Drug**	**Trade name Manufacturer**	**Class**
Amodiaquine (200mg)	Camoquin<br>Pfizer (formerly Parke-Davis)	4-aminoquinoline
Sulfadoxine-pyrimethamine (500mg/25mg)	Fansidar<br>Roche	Antifolate combination
Artesunate (50mg)	Arsumax<br>Sanofi-Synthelabo	Artemisinin derivative
Coartemether (AL)<br>(20 mg artemether /120 mg lumefantrine)	Coartem<br>Novartis	Fixed combination artemisinin derivative and synthetic fluorene compound

11.2 Study supply acquisition

All study medications will be obtained prior to the onset of the trial. AQ, SP, AS, and coartemether will be ordered through SurgiPharm pharmacy in Kampala, Uganda.

11.3 Storage

All agents will be stored in their original packaging in the study clinic prior to preparation for administration.

11.4 Investigational drug accountability

A registry of all study medication current product labels and Certificates of Analysis, provided by SurgiPharm, will be maintained within the regulatory binder for the study. The date received, lot number, expiration date, and date used will be recorded for each of the study medications. Monthly inventory of all study medications will be conducted and a record log of investigational medications kept at the study clinic.

11.5 Blinding- Phase I - completed

The subjects, their caregivers, physicians, and laboratory technicians will be blinded to the treatment groups. Study nurses, who will be responsible for distributing study medications, will not be blinded, and will not be involved in outcome assessment or adverse event monitoring.

11.5.1 Blinding- Phase II

Study investigators will be unblinded for Phase II for purposes of analysis of Phase I safety and efficacy data. The study will therefore be an open-label trial during Phase II.

11.6 Administration of study medications

All treatment regimens will follow the same dosing schedule consisting of twice daily dosing for 3 days (Appendix B). Study nurses will prepare the study medications. Administration of all morning

doses will be directly observed in the study clinic. For young children, study medications will be crushed, and mixed with water and sugar. Older children will be given tablets or fractions of tablets to take orally. Following morning administration of study medications, a second dose of medication or placebo will be given with instructions for administration at home in the evening.

11.7 Other medications

Other medications will be used in a non-blinded fashion for the management of specific problems as detailed in Section 10 and Table 3. Quinine is the standard treatment in Uganda for malaria after the failure of first-line agents and the combination of quinine and clindamycin is a standard pediatric therapy for resistant malaria in many parts of the world, including the United States. Acetaminophen is a standard, well-tolerated antipyretic, and the combination of mebendazole and iron sulfate for anemia is standard management in Uganda, based on Integrated Management of Childhood Illnesses (IMCI) guidelines.

Table 3. Other medications

Drug	Indication	Dosage
Quinine	Malaria (after treatment failure)	10 mg/kg TID x 7d
Clindamycin	Malaria (with quinine after failure of quinine treatment)	5 mg/kg QID for 7 days
Acetaminophen	Fever (after malaria diagnosis)	10mg/kg every 6 hours
Mebendazole	Anemia (Hb < 10 g/dL)	Only children > 6 mo. age; 250 mg age 1-2 years; 500 mg age > 2 years; treated no more frequently than every 6 months
Iron sulfate	Anemia (Hb < 10 g/dL)	100 mg po qD for 2 weeks

11.8 Contraindicated drugs

We will avoid use of drugs that are metabolized by cytochrome CYP2D6 and are commonly used in Uganda, including amitriptyline, codeine, dextromethorphan, haloperidol, metoprolol, and tramadol, given the possibility of interaction with artemether + lumefantrine. In addition, we will avoid use of drugs that prolong the QTc interval and are commonly used in Uganda, including erythromycin, clarithromycin, azithromycin, fluoroquinolones, cisapride, astemizole, and terfenadine. Participants requiring prolonged or chronic therapy with these drugs will be terminated from study participation.

11.9 Insecticide treated bed nets (ITN)

Each study participant of the study will be given a new long-lasting insecticide treated mosquito net (PermaNet®) with written instructions for proper use. In addition, each household will be given one additional bed net. PermaNets (Vestergaard Frandsen A/S Denmark) are high quality nets made of 100% polyester and pre-treated with 55 mg/m^2 deltamethrin insecticide. These nets can be washed up to 20 times before the need for retreatment. The nets will remain effective against mosquitoes for up to 4 years when washed every three months following washing instructions. Nets washed more frequently will need to be retreated after the 20th wash.

12.0 DIAGNOSTICS

12.1 Microscopy

Thick and thin blood smears will be stained with 2% Giemsa for 30 minutes and read by experienced laboratory technologists who are not involved in direct patient care. Parasite densities will be calculated by counting the number of asexual parasites per 200 leukocytes (or per 500 leukocytes, if the count is <10 asexual parasites/200 leukocytes), assuming a leukocyte count of 8,000/µl. A blood smear will be considered negative when the examination of 100 high power fields does not reveal asexual parasites. Gametocytemia will also be determined from thick smears. Thin smears will be used for parasite species identification. Urgent thick smears will be read in our study clinic for initial diagnosis and to identify treatment failures during follow-up. Routine blood smears will be read within 48 hours. For quality control, all slides will be read by a second microscopist and a third reviewer will settle any discrepant readings.

12.2 Clinical laboratory studies

At certain time points, as noted in Section 10, venipuncture blood samples will be used for clinical laboratory studies. Additional venipunctures will be performed, as appropriate, for follow-up of adverse events. Laboratory studies will be performed at the MUWRP lab, a facility that meets good laboratory practice (GLP) guidelines and is located on the Mulago Hospital campus.

12.3 Molecular studies

Each time a thick blood smear is obtained blood will also be collected onto filter paper. Samples will be collected by venipuncture or by fingerprick sampling. Blood will be placed onto filter paper in approximately 25 ul aliquots per blood spot (4 blood spots per sample). The samples will be labeled with study numbers and dates, air-dried, and stored in small, sealed sample bags at ambient temperature with desiccant. Molecular studies will involve the extraction of DNA from filter paper and characterization of polymorphisms using standard molecular procedures including PCR, DNA hybridization, and/or restriction enzyme digestion. For all repeat episodes of malaria, molecular genotyping methods, similar to those described for polymorphism analysis, will be used to distinguish recrudescent from new infections. Additional molecular studies will include analyses of polymorphisms in parasite and/or human genes for mutations that may impact on clinical malaria. Molecular studies will be performed only for research purposes and will have no impact on the clinical management of study patients.

12.4 Future laboratory research

As noted in section 10, phlebotomy will be performed at enrollment, on Days 0 and 14 for each episode of malaria, and every 3 months, if malaria has not been diagnosed over the last 3 month period. These blood samples will be used to collect and store serum and plasma and, for selected patients, for the preparation and storage of blood cells. These samples will be available for the evaluation of infectious diseases by serological methods, the identification of pathogens by molecular or microbiological methods, and the assessment of immune responses to infection. Consent for these studies is covered by the Informed Consent for Future Use of Biological Specimens.

12.5 Parasitology and immunology studies – Phase I

To improve our understanding of antimalarial drug resistance, we will evaluate parasites that cause malaria in the laboratory in a sub-set of patients. For selected patients diagnosed with uncomplicated malaria, when blood is drawn for routine evaluations as specified in this protocol, approximately 1 ml of blood will be transferred to our molecular laboratory at Mulago Hospital for parasite culture. Parasitology studies will not require additional phlebotomy from study subjects. Parasites will be cultured following standard protocols and evaluated for in vitro drug sensitivity, molecular characteristics, and other features. Information from the parasitology studies will have no impact on patient care.

In order to improve our understanding of the immune response to malaria, we will be adding laboratory studies of the T-cell response to malaria-specific antigens. These studies will be done on fresh whole blood samples at the Joint Clinical Research Centre CTL laboratory. The results of the studies will be used for research purposes only. Effective following approval of protocol version 1.7, each time phlebotomy is performed on a study participant (as outlined in section 10) an additional 2-5 ml will be collected for immunology studies in a separate tube. These studies will not require any additional blood draws. Given the need to increase the amount of blood taken from 5 mls to 7-10 mls, parents/guardians will asked to provide informed consent for these additional laboratory studies. Additional blood for immunology studies will not be taken from children of parents/guardians who decline to provide informed consent. There will be no consequences for children of parents/guardians who decline to provide informed consent. In the event that study physicians are unable to obtain 10 mls of blood during phlebotomy, immunology studies will not be performed for that blood draw.

Flow cytometry-based assays will be performed on freshly isolated peripheral blood mononuclear cells (PBMC). Intracellular cytokine release in response to synthetic peptides corresponding to relevant antigens will be used to evaluate the frequency and phenotype of T cell responses. Briefly, PBMC will be incubated overnight with peptides at 37° C in 5% CO_2 in the presence of co-stimulatory anti-CD49d and CD28 (1μg/ml, Becton-Dickinson, San Jose, CA). Brefeldin A (10 μg/ml) will be added and cells will be fixed and permeabilized according to the Becton Dickinson protocol and stained with fluorochrome monoclonal antibodies (Mab): IFN-g, CD107 or IL-2 and CD3, CD4, and CD8 (BD Pharmingen, San Diego, CA). Responses will be evaluated and correlated with clinical status. Analysis will be performed using the FACSCalibur Flow Cytometer (BD Biosciences, Mountain View, CA) and FlowJo software 4.0 (Tree Star, Inc, San Carlos, CA). Negative and positive controls will be unstimulated (no peptide) and stimulated (PHA-1 μg/ml, Murex Biotech) cells, respectively. Results will be expressed as Net Percent cytokine positive T cells (Net percent =% peptide-specific - % control). Both CD4+ and CD8+ T cell responses will be evaluated with the goal to further understand the potential mechanism of immune protection in children infected with malaria, which will ultimately be an important tool for vaccine development.

12.5.1 Parasitology and immunology studies – Phase IIa
Host immunity plays an important role in the outcomes of malaria infections, but biomarkers of parasite exposure and clinically relevant immunity are poorly characterized. Antibodies to malaria antigens represent potential biomarkers which may fulfill this void. We will evaluate serum and plasma samples already being collected as part of this protocol for antibodies to *Plasmodium falciparum* antigens. Since serum and plasma samples are already being collected and stored, antibody studies will not require any additional phlebotomy and will not require additional consent. Antibodies to *P.falciparum* antigens, including MSP-1, MSP-3, CSP, LSA-1, GLURP, schizont extract, and potentially others identified as important in antimalarial immunity will be measured using standard ELISA tests. Levels of antibodies will then be related to clinical outcomes observed during the trial, such as malaria incidence and outcomes of antmalarial therapy. Discovery of associations between antibody response to *P. falciparum* antigens and clinical outcomes can then be used to account for parasite exposure and the contribution of host immunity to clinical outcomes in futures studies. Tests for these studies will be carried out in part at both the UCSF Rosenthal Lab and the laboratory of Dr. Chandy John at the University of Minnesota. Studies of host immunity will only be performed on participants who have signed our consent form approving future use of their biological specimens.

In addition, serum and plasma samples already stored as described in this protocol for immunology studies will be used to: 1) determine the prevalence of antibodies to *Helidobacter pylori* across different age strata; 2) determine how antibiotic use in these children may affect *H.pylori* serostatus; and 3) examine the relationship between *H.pylori* serostatus and malaria incidence. Samples will be shipped to New York University where *H.pylori* antibody testing will be conducted under the auspices of Dr. Martin Blaser. No patient identifiers will be shared with New York University. Data analysis,

including studies of associations between antibody levels and clinical measures will be conducted by the investigators of this protocol.

13.0 DATA COLLECTION AND MANAGEMENT

13.1 Data management

All clinical data will be recorded onto standardized case record forms by study physicians. Laboratory data will be recorded in a laboratory record book by the study laboratory technologists and then transferred to the case record forms by study coordinators, who will review the case record forms frequently for completeness and accuracy. Data will be transferred from the case record forms into a computerized database and will be double entered to verify accuracy of entry. Adverse event data will be transferred onto standardized data extraction forms prior to entry into the database. Back-up files of the database will be stored on zip or compact discs after each data entry session. For quality control, query programs will be written into the database to limit the entry of incorrect data and ensure entry of data into required fields.

13.2 Data quality assurance and monitoring

All members of the study team will be educated in the study protocol prior to the onset of the trial. Knowledge of the study protocol and procedures will be assessed and documented with a post-training questionnaire. The study physicians will complete case record forms at each patient visit. These forms will be reviewed by the study coordinator for completeness and accuracy. To optimize the quality of thick blood smear slide readings, each slide will be read by two expert microscopists who will be blinded to the patient's treatment group. Any discrepancies in slide readings will be reviewed and resolved by a third microscopist. Study group meetings will be conducted regularly to review the progress of the study, address any difficulties, and provide performance feedback to the members of the study group.

13.3 Record keeping

Case record forms will be provided for each subject. Participants will be identified by their study identification number on study documents and patient names will not be entered into the computerized database. All patient record forms will be kept in individual files in a secured filing cabinet in the study clinic. All corrections will be made on case record forms following GCP guidelines by striking through the incorrect entry with a single line and entering the correct information adjacent to it. All corrections will be initialed and dated by the investigator making the correction. Additional records will be kept in the clinical and laboratory record books, which will be stored in the central study laboratory in Makerere University Medical School. The investigators will cooperate with all requested monitoring visits, audits, or IRB or DSMB reviews.

14.0 STATISTICAL CONSIDERATIONS

14.1 Primary outcome

The effect of antimalarial drug therapy can be measured both in terms of drug efficacy (risk of true treatment failure) and post-treatment prophylactic effect (risk of new infection). To best reflect the overall impact of therapy, our primary outcome measurement will be the treatment incidence density (treatments per time at risk) for each treatment arm. To eliminate the period not influenced by study drugs, treatment count will exclude the first episode. Follow-up time will be from the first episode to the end of the study. Treatment count will include both first-line treatments with study drugs and second-line treatments with quinine following study drug failure. It will be assumed that participants will not be at risk for repeat therapy for 14 days after treatment with quinine, for which resistance has not been reported, so this time will be excluded when calculating total time at risk.

14.2 Secondary outcomes

14.2.1 Drug efficacy

We will examine the efficacy of the different treatment groups using each episode of malaria treated with a study drug as the unit of analysis. We will examine the risk for repeat treatment as a function of time. Short-term (14-day) assessments of treatment efficacy will provide a standard analysis that will be useful for comparisons with other studies. Specific short-term outcomes to be assessed will include (1) clinical and parasitological outcome, (2) rates of fever and parasite clearance, (3) change in hemoglobin level from day 0 to 14, (4) presence of gametocytes following treatment, and (5) safety and tolerability of study medications. Long-term (beyond 14-day) outcomes will be (6) risk of recrudescence and (7) risk of reinfection using Kaplan-Meier product limit estimates of risk at various time intervals (i.e. 4, 6, and 8 weeks after initiation of therapy). In the analysis of long-term outcomes, molecular genotyping will be used to distinguish recrudescence (true treatment failure) from new infections.

14.2.2 Safety and tolerability

All adverse events will be catalogued based on their frequency, severity, and relationship to study medication using standardized protocols (Section 15). These indices of safety and tolerability among treatment groups will be compared using each episode of malaria treated with a study drug as the unit of analysis.

14.2.3 Other long-term outcomes

Other long-term outcomes that will be assessed will include (1) incidence of asymptomatic parasitemia, (2) change in hemoglobin level over time, (3) perceived tolerability of study medications among subjects and care givers, and (4) drug costs (comparison of total cost per patient).

14.3 Sample size calculations

We will enroll 600 persons (approximately 200 in each treatment group). Data from an earlier study in the same area of Kampala indicate that approximately 75% of subjects will have at least one episode of malaria (about 150 per treatment arm), and those with malaria will average a total of 2.75 treatment episodes per person year. Assuming 10% attrition per year, this will result in approximately 1120 treatments per arm over 3 years. To approximate the statistical power that can be expected for the analyses of the primary outcome described above, we employ normal approximations for the outcome defined as log{(1+treatments)/follow-up}, where we have added 1 to the treatment count (or, equivalently, included the initial treatment in the count) in order to allow taking the logarithm. Our preliminary data show that this outcome measure is approximately normally distributed and has a standard deviation of 0.21. By standard calculations for normally distributed data, this implies that 150 per group will provide 80% power to detect a difference between two treatment arms of 0.068 (log base 10) at p=0.05, two-sided. This corresponds to a 17% difference in treatment incidence density between the arms.

Calculations for secondary outcomes of treatment efficacy will be based on the estimated risk of recrudescence after correction by genotyping. The unit of analysis will be each treatment for malaria. Therefore, the effective sample size for efficacy will depend on within-person correlation in outcomes and will be at least 150 per arm (the number of subjects) and at most 1120 per arm (the number of episodes). At this time the optimal duration of follow-up to detect significant differences among the treatment arms is unknown. However, we predict that this difference will be apparent after 42 days based on pharmacokinetic factors and published reports of time to recrudescence [3]. Based on data from our pilot study, we expect approximately 6% of patients treated with AQ+SP (our current "best available treatment") to develop recrudescence with resistant parasites within 42 days of follow-up. We are interested in whether any of the other combination therapy groups will have a risk of treatment failure of 15% or more after the same follow-up period (of note, the risk of recrudescence (corrected

by genotyping) after 42 days was 21% in the AS+SP arm of our pilot study). This difference would be detectable at 80% power with an effective sample size per arm of 203 episodes, which we will achieve if within-person correlation is not too strong. In addition, a difference of 6 % vs. 17% would be detectable with 80% power even at the minimum possible effective sample size of 150 per arm.

14.4 Analytical plan

14.4.1 Primary outcome

Statistical modeling and comparison of the number of treatments must take into account each participant's follow-up time, because more time at risk will obviously tend to produce more treatments regardless of which therapy is used. Estimating how the arms differ in terms of treatments per person-year of follow-up is therefore a useful summary. We will accomplish this by using Poisson regression, the standard approach for count data, to model the number of treatments, excluding the first. These models will include the logarithm of the follow-up time as an offset (an offset is similar to a predictor variable, but its coefficient is set to 1.0 instead of being estimated). Because the outcome variable is implicitly logarithmically transformed in Poisson regression, this will have the net effect of modeling log (treatments/follow-up), as desired. We will translate the fitted coefficients and their confidence bounds into percentage effects with the formula 100*{exp(coefficient)-1}. This approach is closely related to exponential survival models for analyzing events per follow-up time, but is better able to adjust for violated assumptions. Simpler methods for rates would require assuming that times between treatments are independent and identical exponential distributions. Testing for overdispersion in the Poisson regression can detect violations of these assumptions, and variances can be adjusted accordingly to produce valid p-values and confidence intervals [38]. Poisson regression also can include a random household effect, which may be desirable because different subjects from the same household may not be independent, and the initial sampling unit is a household.

14.4.2 Secondary outcomes

For the secondary analysis of treatment efficacy (with correction by genotyping), the unit of analysis will be an episode of malaria treated with a study drug. For all treatments, observation will continue until the time when a patient is retreated for malaria or follow-up ends (by exclusion or completion of the study). Outcomes of interest will include recrudescence and reinfection, as defined by genotyping, stratified by intervals of time from initiation of therapy. In addition to each type of failure, a composite outcome of any failure will also be analyzed. Repeated measures logistic regression will be used to model the influence of treatment arm and other factors on the probability of failure. Because episodes occurring in the same person may not be independent, even in models that include measured host factors, we will use the Statistical Analysis System's NLMIXED procedure to include a random person effect in the logistic regression models. Models of the two types of late failure will be restricted to those with initial treatment success and so will be conditional on that event, because it would not be clinically relevant to count early failure as lack of a late failure, a favorable outcome. Pairwise differences in treatment arms will be estimated and tested, without formal adjustment for multiple comparisons. Instead, interpretation will be cautious and not based solely on whether $p<0.05$ is achieved for any pairwise comparison; biologic plausibility (including whether the two regimens have a drug in common) and magnitudes of estimated effects will also be considered. In addition, an omnibus p-value for any treatment difference will be calculated by a likelihood ratio test.

For the secondary analysis of treatment safety and tolerability, the unit of analysis will be an episode of malaria treated with a study drug. Observation will continue until the time when a patient is retreated for malaria with the same study drug or follow-up ends (by subject withdrawal or completion of the study). Outcomes of interest will include various measures of adverse events stratified by intervals of time from initiation of therapy. Measures of adverse events of interest will include any adverse event, any serious adverse event, any adverse event stratified by type (sign, symptom, and laboratory abnormality), and any adverse event stratified by severity and relationship score. As above, repeated measures logistic regression will be used to model the influence of treatment arm and other factors on the probability of an adverse event.

It is possible that a patient may be diagnosed with their first episode of uncomplicated malaria in the Acute Care Unit, prior to being randomized and assigned a study treatment, and may initially be treated with non-study medications. If this occurs, the patient will be randomized at the time they present to the study clinic and will be treated with the appropriate study medications to complete a 3-day course of antimalarial treatment. In such cases, the malaria episode will be counted as an event in the primary analysis of treatment incidence density. However, such episodes will be excluded from secondary analyses of response to therapy for individual episodes of uncomplicated malaria.

14.5 Early analysis of data from Phase I - completed

We will perform an early analysis of efficacy and safety data collected during Phase I of the study. The rationale behind an early analysis includes both practical/public health considerations (see Section 8.5) and statistical considerations. Our initial sample size calculations were based on testing the hypotheses that treatment incidence density would differ among the 3 treatment arms and that the risk of treatment failure following individual study drug treatments would differ among the 3 treatment arms. There are several lines of evidence suggesting futility in observing significant differences in efficacy measures between the 3 treatment arms. Firstly, our preliminary results have shown that the observed incidence of malaria is lower than that predicted (observed = 1.62, predicted = 2.75), and because the incidence is highly skewed the standard deviation of normal approximation of primary outcome is higher than expected (2.9595 vs. 2.1). Based on our preliminary observed data we estimate that at the planned time of our early analysis 60% of study participants will have had at least one study drug treatment and we would have 80% power to detect a minimum difference in treatment incidence density of 31%. If we were to continue the study until all of the study participants were to have at least one treatment with study drug, we would have 80% power to detect a 21% or greater difference in treatment incidence density between the treatment arms. We do not feel that extending the time of analysis justifies the modest decrease in the minimum difference in treatment incidence density detectable. Secondly, our preliminary data on the risk of treatment failure following individual study drug treatments suggests little difference among the three treatment groups. At the time of the planned early analysis, we estimate that we will have given 873 study drug treatments (~291 per treatment arm) with a 28-day risk of treatment failure (uncorrected by genotyping, so including recrudescences and new infections) of 14.7% for all treatment groups combined. In a previous study of children aged 1-10 in Kampala, the same measure of treatment failure was 14% for both AQ+SP and AQ+AS, which represent 2 of the 3 treatment arms included in the current study. Extrapolating data from this prior study to our preliminary data would suggest a maximum difference in the 28 risk of treatment failure of 14% in the AQ+SP or AQ+AS arms versus 16.1% in the artemether-lumefantrine arm. A sample size of 4,550 treatments in each arm (alpha=0.05, power=0.80) would be required to detect such a small difference, which is far beyond what is achievable in this study. In summary, we believe that the strong public health benefit of unblinding our study for the purpose of expeditious data analysis and dissemination easily outweighs the predicted minor benefit of continuing the blinded study to increase sample size in this segment of the study.

15.0 ADVERSE EVENT MONITORING

15.1 Introduction

Our experience shows that, when defined as any untoward medical occurrence, adverse events occur commonly in an antimalarial drug efficacy trial, although the majority of events are likely due to the clinical course of malaria and other common non-malarial illnesses, and not to study medications. In this study, adverse event data will be captured at three levels based on the severity of the event. (1) All events: Basic information on timing and severity will be captured on the case record forms for all events that occur during study follow-up. (2) Events of moderate or greater severity: Additional information will be recorded on an Adverse Event Form by study physicians and subsequently

reviewed by the study coordinator. (3) Serious adverse events: Events will be reported individually using Serious Adverse Event Forms (initial and follow-up) for individual events.

At each patient encounter (including study clinic and routine home visits), a standardized system for assessing patient symptoms, physical exam findings, and laboratory abnormalities, based on grading of severity, will be used (Appendix D). Use of this monitoring system will allow for the systematic capture of all adverse events occurring throughout the study. Assessment and reporting of adverse events will begin with the first malaria episode treated with study medications. For each malaria episode, follow-up for safety will be conducted during the scheduled malaria follow-up visits (Days 1, 2, 3, 7, and 14), and at any unscheduled visit. Follow-up for adverse events reported during a given malaria episode will continue until the patient is re-treated with study medications, at which time a new cycle of adverse event assessment and reporting will begin, or the study ends.

The standardized system for monitoring adverse events was created based on our experience with malaria in Kampala, our review of adverse events related to antimalarials reported in the literature, package inserts of the study medications, and DMID guidelines for grading severity of events. The system actively defines and assesses adverse events, particularly serious toxicity, which has previously been reported in association with the study medications. As discussed in Section 17, these adverse events include hepatotoxicity (AQ, SP, and AS), bone marrow toxicity (AQ, SP and AS), severe skin reactions (SP), and neurological toxicity (AS and artemether). Considering these potential toxicities, our routine assessments will include blood counts (CBC and differential), liver enzyme measurements, skin examination, and neurological assessment.

15.2 Definitions

1) **Adverse event:** defined as "any untoward medical occurrence in a patient or clinical investigation subject administered a pharmaceutical product that does not necessarily have a causal relationship with this treatment" (ICH Guidelines E2A). An adverse event can further be broadly defined as any untoward deviation from baseline health which includes:

- Worsening of conditions present at the onset of the study
- Deterioration due to the primary disease
- Intercurrent illness
- Events related or possibly related to concomitant medications

(International Centers for Tropical Disease Research Network Investigator Manual, Monitoring and Reporting Adverse Events, 2003).

2) **Serious adverse event:** defined as an experience that results in any of the following outcomes:

- Death during the period of study follow-up
- Life-threatening experience (one that puts a patient at immediate risk of death at the time of the event)
- Inpatient hospitalization or prolongation of existing hospitalization during the period of study follow-up
- Persistent or significant disability or incapacity
- Congenital anomaly or birth defect
- Specific medical or surgical intervention to prevent one of the other serious outcomes listed in the definition.

3) **Expected adverse event:** To meet the guidelines for determining expectedness of an adverse event provided by our sponsor (NIH Division of Microbiology and Infectious Disease [DMID]) and our primary IRB (UCSF Committee on Human Research), two definitions of expectedness will be used in this study.

- **DMID Expectedness.** DMID defines an unexpected event as any adverse experience, the specificity or severity of which is not consistent with the current investigator brochure, or that has not been previously observed, i.e. included in drug package labeling (Appendix E), whether or not the event is anticipated because of the pharmacologic properties of the study agent. In this blinded study, 3 different treatment regimens including 4 different antimalarial preparations are to be evaluated. To be classified as an "expected" event according to the DMID definition, an event must be described in the package insert for ALL study medications. This will include AQ, AP, AS and Coartem in Phase I of the study and AQ, AS, and Coartem in Phase II. Given that that only elevation of ALT is reported for all of our study medications used in both Phase I and Phase II (Appendix E), the majority of adverse events will be considered "unexpected" according to the DMID definition. In Phase IIa, adverse events will be considered "unexpected" only if they are not listed or not consistent with the drug package labeling for Coartem.

- **CHR Expectedness.** The UCSF CHR defines an expected event as one that may be reasonably anticipated to occur as a result of the study procedures or study participation and should thus be described in the research proposal, the informed consent document, and investigator's brochure (when applicable), or is part of the normal disease process or progression. An unexpected event is one that may occur during the course of research participation that was unanticipated, or was more severe or more frequent than expected. An unexpected event is also one that results in subject withdrawal from study participation, is due to an overdose of study medication, or is due to a deviation from the approved protocol. In this study, events that are considered to be associated with malaria, including severe malaria, or which have been described on ANY package insert (Appendix E), will be considered "expected" according to the CHR definition. This will include AQ, SP, AS and Coartem in Phase I of the study, AQ, AS, and Coartem in Phase II, and only Coartem in Phase IIa. Other events, including those resulting in withdrawal, or related to overdose or a protocol deviation, will be considered "unexpected". The CHR definition of expectedness will be used to determine if expedited reporting of an unexpected event is indicated.

15.3 Baseline assessment

On Day 0 of all new malaria episodes, study physicians will ask about the presence of symptoms commonly associated with malaria and antimalarial treatment, and will perform a targeted physical examination. A severity grading system will be used to record information about symptoms and physical exam findings directly onto the case record form during the study clinic visits. Laboratory testing (CBC, differential, and ALT) will be performed on Days 0 and 14. Laboratory results and their severity grades will be transferred to the case record forms. The Day 0 values for symptoms, physical exam findings, and laboratory results will serve as the patient's baseline for that treatment episode.

15.4 Follow-up assessment

At each study clinic visit, including regularly scheduled malaria follow-up visits (Days 1, 2, 3, 7, and 14) and any unscheduled visits, patients will be assessed as on Day 0, and severity scores will be recorded on the case record forms. Study physicians will evaluate for the occurrence of adverse events by reviewing the case record form and comparing the severity grades assigned on that follow-up day to the values recorded for the previous visit. An increase in severity score will indicate that an adverse event has occurred. All events of increasing severity with a score of $\geq$ 2 will be reported on a separate form. If the severity of an event fluctuates during the standardized follow-up period, rising to $\geq$ 2 on more than one follow-up day, it will only be reported once (although the form may be updated to reflect any increase in the maximum severity or relationship).

15.5 Reporting of adverse events

Study physicians will record the following information on an Adverse Event Record Form:

1. Description of event
2. Date of event onset
3. Date event reported
4. Severity of the event
5. Relationship of the event to study medication
6. Should this be considered a Serious Adverse Event?
7. DMID Expectedness (is this event an elevation of ALT?)
8. CHR Expectedness (could this event be associated with malaria or is it reported on any package insert?)
9. Explanation (is there an alternative explanation for this event?)
10. Expedited reporting (indicated if event is serious or CHR unexpected with no other explanation for the event)
11. Initials of the person reporting the event

The recommended guidelines for AE reporting to NIH/DMID, the FDA, and UCSF CHR are summarized in the algorithm below.

15.5.1 Reporting of seizures

Following the January 2006 DSMB meeting, we were requesting to report all serious adverse events, regardless of whether the participant had been randomized and treated with study medications, due to concerns of the committee about the incidence of seizures in the study cohort. At the following DSMB meeting in July 2006, it was concluded that there was no significant difference found in seizures occurring in participants randomized and treated with study medications, to those who had not received study medications. However, we were requested to continue expedited reporting of seizures to the DMID, regardless of prior treatment with study medications. Of note, our other IRBs, only require expedited reporting of SAEs (including seizures) which are deemed as possibly, probably or definitely related to study medication. On August 3, 2007, reporting of events in participants not randomized to study medications was discontinued with permission from DMID, with the exception of seizures. On March 28, 2008, DMID granted us permission to report seizures periodically rather than via expedited reporting if the relationship of the event was deemed to be unrelated to study treatment. A log for periodic reporting of all seizures (found in Appendix J) within each reporting period will be submitted to the DMID every 6 months and in conjunction with the annual report when possible.

SAE REPORTING ALGORITHM

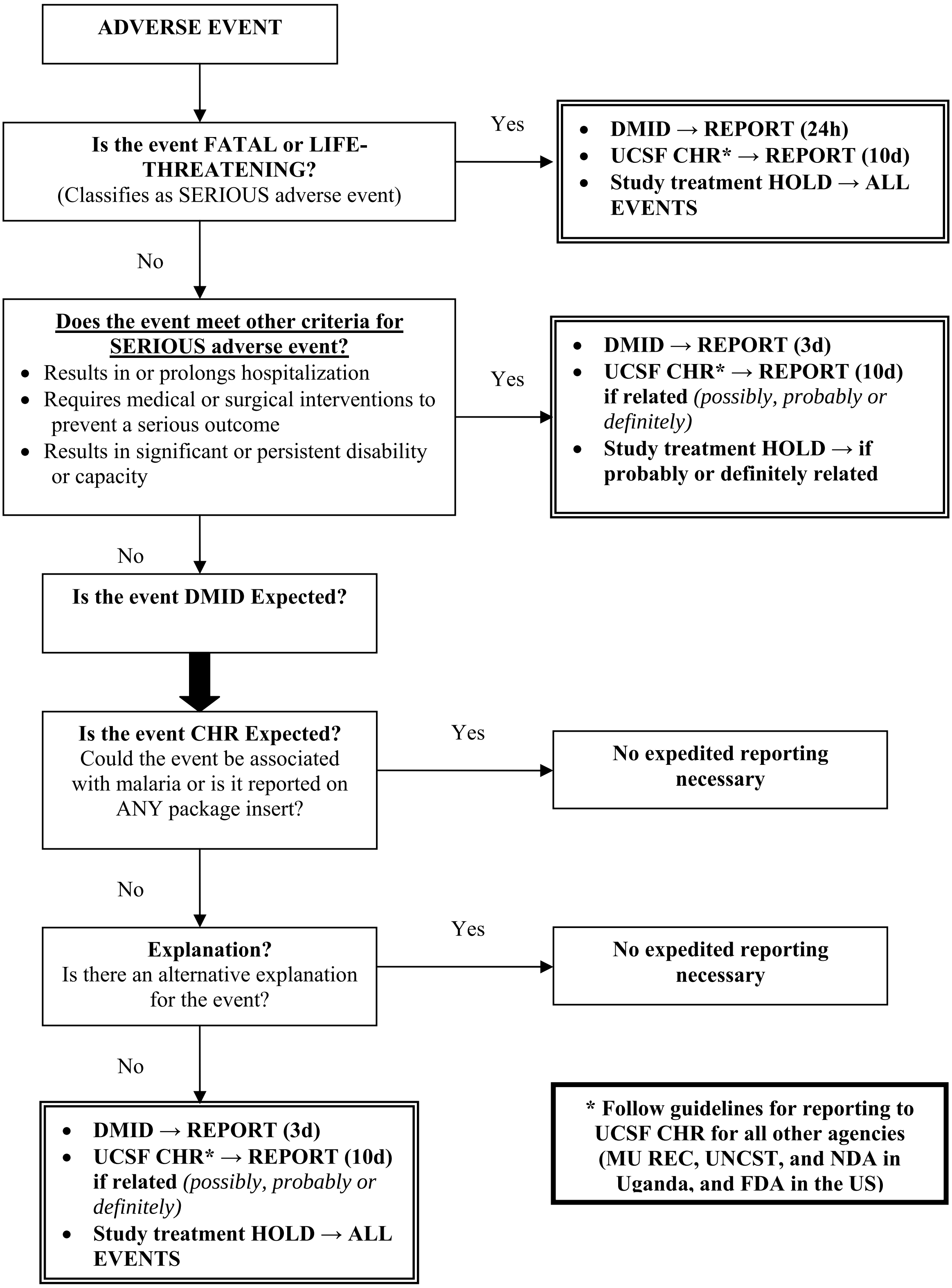

15.6 Follow-up of adverse events

Adverse event monitoring and follow-up will be done during the standardized 14-day follow-up period at all scheduled and unscheduled visits. On Day 14, a decision to continue active follow-up for any ongoing adverse events will be made at the discretion of the study physicians. At subsequent follow-up visits for new problems, subjects will undergo the same clinical assessment (symptoms, physical exam) and will be assessed for the occurrence of any new adverse events. Events previously reported during standardized malaria follow-up, or during subsequent visits, that had resolved or improved will be reported again if they have an increasing severity score of ≥ 2. If the patient is diagnosed with a

new malaria episode and is re-treated with study medications, a new cycle of adverse event assessment and reporting will begin.

Regarding follow-up of laboratory tests, in general, if the patient is asymptomatic and test results have a severity score of ≤ 2, the laboratory tests will be repeated according to the schedule in the protocol. If the patient is symptomatic, or if test results have a severity score of 3 or 4, the tests will be repeated at appropriate intervals based on good medical practice, additional tests will be ordered if necessary, and the patient's treatment will be changed if appropriate.

15.7 Study treatment hold

A decision to place a patient who experiences an SAE or a CHR unexpected event on "study treatment hold" by either stopping study medication and changing treatment to quinine (if the patient is actively receiving study medication for treatment of malaria), or by indicating that the patient should be treated with quinine instead of study medications in the case of a future diagnosis of malaria (if the patient is not current receiving treatment with study medication), will be made according to the following guidelines:

- **Life-threatening events** (severity grade = 4). If a patient experiences a life-threatening event, they will be immediately placed on study treatment hold, regardless of the suspected relationship of the event to the study medications.
- **Other SAEs.** If a patient experiences an SAE which is not life-threatening, they will be placed on study treatment hold if the event is initially judged to be probably or definitely related to the study medications.
- **CHR unexpected adverse events**. If a patient experiences a CHR unexpected event that has no other alternative explanation, they will be immediately placed on study treatment hold.

A patient may also be placed on "study treatment hold" if the study physicians feel that it is clinically indicated. For all SAEs and severe unexpected events, we will consult an independent local physician, who is not associated with the study, to review the case and to re-assess the relationship of the events to the study medication. If the independent physician judges the event to be not related or possibly related to the study medications, the patient will be taken off "study treatment hold" (when the study physicians feel that this is clinically appropriate) and will be treated with the combination regimen they were randomized to receive for all future episodes of uncomplicated malaria. If the event is felt to be probably or definitely related to the study medication, the patient will be excluded from the study (Section 10.11). If a subject is withdrawn from the study because of an event which is probably or definitely related to the study medication, plans for additional follow-up, including laboratory tests, will be individualized to each subject as appropriate for the clinical setting.

15.8 Routine visits

Subjects seen for the 90-day routine clinic visit will be questioned about symptoms and undergo a physical exam according to the standardized clinical assessment. Symptoms and physical exam findings will be graded by severity and recorded on the case record form. The forms will be reviewed and severity scores will be compared to those recorded at the subject's last encounter. If the subject is found to have any events with an increasing severity score of ≥ 2, these events will be reported (on the Adverse Event Record Form used for the most recent malaria episode) and the patient will be brought to the study clinic by the home visitor for further evaluation on the following day.

16.0 DATA AND SAFETY MONITORING

16.1 Data and safety monitoring board – Phase I & II

A data and safety monitoring board, assembled in conjunction with the NIH, will review the study protocol prior to implementation of the trial and will be convened to review the study periodically.

16.1.1 Data and safety monitoring board – Phase IIa

This trial became an open label trial on February 5, 2008. In agreement with the DMID, since this study is an open label trial, it will no longer require DSMB periodic review of the safety data. The Independent Safety Monitor and the DMID Medical Monitor will review safety data and consider a conference with the study team if a safety signal is identified or suspected in Phase IIa of the study.

16.2 Monitoring plan – Phase I & II

A three-year follow-up period is planned. Interim reports will be prepared when approximately one-third and two-thirds of the total projected follow-up time has been observed. Interim reports will contain information on study progress and data quality (including subject recruitment, patient follow-up, and protocol adherence), safety data (adverse events, serious adverse events), and efficacy data (treatment incidence density, clinical outcome at 14 days). Stopping guidelines will be based on comparisons among treatment groups of the primary efficacy outcome of treatment incidence density and the secondary outcome of the incidence of adverse events. Stopping guidelines based on secondary efficacy outcomes dependent on recrudescence and reinfection rates will not be provided, as these require the results of molecular testing that will not be available in a timely manner.

16.3 Stopping guidelines for Phase I- completed

Interpretation of results and decisions about discontinuation of any treatment arms will be made by a Data and Safety Monitoring Board (DSMB), using the suggested guidelines given here. We do not propose that the DSMB be strictly bound by pre-specified criteria, because of the complexity of the trade offs between safety, efficacy, and costs, and the possibility that new information will change considerations. Rather, consideration of stopping guidelines requires a reasoned judgment based on all information that is available at the time of data review. In the interim analyses, the AQ+SP treatment group will serve as the reference standard based on previous data collected at our study site and low cost. Stopping guidelines for the primary efficacy outcome will be provided (Table 5), but will only apply if AQ+SP is found to be superior to any of the other treatment groups (AQ+AS and coartemether). Superiority of these treatment groups in terms of efficacy over AQ+SP will not be considered sufficient reason to stop the study. The rationale for this approach is that factors other than efficacy, including cost and availability, are important when considering regimens as candidates for first-line therapy in a national antimalarial drug policy. The higher cost of the alternative treatment groups (AQ+AS and coartemether) may not justify their widespread use despite a statistically significant benefit compared to AQ+SP. Alternatively, statistically significant benefits of AQ+SP over any of the alternative treatment regimens in terms of efficacy would constitute criteria for early termination of any of the inferior alternative treatment groups. An additional measure for early termination of a treatment regimen will be based on the cumulative incidence of serious adverse events probably or definitely related to a study medication. Based on previous studies of uncomplicated malaria from our group, the incidence of severe malaria or danger signs was 26/1857 (1.4%) after 14 days of follow-up. A statistically significant (based on the lower bound of the confidence interval) excess of serious adverse events probably or definitely related to alternative study regimens (AQ+AS and Coartemether) relative to this baseline incidence measure (1.4%) will constitute criteria for early termination of any of the treatment regimens.

Participants randomized to a treatment regimen found to be inferior to AQ+SP or those assigned to a treatment regimen found to have an unacceptably high incidence of serious adverse events will be re-randomized to the remaining treatment groups for all future episodes of malaria for the duration of the study.

Table 5. Stopping rules for results of interim analyses

Outcome Category	Outcome	Unit of analysis	Comparison groups	Guidelines for statistical significance*
Efficacy	Treatment incidence density	Participant	AQ+SP vs alternatives†	First 1/3 p-value <0.0006<br>First 2/3 p-value <0.015
Safety	Serious AE probably or definitely related to study medication	Episode of uncomplicated malaria	Cumulative incidence in alternatives†	Lower bound of 95% CI > 1.4%

* P-values are O'Brien-Fleming values for two interim looks. Guidelines are not binding; a DSMB will make final decisions about discontinuation of any study arms.
† AQ+AS, coartemether

17.0 HUMAN SUBJECTS CONSIDERATIONS

17.1 Subject selection criteria

Study subjects will be a representative sample of children between 1 to 10 years of age from the Mulago III Parish of the Kawempe District of Kampala who meet our selection criteria and whose parents or guardians provide informed consent. The use of probability sampling will minimize selection bias and allow generalization of results to the larger target population. We plan to recruit only Ugandan residents and will recruit male and female children. This population is most appropriate for study, as malaria is primarily a pediatric disease in highly endemic countries, such as Uganda. In addition, children have less antimalarial immunity than adults, due to a smaller number of past infections, and so their analysis offers a clearer picture of drug resistance. The age 10 cut-off also avoids complexities of managing groups in different departments at Mulago Hospital, as, if necessary for after-hours visits, all participants will be appropriate for evaluation and treatment in the Pediatrics Department at Mulago Hospital, which treats children up to age 13, through the course of the study.

17.2 Risks and discomforts

17.2.1 Privacy

Care will be taken to protect the privacy of subjects, as described in this protocol. However, there is a risk that others may inadvertently see patients' medical information, and thus their privacy compromised.

17.2.2 Risks of randomization

This will be a randomized trial, and some treatment arms may prove to be more or less efficacious, more or less well tolerated, and/or more or less safe than others. Thus, there is the risk that patients will be randomized to less efficacious, less well tolerated, and/or less safe treatment regimens.

17.2.3 Finger sticks and venipuncture

Risks of these procedures include pain, transient bleeding and soft-tissue infection.

17.2.4 Risks of medications

17.2.4.1 Introduction

All of the antimalarial medications to be evaluated are registered or in the process of registration for treatment of malaria in Uganda, and no common serious toxicities are known [39-41]. However, rare serious toxicities are reported with older drugs (SP, AQ) and there are concerns with newer drugs that lack the years of post-marketing experience necessary for the identification of rare toxicities.

17.2.4.2 Amodiaquine (AQ)

AQ has been described as "very well tolerated" for routine use [40], and it was widely used for chemoprophylaxis against malaria in the past. However, prophylactic use was discontinued due to rare instances of agranulocytosis, aplastic anemia, and hepatotoxicity, principally associated with use for malarial chemoprophylaxis in travelers [4, 40]. Side effects listed as occasional on the package insert (Pfizer/Parke-Davis, Senegal) are nausea, vomiting, diarrhea, lethargy, agranulocytosis and other blood dyscrasias, hepatitis, and peripheral neuropathy. Reported rates of serious reactions to AQ in the UK were 1:2100 blood dyscrasias, 1:31,000 deaths from blood dyscrasias, and 1:15,650 serious hepatotoxicity [42]. Toxicities with short-term use for treatment are expected to be much lower, although data are limited [4-8]. In a review of 40 published and unpublished clinical trials, no severe or life-threatening adverse event was noted [4]. Considering tolerability in 488 AQ-treated patients, gastrointestinal toxicities and pruritis were most commonly reported, and the incidence of adverse events was similar among patients treated with AQ, CQ, and SP [4]. At our study site in Uganda, no serious toxicities were observed with AQ monotherapy (131 treatments) [8].

17.2.4.3 Sulfadoxine-pyrimethamine (SP)

SP has generally been the preferred replacement for CQ for the treatment of uncomplicated malaria in Africa. Although technically a combination regimen, SP is generally considered a single antimalarial agent, as its success depends on the synergistic action of its two component inhibitors of folate synthesis. SP is approved in the USA for the treatment of falciparum malaria and for chemoprophylaxis against malaria in travelers, but it is no longer recommended for this second use due to rare, but serious toxicity. Adverse reactions listed on the SP package insert (Roche, USA) are blood dyscrasias (agranulocytosis, aplastic anemia, thrombocytopenia), allergic reactions (erythema multiforme and other dermatological conditions), gastrointestinal reactions (glossitis, stomatitis, nausea, emesis, abdominal pain, hepatitis, diarrhea), central nervous system reactions (headache, peripheral neuritis, convulsions, ataxia, hallucinations), respiratory reactions (pulmonary infiltrates), and miscellaneous reactions (fever, chills, nephrosis); based on widespread experience with the drug, all of these reactions appear to be uncommon or rare with short-term therapeutic use. The best-documented severe adverse effects with SP are cutaneous reactions, primarily noted when SP was used for long-term chemoprophylaxis in non-African populations. Reported rates of serious reactions to SP in the UK, with long-term use for chemoprophylaxis, were 1:2100, with 1:4900 serious dermatological reactions and 1:11,100 deaths [42]. Estimated rates of toxicity in the US were 1:5000-8000 severe cutaneous reactions and 1:11,000-25,000 deaths [43]. Clinical experience suggests that risks of severe toxicity are much lower with malaria treatment regimens in Africa. Overall, the risk of severe reactions occurring in developing countries with single-dose SP treatment has been estimated at 0.1 per million [44]. Of note, the AQ+SP arm of the study is being dropped and SP will no longer be received by study participants in Phase II of the study.

17.2.4.4 Artemisinins

Artemisinin derivatives have now been extensively studied, and they are remarkable for a lack of serious toxicity when used for the treatment of malaria [13]. Considering all artemisinins, 15% (12,463) of the patients enrolled in all published antimalarial drug trials over the past 50 years have received an artemisinin compound, and there are more trials on these compounds than on any other antimalarials (N. White, unpublished communication). In addition to formal studies, artemisinins have now been widely used, with well over a million treatments, mostly of AS, in Southeast Asia. The only serious toxicity which has emerged in detailed prospective clinical evaluations is a low risk of type 1 hypersensitivity reactions (estimated risk 1:2833, 95% CI 1:1362-1:6944) [45]. Electrocardiograms and detailed neurological, audiometric, and neurophysiological tests have failed to show any evidence for cardiac or neurological toxicity in humans (see below for more details) [46-49]. Considering oral AS, adverse events appear to be rare. The package insert for AS (Sanofi-Winthrop, France) notes only two potential laboratory abnormalities "in a few cases", lowering of reticulocyte count and slight increases in transaminases.

Animal studies have led to some concerns over artemisinins, particularly regarding cardiac and neurological effects. As slight QT prolongation was observed in dogs treated with high doses, detailed electrocardiographic studies have been conducted in humans during treatment for falciparum malaria [23, 47-50]. Taking into account effects of malaria, no significant effects of artemisinins on the QT interval were identified.

The neurological effects of artemisinins have been very extensively studied. In mice, rats, dogs, and monkeys, high dosages of intramuscular artemether and arteether produce an unusual and selective pattern of damage to certain brainstem nuclei, particularly those of the auditory and vestibular systems [51-64]. AS is transformed in vivo to dihydroartemisinin, which is the most neurotoxic of the artemisinin derivatives [65-67]. However, in the animal models, orally administered AS and dihydroartemisinin are considerably less neurotoxic than intramuscular artemether or arteether. Differences in toxicity are explained by differences in pharmacokinetics of different compounds and different routes of administration [55, 59, 61, 63]. Neurotoxicity results from the long-lasting blood concentrations that follow intramuscular injection of the oil-soluble compounds, artemether and arteether. Oral administration of artemether or arteether, which provides much more rapid absorption and elimination than intramuscular dosing, leads to markedly less neurotoxicity in mice, although oral artemether can be made more neurotoxic by giving the drug in small repeated doses to simulate the constant exposure that follows intramuscular injection [61]. Artesunate is much less toxic than arteether in rats when administered intramuscularly [62] or orally [56, 63, 68]. Importantly, with high dose intramuscular injections of artemether and arteether, clinical assessment of mice was a sensitive indicator of neurotoxicity; no mice with normal clinical exams showed histopathology [67].

The artemisinin derivatives are remarkably well tolerated in humans. In a clinical safety review of 108 studies including 9,241 patients, no serious adverse events or significant toxicity was reported [69]. In addition, a systematic review of artemisinin derivatives for treating uncomplicated malaria, including 41 studies of 5,240 patients, showed no evidence of harmful effects related to artemisinin derivatives [70]. Clinical studies have shown no convincing evidence for neurotoxicity after treatment with artemisinin derivatives, though neurological effects of acute malaria are common. One letter described ataxia and slurred speech after AS therapy, but these findings were consistent with the course of severe malaria [71]. To specifically evaluate for potential artemisinin-associated auditory toxicity in humans, van Vugt et al. performed clinical neurological evaluations, audiometry and early latency auditory evoked responses in 79 patients treated with multiple doses of artemether or artesunate and 79 matched controls in Thailand, and no evidence of auditory toxicity was detected [48]. Comparisons of patients who had received multiple courses of artemisinin derivatives with age-matched untreated controls showed no significant differences in clinical, audiometric, or auditory evoked potential measurements [46, 48]. Even considering the most worrisome dosing regimen, there is no evidence that clinical use of intramuscular artemether has caused neurotoxicity. In a new report, four independent neuropathologists examined the brains of patients who died after treatment with intramuscular artemether, and there was no evidence for the characteristic pattern of neuropathological change seen in the animal studies [72]. These results suggest a wide margin of safety for artemisinins in clinical use, particularly when given orally, particularly for water soluble compounds, and most particularly for the most widely studied water-soluble agent, AS.

17.2.4.5 Artemether-Lumefantrine (Coartemether)

Coartemether (Coartem and Riamet; Novartis) has been extensively studied through GCP standardized preclinical and clinical trials and was added to the WHO Essential Medicines List in 2002 [27]. It has been approved for use against malaria in both developing and developed (e.g. Switzerland) countries. The drug appears to be very well tolerated, especially in comparison to other antimalarials and antimalarial combinations including choloroquine, quinine, and mefloquine+artesunate. A clinical safety review of children under 12 years of age showed that the most common adverse events were abdominal pain, cough, anorexia, headache, vomiting, and diarrhea (all seen in 5-12% of subjects; Novartis monograph, 3rd ed., Jan 2004; www.novartis.com).

An integrated review of toxicity in 1869 patients (611 under age 13) showed the most commonly reported adverse events were gastrointestinal disturbances (abdominal pain, anorexia, nausea, vomiting diarrhea), headache, and dizziness (Table 6). Rash and pruritis were reported in <2% of patients. No serious or persistent neurological toxicities were linked to coartemether therapy. Of 20 severe adverse events in 1869 patients, 19 were likely attributable to underlying malaria or concomitant illness, and one was possibly related to coartemether use (hemolytic anemia in a 35-year-old 13 days after the last administered dose)[41]. One concern addressed in studies of coartemether was possible cardiac arrhythmogenic potential based on similarities in the chemical structures of lumefantrine and halofantrine. Halofantrine can cause defects in cardiac conduction, particularly a marked QT prolongation that can produce arrhythmias. In 713 patients treated with lumefantrine and followed with serial electrocardiograms, no adverse clinical cardiac events were recorded. Although trials have been limited to date, no serious cardiotoxicity or neurotoxicity has been reported with the use of coartemether [22, 39, 48, 73].

17.2.4.6 Combination antimalarial therapy

There is broad consensus that combination therapies offer the optimal means of treating malaria in Africa, and many insist that all new antimalarial regimens should include multiple drugs. However, although combination therapy is now standard in many countries in Asia, South America, and Africa (including Uganda), limited data on safety of the combinations are available. A strength of our project will be its ability to rigorously assess the safety of antimalarial combinations that are also used or soon to be used (outside of well-monitored studies) in many parts of Africa.

AQ+SP. A systematic review of 3 older studies that included AQ+SP reported no serious adverse events [74]. In three published studies from our study site in Kampala, a total of 941 AQ+SP treatments were administered without the occurrence of severe adverse events (Table 6) [3, 8, 28]. Another group has also studied AQ+SP in 59 children with malaria in Tanzania and adverse events other than those expected for acute malaria were not identified [30]. In a recent study from Kampala, which compared three antimalarial combination therapies (Staedke, unpublished), rigorous surveillance for adverse events, including laboratory testing, was done to evaluate drug safety and tolerability. Of 132 patients treated with AQ+SP, 6 experienced serious adverse events (defined as events that resulted in hospitalization, required medical intervention, or were life-threatening). Most of the events were attributable to severe malaria and included convulsions (3 patients) and vomiting (1 patient). Three serious hematologic events that were not associated with severe malaria or other illnesses occurred in a single patient. Thrombocytopenia, neutropenia, anemia were reported in one AQ+SP treated patient who was successfully treated for malaria. Severe thrombocytopenia (33,000/mm^3) was present at enrollment and persisted through study follow-up, becoming life-threatening. Severe neutropenia and anemia subsequently developed and the patient became transfusion-dependent. A bone marrow biopsy revealed hypoplastic marrow of undetermined etiology with all cell lines present. Additional severe laboratory-related adverse events reported in the AQ+SP group included transient asymptomatic neutropenia (1 patient), and elevation of ALT associated with clinical hepatitis (1 patient), which resolved spontaneously by Day 28. As above, AQ+SP will be discontinued in Phase II of the study.

AQ+AS. In a 3-center study of 470 treatments with AQ+AS in Kenya, Senegal, and Gabon, symptoms consistent with malaria were common, but other adverse events were uncommon, and included vomiting in 1.3%, pruritis in 1.9%, and skin rash in 0.2%. Seven serious adverse events were all consistent with malaria or other illnesses and not deemed related to study drugs [32]. Recently in Kampala, AQ+AS was well-tolerated. Of 133 patients treated with AQ+AS, no serious adverse events which were likely to be related to the study treatment were reported. Additional severe laboratory-related adverse events in the AQ+AS group included anemia in one patient (Staedke, unpublished).

Other combinations. Table 6 outlines reported adverse events from the most recent studies of each of the study combination regimens.

Table 6. Adverse Events reported in previous studies for antimalarial study combinations

Drug Combination	Country	n	AE reported
**AQ+SP [79]**	Uganda	534	fever (15%); pruritis (8%); anorexia (7%); weakness (6%); cough, vomiting (2%); headache, diarrhea, coryza (1%)
**AQ+SP [80]**	Uganda	129	cough (37%); viral syndrome (31%); fever (27%); vomiting (24%); pruritis, anorexia, diarrhea, weakness (18%); headache (17%)
**AQ+SP [28]**	Uganda	172	pruritis, nausea/vomiting (24%); diarrhea (11%); neutropenia (9%); abdominal pain (6%); cough (5%); anorexia, malaise (3%); headache (2%); rash (1%)
**AQ+SP [3]**	Uganda	215	no reported serious adverse events
**AQ+AS†**	Uganda	130	cough (34%); viral syndrome, fever (26%); vomiting, diarrhea (21%); weakness (18%); pruritis (14%); headache (12%)
**AQ+AS [32]**	Kenya, Senegal, Gabon	470	vomiting (1.3%), pruritis (1.9%), skin rash (0.2%), other reported AE: weakness, headache, dizziness, anorexia, nausea, abdominal pain, diarrhea
**Coartemether [75]**	Thailand	25 (6 dose)	for total study (adults and children) AEs included: nervous system related [dizziness, sleep disorder, headache] (6%), gastrointestinal [anorexia, nausea, abdominal pain, hepatomegal, vomiting] (12.7%), 1 case tremor (day 1), 2 cases hypoesthesia, 1 SAE 12d after dosing, fever & coma (for 4d), full recovery
**Coartemether [76]**	Thailand	12 (4 dose)<br>18 (6 dose)<br>13 (6 dose)	vomiting, headache, anorexia, asthenia, arthralgia, myalgia, dizziness; no related SAE
**Coartemether [77]**	Gambia	144 (4 dose)	diarrhea, vomiting, coughing
**Coartemether [78]**	Tanzania	260 (4 dose)	abdominal pain, fatigue, headache, vomiting, anorexia, diarrhea, sleeping disorders

17.2.4.7 Quinine

Quinine is the standard drug for the treatment of severe malaria throughout Africa and is also the standard drug for the treatment of falciparum malaria in the U.S. It will be used to treat patients with symptomatic falciparum malaria at the onset of the study and it will be the treatment for patients who fail therapy with any study regimen. The use of quinine to treat infections that fail other therapies is in accordance with Ugandan malaria treatment policy. Quinine can commonly cause tinnitus, headache, nausea, dizziness, flushing, and visual disturbances. These symptoms, termed "cinchonism", do not warrant discontinuing therapy unless they are severe. Less common toxicities include vomiting, diarrhea, and abdominal pain. Rare toxicities include skin rashes, urticaria, angioedema, bronchospasm, hemotologic abnormalities (hemolysis, leukopenia, agranulocytosis, and thrombocytopenia). Quinine can cause hypoglycemia, especially in pregnancy. Cardiovascular toxicity is seen principally with intravenous quinine, which will not be used in our study.

17.2.4.8 Clindamycin

Clindamycin will be used in conjunction with quinine only to treat patients who fail quinine therapy after failure of study regimens to treat malaria. Common adverse events with clindamycin are diarrhea, nausea, and skin rashes. Much less commonly, severe diarrhea, including pseudomembranous colitis, can occur. Uncommon toxicities also include hepatic abnormalities and neutropenia.

17.2.4.9 Acetaminophen

This drug will be used as an antipyretic during acute episodes of malaria. It is probably the most widely used analgesic in the world and is generally very well tolerated. The most common serious toxicity of acetaminophen is hepatotoxicity, but this is nearly always a result of ingestion of dosages far above those that are standard (as will be used in our study). Transient mild increases of hepatic enzymes can also be seen.

17.2.4.10 Mebendazole
This drug is generally very well tolerated for short-term treatment of helminth infections. Infrequent mild toxicities include mild nausea, vomiting, diarrhea, and abdominal pain. Rare side effects, usually with high-dose therapy (not to be used in this study), are hypersensitivity reactions (rash, urticaria), agranulocytosis, alopecia, and elevation of liver enzymes.

17.2.4.11 Iron sulfate
Iron sulfate is a routine therapy to replace iron in anemic patients. Toxicities include heartburn, nausea, upper gastric discomfort, constipation, and diarrhea.

17.3 Treatment and compensation for injury

The usual services offered at the study clinic and at Mulago Hospital will be available in case of any injury related to the study. Care will be provided free of charge for injuries related to study participation using available funds.

17.4 Alternatives

Individuals whose parents or guardians choose not to participate in this study will not be enrolled. They will receive standard care for medical problems as they arise at the Mulago Hospital outpatient department or other medical facilities in Kampala.

17.5 Costs to the subject

None

17.6 Reimbursement of subjects

Subjects will not be paid for their participation in the study. We will provide all routine medical care, including evaluations, medications available in our clinic, and cost of any transportation free of charge. In addition, we will reimburse the cost of consultation for referrals made by study physicians to other clinics and services within the Mulago Hospital Complex, and visits to the Acute Care Unit. We anticipate reimbursing the cost of most diagnostic tests (including laboratory test, X-rays, and ultrasounds) and medications resulting from these referrals, using available funds. However, reimbursement of all diagnostic tests and treatment recommended outside the study clinic cannot be guaranteed in all circumstances.

17.7 Confidentiality of records

Parents and guardians will be informed that participation in a research study may involve a loss of privacy. All records will be kept as confidential as possible. Patients will be identified primarily by their study number and patient names will not be entered into the computerized database. All patient record forms will be kept in individual files in a secured filing cabinet in the study clinic. Additional records will be kept in the clinical and laboratory record books, which will be stored in the central study laboratory in Makerere University Medical School. No individual identities will be used in any reports or publications resulting from the study.

18.0 QUALIFICATIONS OF INVESTIGATORS

Dr. Philip Rosenthal (Principal Investigator) is Professor in the Department of Medicine, UCSF, is board certified in Medicine and Infectious Diseases, has maintained a strong research and clinical interest in malaria for many years, and has co-directed clinical malaria studies in Uganda since 1998.

Dr. Moses Kamya (Co-Investigator) is Senior Lecturer in the Dept. of Medicine, Makerere University, Kampala, Uganda, is an expert on malaria and other tropical diseases, and has conducted clinical research on malaria and other tropical infectious diseases for many years.

Dr. Grant Dorsey (Co-Investigator) is an Assistant Professor in the Department of Medicine, UCSF, is board-certified in Medicine and Infectious Diseases, received a PhD in Epidemiology from University California, Berkeley, and has conducted clinical and epidemiological studies of malaria in Uganda since 1998.

Dr. Sarah Staedke (Co-Investigator) is a Clinical Senior Lecturer at the London School of Hygiene & Tropical Medicine, is board-certified in Medicine and Infectious Diseases, is licensed to practice medicine in Uganda, received a Diploma in Tropical Medicine and Hygiene from the London School of Tropical Medicine, and has conducted clinical and epidemiological studies of malaria in Uganda since 1998.

Dr. Jeffrey Martin (Co-Investigator) is an Associate Professor in the Department of Epidemiology and Biostatistics, UCSF, is board certified in Medicine and Infectious diseases, trained in epidemiology and biostatistics, and has considerable experience in the conduct of human subjects research, particularly in the area of human herpesvirus 8 infection.

Dr. Lisa Butler (Co-Investigator) is a Postdoctoral Research Fellow in the Department of Medicine, UCSF. She has conducted epidemiologic studies of human herpesvirus 8 infection in sub-Saharan Africa since 2002.

Dr. Bryan Greenhouse (Co-Investigator) is an Adjunct Assistant Professor in the Department of Medicine, UCSF, is board-certified in Medicine and Infectious diseases, and has conducted translational studies of malaria since 2005.

Dr. Chandy John (Co-Investigator) is an Associate Professor of Pediatrics in the Division of Pediatric Infectious Diseases and the Director of the Global Pediatrics Program at the University of Minnesota. Dr. John's areas of interest include research in malaria immunology and epidemiology, and education in global health and infectious disease. He has expertise in investigating how immune responses to P. falciparum antigens relate to risk of infection and disease.

Other clinicians and staff participating in the study will be supervised by the personnel listed above to ensure that they are qualified to manage clinical malaria studies and all aspects of the study procedures discussed above.

19.0 SPECIAL REQUIREMENTS

This study will receive approval from the UCSF Committee on Human Research, the Makerere University Medical School Faculty IRB, Ugandan National Council of Science and Technology, and the NIH / DMID International Clinical Studies Review Committee before it is initiated.

20.0 REFERENCES

1. Rogier C, Tall A, Diagne N, Fontenille D, Spiegel A and Trape JF. Plasmodium falciparum clinical malaria: lessons from longitudinal studies in Senegal. Parassitologia 1999;41:255-9.
2. White N. The assessment of antimalarial drug efficacy. Trends Parasitol 2002;18:458-464

3. Dorsey G, Njama D, Kamya MR, Cattamanchi A, Kyabayinze D, Staedke SG, Gasasira A and Rosenthal PJ. Sulfadoxine/pyrimethamine alone or with amodiaquine or artesunate for treatment of uncomplicated malaria: a longitudinal randomised trial. Lancet 2002;360:2031-8
4. Olliaro P, Nevill C, LeBras J, Ringwald P, Mussano P, Garner P and Brausseur P. Systematic review of amodiaquine treatment in uncomplicated malaria. Lancet 1996;348:1196-1201
5. van Dillen J, Custers M, Wensink A, Wouters B, van Voorthuizen T, Voorn W, Khan B, Muller L and Nevill C. A comparison of amodiaquine and sulfadoxine-pyrimethamine as first-line treatment of falciparum malaria in Kenya. Trans R Soc Trop Med Hyg 1999;93:185-8
6. Brasseur P, Guiguemde R, Diallo S, Guiyedi V, Kombila M, Ringwald P and Olliaro P. Amodiaquine remains effective for treating uncomplicated malaria in west and central Africa. Trans R Soc Trop Med Hyg 1999;93:645-50
7. Gorissen E, Ashruf G, Lamboo M, Bennebroek J, Gikunda S, Mbaruku G and Kager PA. In vivo efficacy study of amodiaquine and sulfadoxine/ pyrimethamine in Kibwezi, Kenya and Kigoma, Tanzania. Trop Med Int Health 2000;5:459-63.
8. Staedke SG, Kamya MR, Dorsey G, Gasasira A, Ndeezi G, Charlebois ED and Rosenthal PJ. Amodiaquine, sulfadoxine/pyrimethamine, and combination therapy for treatment of uncomplicated falciparum malaria in Kampala, Uganda: a randomised trial. Lancet 2001;358:368-74
9. Dorsey G, Kamya MR, Ndeezi G, Babirye JN, Phares CR, Olson JE, Katabira ET and Rosenthal PJ. Predictors of chloroquine treatment failure in children and adults with falciparum malaria in Kampala, Uganda. Am J Trop Med Hyg 2000;62:686-92.
10. Bloland PB, Lackritz EM, Kazembe PN, Were JBO, Steketee R and Campbell CC. Beyond chloroquine: implications of drug resistance for evaluating malaria therapy efficacy and treatment policy in Africa. Journal of Infectious Diseases 1993;167:932-927
11. Mberu EK, Mosobo MK, Nzila AM, Kokwaro GO, Sibley CH and Watkins WM. The changing in vitro susceptibility pattern to pyrimethamine/sulfadoxine in Plasmodium falciparum field isolates from Kilifi, Kenya. Am J Trop Med Hyg 2000;62:396-401
12. AFRO. Global AMDP database.
13. Meshnick SR, Taylor TE and Kamchonwongpaisan S. Artemisinin and the antimalarial endoperoxides: from herbal remedy to targeted chemotherapy. Microbiol Rev 1996;60:301-15
14. Li GQ, Guo XB, Fu LC, Jian HX and Wang XH. Clinical trials of artemisinin and its derivatives in the treatment of malaria in China. Trans R Soc Trop Med Hyg 1994;88 Suppl 1:S5-6
15. Looareesuwan S. Overview of clinical studies on artemisinin derivatives in Thailand. Trans R Soc Trop Med Hyg 1994;88 Suppl 1:S9-11
16. Hien TT. An overview of the clinical use of artemisinin and its derivatives in the treatment of falciparum malaria in Viet Nam. Trans R Soc Trop Med Hyg 1994;88 Suppl 1:S7-8
17. Brockman A, Price RN, van Vugt M, Heppner DG, Walsh D, Sookto P, Wimonwattrawatee T, Looareesuwan S, White NJ and Nosten F. Plasmodium falciparum antimalarial drug susceptibility on the north-western border of Thailand during five years of extensive use of artesunate-mefloquine. Trans R Soc Trop Med Hyg 2000;94:537-44
18. Price RN, Nosten F, Luxemburger C, van Vugt M, Phaipun L, Chongsuphajaisiddhi T and White NJ. Artesunate/mefloquine treatment of multi-drug resistant falciparum malaria. Trans R Soc Trop Med Hyg 1997;91:574-7
19. White NJ. Delaying antimalarial drug resistance with combination chemotherapy. Parassitologia 1999;41:301-8.
20. White NJ, Nosten F, Looareesuwan S, Watkins WM, Marsh K, Snow RW, Kokwaro G, Ouma J, Hien TT, Molyneux ME, Taylor TE, Newbold CI, Ruebush TK, Danis M, Greenwood BM, Anderson RM and Olliaro P. Averting a malaria disaster. Lancet 1999;353:1965-67
21. Attaran A, Barnes KI, Curtis C, d'Alessandro U, Fanello CI, Galinski MR, Kokwaro G, Looareesuwan S, Makanga M, Mutabingwa TK, Talisuna A, Francois Trape J and Watkins WM. WHO, the Global Fund, and medical malpractice in malaria treatment. Lancet 2004;363:237-40
22. Bindschedler M, Lefevre G, Degen P and Sioufi A. Comparison of the cardiac effects of the antimalarials co-artemether and halofantrine in healthy participants. Am J Trop Med Hyg 2002;66:293-8

23. van Vugt M, Ezzet F, Nosten F, Gathmann I, Wilairatana P, Looareesuwan S and White NJ. No evidence of cardiotoxicity during antimalarial treatment with artemether-lumefantrine. Am J Trop Med Hyg 1999;61:964-7
24. Lefevre G, Looareesuwan S, Treeprasertsuk S, Krudsood S, Silachamroon U, Gathmann I, Mull R and Bakshi R. A clinical and pharmacokinetic trial of six doses of artemether-lumefantrine for multidrug-resistant Plasmodium falciparum malaria in Thailand. Am J Trop Med Hyg 2001;64:247-56
25. Ezzet F, van Vugt M, Nosten F, Looareesuwan S and White NJ. Pharmacokinetics and pharmacodynamics of lumefantrine (benflumetol) in acute falciparum malaria. Antimicrob Agents Chemother 2000;44:697-704
26. Snow RW, Eckert E and Teklehaimanot A. Estimating the needs for artesunate-based combination therapy for malaria case-management in Africa. Trends Parasitol 2003;19:363-9
27. World Health Organization. Position of WHO's Roll Back Malaria Department on malaria treatment policy.
28. Gasasira AF, Dorsey G, Nzarubara B, Staedke SG, Nassali A, Rosenthal PJ and Kamya MR. Comparative efficacy of aminoquinoline-antifolate combinations for the treatment of uncomplicated falciparum malaria in Kampala, Uganda. Am J Trop Med Hyg 2003;68:127-32
29. Staedke SG, Mpimbaza A, Kamya MR, Nzarubara B, Dorsey G and Rosenthal PJ. Combination therapies for uncomplicated falciparum malaria in Kampala, Uganda: a randomized clinical trial. submitted for publication 2004
30. Schellenberg D, Kahigwa E, Drakeley C, Malende A, Wigayi J, Msokame C, Aponte JJ, Tanner M, Mshinda H, Menendez C and Alonso PL. The safety and efficacy of sulfadoxine-pyrimethamine, amodiaquine, and their combination in the treatment of uncomplicated Plasmodium falciparum malaria. Am J Trop Med Hyg 2002;67:17-23
31. Rwagacondo CE, Niyitegeka F, Sarushi J, Karema C, Mugisha V, Dujardin JC, Van Overmeir C, van den Ende J and D'Alessandro U. Efficacy of amodiaquine alone and combined with sulfadoxine-pyrimethamine and of sulfadoxine pyrimethamine combined with artesunate. Am J Trop Med Hyg 2003;68:743-7
32. Adjuik M, Agnamey P, Babiker A, Borrmann S, Brasseur P, Cisse M, Cobelens F, Diallo S, Faucher JF, Garner P, Gikunda S, Kremsner PG, Krishna S, Lell B, Loolpapit M, Matsiegui PB, Missinou MA, Mwanza J, Ntoumi F, Olliaro P, Osimbo P, Rezbach P, Some E and Taylor WR. Amodiaquine-artesunate versus amodiaquine for uncomplicated Plasmodium falciparum malaria in African children: a randomised, multicentre trial. Lancet 2002;359:1365-72
33. de Vries PJ, Dien TK. Clinical pharmacology and therapeutic potential of artemisinin and its derivatives in the treatment of malaria. Drugs 1996;52:818-36
34. Bunnag D, Viravan C, Looareesuwan S, Karbwang J and Harinasuta T. Clinical trial of artesunate and artemether on multidrug resistant falciparum malaria in Thailand. A preliminary report. Southeast Asian J Trop Med Public Health 1991;22:380-5
35. Njama D, Dorsey G, Guwatudde D, Kigonya K, Greenhouse B, Musisi S and Kamya MR. Urban malaria: primary caregivers' knowledge, attitudes, practices and predictors of malaria incidence in a cohort of Ugandan children. Trop Med Int Health 2003;8:685-92
36. Severe falciparum malaria. World Health Organization, Communicable Diseases Cluster. Trans R Soc Trop Med Hyg 2000;94 Suppl 1:S1-90
37. World Health Organization. Assessment and monitoring of antimalarial drug efficacy for the treatment of uncomplicated falciparum malaria:http://www.emro.who.int/rbm/publications/protocolwho.pdf
38. McCullagh P, Nelder JA. Generalized Linear Models. London: Chapman and Hall, 1989
39. Omari AA, Gamble C and Garner P. Artemether-lumefantrine for uncomplicated malaria: a systematic review. Trop Med Int Health 2004;9:192-9
40. Luzzi GA, Peto TEA. Adverse effects of antimalarials. Drug Safety 1993;8:295-311
41. Bakshi R, Hermeling-Fritz I, Gathmann I and Alteri E. An integrated assessment of the clinical safety of artemether-lumefantrine: a new oral fixed-dose combination antimalarial drug. Trans R Soc Trop Med Hyg 2000;94:419-24
42. Phillips-Howard PA, West LJ. Serious adverse drug reactions to pyrimethamine-sulphadoxine, pyrimethamine-dapsone and to amodiaquine in Britain. J R Soc Med 1990;83:82-5.

43. Miller KD, Lobel HO, Satriale RF, Kuritsky JN, Stern R and Campbell CC. Severe cutaneous reactions among American travelers using pyrimethamine-sulfadoxine (Fansidar) for malaria prophylaxis. Am J Trop Med Hyg 1986;35:451-8
44. Sturchler D, Mittelholzer ML and Kerr L. How frequent are notified severe cutaneous adverse reactions to Fansidar? Drug Saf 1993;8:160-8
45. Leonardi E, Gilvary G, White NJ and Nosten F. Severe allergic reactions to oral artesunate: a report of two cases. Trans R Soc Trop Med Hyg 2001;95:182-3
46. Kissinger E, Hien TT, Hung NT, Nam ND, Tuyen NL, Dinh BV, Mann C, Phu NH, Loc PP, Simpson JA, White NJ and Farrar JJ. Clinical and neurophysiological study of the effects of multiple doses of artemisinin on brain-stem function in Vietnamese patients. Am J Trop Med Hyg 2000;63:48-55
47. Tran TH, Day NP, Nguyen HP, Nguyen TH, Pham PL, Dinh XS, Ly VC, Ha V, Waller D, Peto TE and White NJ. A controlled trial of artemether or quinine in Vietnamese adults with severe falciparum malaria. N Engl J Med 1996;335:76-83
48. van Vugt M, Angus BJ, Price RN, Mann C, Simpson JA, Poletto C, Htoo SE, Looareesuwan S, White NJ and Nosten F. A case-control auditory evaluation of patients treated with artemisinin derivatives for multidrug-resistant Plasmodium falciparum malaria. Am J Trop Med Hyg 2000;62:65-9
49. Price R, van Vugt M, Phaipun L, Luxemburger C, Simpson J, McGready R, ter Kuile F, Kham A, Chongsuphajaisiddhi T, White NJ and Nosten F. Adverse effects in patients with acute falciparum malaria treated with artemisinin derivatives. Am J Trop Med Hyg 1999;60:547-55
50. Touze JE, Heno P, Fourcade L, Deharo JC, Thomas G, Bohan S, Paule P, Riviere P, Kouassi E and Buguet A. The effects of antimalarial drugs on ventricular repolarization. Am J Trop Med Hyg 2002;67:54-60
51. Nosten F, ter Kuile FO, Luxemburger C, Woodrow C, Kyle DE, Chongsuphajaisiddhi T and White NJ. Cardiac effects of antimalarial treatment with halofantrine. Lancet 1993;341:1054-6
52. Brewer TG, Grate SJ, Peggins JO, Weina PJ, Petras JM, Levine BS, Heiffer MH and Schuster BG. Fatal neurotoxicity of arteether and artemether. Am J Trop Med Hyg 1994;51:251-9
53. Brewer TG, Peggins JO, Grate SJ, Petras JM, Levine BS, Weina PJ, Swearengen J, Heiffer MH and Schuster BG. Neurotoxicity in animals due to arteether and artemether. Trans R Soc Trop Med Hyg 1994;88 Suppl 1:S33-6
54. Kamchonwongpaisan S, McKeever P, Hossler P, Ziffer H and Meshnick SR. Artemisinin neurotoxicity: neuropathology in rats and mechanistic studies in vitro. Am J Trop Med Hyg 1997;56:7-12
55. Petras JM, Kyle DE, Gettayacamin M, Young GD, Bauman RA, Webster HK, Corcoran KD, Peggins JO, Vane MA and Brewer TG. Arteether: risks of two-week administration in Macaca mulatta. Am J Trop Med Hyg 1997;56:390-6
56. Nontprasert A, Nosten-Bertrand M, Pukrittayakamee S, Vanijanonta S, Angus BJ and White NJ. Assessment of the neurotoxicity of parenteral artemisinin derivatives in mice. Am J Trop Med Hyg 1998;59:519-22
57. Genovese RF, Newman DB, Petras JM and Brewer TG. Behavioral and neural toxicity of arteether in rats. Pharmacol Biochem Behav 1998;60:449-58
58. Genovese RF, Newman DB, Li Q, Peggins JO and Brewer TG. Dose-dependent brainstem neuropathology following repeated arteether administration in rats. Brain Res Bull 1998;45:199-202
59. Genovese RF, Newman DB, Gordon KA and Brewer TG. Acute high dose arteether toxicity in rats. Neurotoxicology 1999;20:851-9
60. Classen W, Altmann B, Gretener P, Souppart C, Skelton-Stroud P and Krinke G. Differential effects of orally versus parenterally administered qinghaosu derivative artemether in dogs. Exp Toxicol Pathol 1999;51:507-16
61. Li QG, Brueckner RP, Peggins JO, Trotman KM and Brewer TG. Arteether toxicokinetics and pharmacokinetics in rats after 25 mg/kg/day single and multiple doses. Eur J Drug Metab Pharmacokinet 1999;24:213-23

62. Genovese RF, Newman DB and Brewer TG. Behavioral and neural toxicity of the artemisinin antimalarial, arteether, but not artesunate and artelinate, in rats. Pharmacol Biochem Behav 2000;67:37-44
63. Nontprasert A, Pukrittayakamee S, Nosten-Bertrand M, Vanijanonta S and White NJ. Studies of the neurotoxicity of oral artemisinin derivatives in mice. Am J Trop Med Hyg 2000;62:409-12
64. Smith SL, Sadler CJ, Dodd CC, Edwards G, Ward SA, Park BK and McLean WG. The role of glutathione in the neurotoxicity of artemisinin derivatives in vitro. Biochem Pharmacol 2001;61:409-16
65. Wesche DL, DeCoster MA, Tortella FC and Brewer TG. Neurotoxicity of artemisinin analogs in vitro. Antimicrob Agents Chemother 1994;38:1813-9
66. Newton P, Suputtamongkol Y, Teja-Isavadharm P, Pukrittayakamee S, Navaratnam V, Bates I and White N. Antimalarial bioavailability and disposition of artesunate in acute falciparum malaria. Antimicrob Agents Chemother 2000;44:972-7
67. Nontprasert A, Pukrittayakamee S, Dondorp AM, Clemens R, Looareesuwan S and White NJ. Neuropathologic toxicity of artemisinin derivatives in a mouse model. Am J Trop Med Hyg 2002;67:423-9
68. Nontprasert A, Pukrittayakamee S, Prakongpan S, Supanaranond W, Looareesuwan S and White NJ. Assessment of the neurotoxicity of oral dihydroartemisinin in mice. Trans R Soc Trop Med Hyg 2002;96:99-101
69. Ribeiro IR, Olliaro P. Safety of artemisinin and its derivatives. A review of published and unpublished clinical trials. Med Trop (Mars) 1998;58:50-3
70. McIntosh HM, Olliaro P. Artemisinin derivatives for treating uncomplicated malaria. Cochrane Database Syst Rev 2000:CD000256
71. Miller LG, Panosian CB. Ataxia and slurred speech after artesunate treatment for falciparum malaria. N Engl J Med 1997;336:1328
72. Hien TT, Turner GD, Mai NT, Phu NH, Bethell D, Blakemore WF, Cavanagh JB, Dayan A, Medana I, Weller RO, Day NP and White NJ. Neuropathological assessment of artemether-treated severe malaria. Lancet 2003;362:295-6
73. van Agtmael MA, Eggelte TA and van Boxtel CJ. Artemisinin drugs in the treatment of malaria: from medicinal herb to registered medication. 1999;20:199-205
74. McIntosh HM. Chloroquine or amodiaquine combined with sulfadoxine-pyrimethamine for treating uncomplicated malaria. Cochrane Database Syst Rev 2000;2
75. van Vugt M, Looareesuwan S, Wilairatana P, McGready R, Villegas L, Gathmann I, Mull R, Brockman A, White NJ and Nosten F. Artemether-lumefantrine for the treatment of multidrug-resistant falciparum malaria. Trans R Soc Trop Med Hyg 2000;94:545-8
76. van Vugt M, Brockman A, Gemperli B, Luxemburger C, Gathmann I, Royce C, Slight T, Looareesuwan S, White NJ and Nosten F. Randomized comparison of artemether-benflumetol and artesunate-mefloquine in treatment of multidrug-resistant falciparum malaria. Antimicrob Agents Chemother 1998;42:135-9
77. von Seidlein L, Bojang K, Jones P, Jaffar S, Pinder M, Obaro S, Doherty T, Haywood M, Snounou G, Gemperli B, Gathmann I, Royce C, McAdam K and Greenwood B. A randomized controlled trial of artemether/benflumetol, a new antimalarial and pyrimethamine/sulfadoxine in the treatment of uncomplicated falciparum malaria in African children. Am J Trop Med Hyg 1998;58:638-44
78. Hatz C, Abdulla S, Mull R, Schellenberg D, Gathmann I, Kibatala P, Beck HP, Tanner M and Royce C. Efficacy and safety of CGP 56697 (artemether and benflumetol) compared with chloroquine to treat acute falciparum malaria in Tanzanian children aged 1-5 years. Trop Med Int Health 1998;3:498-504
79. Bakyaita N, Dorsey G, Yeka A, Banek K, Staedke SG, Kamya MR, Talisuna A, Kironde F, Nsobya S, Kilian A, Reingold A, Rosenthal PJ, Wabwire-Mangen F. Sulfadoxine-pyrimethamine plus chloroquine or amodiaquine for uncomplicated falciparum malaria: a randomized, multisite trial to guide national policy in Uganda. *Am J Trop Med Hyg* 2005; 72: 573-80
80. Staedke SG, Mpimbaza A, Kamya MR, Nzarubara BK, Dorsey G, Rosenthal PJ. Combination treatments for uncomplicated falciparum malaria in Kampala, Uganda: randomised clinical trial. *Lancet* 2004; 364: 1950-7.

APPENDIX A. INFORMED CONSENT

RESEARCH PARTICIPANT INFORMED CONSENT FORM

**Protocol Title:**	Longitudinal Comparison of Combination Antimalarial Therapies in Ugandan Children: evaluation of safety, tolerability, and efficacy
**Funding Source:**	NIH 1 U01 AI052142
**IND Number:**	69,577
**DMID Protocol Number:**	04-068
**UCSF-CHR Number:**	H2397-25789-05
**Site of Research:**	Mulago Hospital, Kampala Uganda
**Principal Investigator**:	Philip J. Rosenthal, MD
**Date:**	2 January 2008

PURPOSE OF THE STUDY

This research study is being done to learn more about the treatment of malaria. At this time, we would like to know more about the safety of artemether plus lumefantrine (Coartem), which is currently the new first line treatment for malaria in Uganda. To do this, we are carrying out a research study at Mulago Hospital. We will continue to follow a group of about 600 children from Mulago III parish (aged 1-14 years) up to April 2009. Originally, this study compared different drugs for malaria. However, from February 2008 onward, we will not compare treatments. All children with uncomplicated malaria will be treated with artemether plus lumefantrine, which is the new Ugandan standard treatment for malaria.

HOW THE STUDY IS DONE

Over the study period, each time your child has any health problems you are asked to come to the study clinic at the Mulago Assessment Center. Your child will be evaluated there to see if they have malaria. If your child does not have malaria, they will be given appropriate treatment for their illness or will be referred for additional care. If your child does have uncomplicated malaria, they will be treated with artemether plus lumefantrine (Coartem). After each treatment, your child will be followed closely to determine how well they respond to therapy. If your child does not get better after treatment, they will be given quinine to ensure that they get better.

You are being asked to allow your child (or the child under your care, in the case of a legal guardian) to participate in this study until April 30, 2009 or until such a time that you or the study doctors decide that your child should no longer participate in the study. The study may be discontinued by the National Institutes of Health (funding agency) at any time for any reason, and your study doctors may withdraw your child from the study for the following reasons:

1. If you move out of Kampala for more than 60 consecutive days.
2. If we are unable to locate you or your child for more than 60 consecutive days.
3. If you chose to withdraw your consent to participate in this study.
4. If your child develops any serious health problem felt to be probably or definitely related to study treatments.
5. If your child has malaria and fails to be cured by treatment with quinine plus clindamycin, if this treatment is required.
6. If you are unable to comply with the study schedule and procedures outlined below at any time during the course of the study.
7. If your child is diagnosed with a serious chronic disease requiring frequent medical care

PROCEDURES

During study follow-up

a) Each time your child has any health problems, please bring your child to our study clinic. We ask that our study clinic be the only place where your child receives care and medications. There will be someone at the study clinic every day from 8:00 am to 5:00 pm.
b) If your child needs care after 5:00 pm, please bring them to the Acute Care Unit at Mulago Hospital and tell the hospital staff that your child is a part of this study. You may also call our study doctors at any time on our emergency mobile phone (0772-431893).
c) We would like to see your child every 30 days, even if he/she has not been sick. A home visitor may visit you at your home for a routine visit.
d) At least every 3 months, we will also need to take blood from your child (about a teaspoon, from a blood vessel) to examine for malaria parasites, to measure blood counts, and to evaluate the liver. This will happen at the study clinic either during follow-up of a malaria episode, or the home health visitor will visit you at your home to bring you and your child to the study clinic for assessment.

At each study clinic visit for a new illness

a) The study doctors will examine your child.
b) If malaria is not suspected, your child will be treated for the diagnosed condition.
c) If malaria is suspected, a small amount of blood will be taken from your child's finger to examine for malaria parasites.
d) If malaria is not diagnosed, your child will be treated by the study doctor or referred to another part of Mulago Hospital for appropriate care.
e) If malaria is diagnosed, approximately one teaspoon of blood will be taken from your child (from a blood vessel) to count the number of malaria parasites, to measure blood counts, and to evaluate the liver.
f) If malaria is diagnosed, your child will be treated with Coartem at the study clinic. If your child has severe malaria, they will be treated with quinine (not a study drug) and have follow up procedures upon completion of treatment
g) If malaria is diagnosed, you will be asked to bring your child back to the study clinic at least 5 more times over the next 2 weeks for follow-up visits so that we can judge how well your child is responding to the treatment. At each of these follow-up visits, we will examine your child and a small amount of blood will be taken from your child's finger to examine for malaria parasites and to save on filter paper.
h) At the end of two weeks, and on any other day the doctor feels it is necessary (depending on your child's health) your child will have a blood sample taken (from a blood vessel) to examine for malaria parasites, to measure blood counts, and to evaluate the liver. If the treatment given to your child is not working well, the study doctor will change the treatment to quinine.
i) If your child misses an appointment, the home health visitor will visit you at your home to find out why your child missed the appointment and to bring you and your child to the study clinic for assessment.
j) If your child is treated at the Acute Care Unit at Mulago Hospital, please return to our study clinic the next morning.

RISKS AND DISCOMFORTS

Risks involved with the study treatments

1) Minor side effects. The following side effects have been reported in association Coartem; stomach upset, rash, and itching.
2) Severe malaria: Your child may develop malaria that is severe even after receiving treatment with study medications. If your child shows any evidence of severe malaria (including persistent vomiting, low blood counts, convulsions, confusion, or coma) they will be treated with quinine and referred for possible admission to the Acute Care Unit at Mulago Hospital.
3) Unknown Risks: The research treatments may have side effects that no one knows about yet. The researchers will let you know if they learn anything that might make you change your mind about your child's participation in the study.

Risks involved in study procedures

1) Blood draws: The risks of drawing blood from a blood vessel (usually from a vein in the arm or hand) or from a fingerprick include temporary discomfort from the needle stick, bruising, skin infection, and fainting. The amount of blood removed will be too small to affect your child's health.
2) Confidentiality: Participation in research may involve a loss of privacy, but information about your child will be handled as confidentially as possible. Medical information related to malaria will be collected on your child, but only the people working on the study will see it. Anyone assigned to review this study will be granted direct access to your child's medical records, if necessary, for verification of the study procedures and data. Records will be kept as confidential as possible.

BENEFITS

1) Your child will receive clinical care from the doctors, medical officers, and nurses of the project staff in the study clinic.
2) The knowledge gained from this study will help the country of Uganda in determining the best treatment for uncomplicated malaria.

COSTS

After enrolment in the study, you will not be charged for care received at the study clinic. If your child is referred by study physicians to other clinics and services within the Mulago Hospital Complex (including visits to the Acute Care Unit) you will be reimbursed for the cost of consultation. The cost of most diagnostic tests and medications resulting from these visits will also be reimbursed using available funds. However, reimbursement of all diagnostic tests and treatment recommended outside the study clinic cannot be guaranteed in all circumstances.

PAYMENT

You or your child will not be paid for participation in the study. You will be reimbursed for transport costs to and from the study clinic for any visit that your child requires.

ALTERNATIVES TO PARTICIPATION

Your child's participation in this study is completely voluntary. If you decide you do not want your child to participate in the study or decide to withdraw your child from the study at any time and for any reason, this will not affect your child's care at Mulago Hospital, where standard care for all medical problems is available. The Ugandan Ministry of Health currently recommends co-artemether (Coartem) for treatment of uncomplicated malaria, and quinine for treatment of severe malaria. These treatments are generally available at Mulago Hospital.

NEW INFORMATION

During the study, you will be informed promptly of any new information that may influence your willingness to continue participation in the study.

CONSEQUENCES OF WITHDRAWAL

Should you or your study doctors decide to withdraw your child from the study, your child will still be eligible for care at Mulago Hospital, but payment for all treatment and medications will be your responsibility. If your child is withdrawn from the study because a serious health problem develops, which is felt to be at least probably related to the study treatments, or because your child has malaria which is failed to be cured by treatment with quinine and clindamycin, appropriate follow-up tests will be performed. If your child is withdrawn from the study because you are unable to comply with the study schedule and procedures, additional follow-up tests will be performed if the study doctors feel they are necessary, and you agree they should be done.

USE OF THE RESULTS

The findings from this study may be published in a medical journal. The study participants will not be identified by name. After the study is completed, you may request an explanation of the study results.

PRIVACY INFORMATION

We will keep the study information private. Under certain conditions, people responsible for making sure that the research is done properly may review your study records. This might include people from University of California, San Francisco, the National Institutes of Health (U.S.), Food and Drug Authority, and others involved with monitoring the study. All of these people are also required to keep your identity confidential. Otherwise, the information that identifies you will not be given out to people who are not working on the study.

TREATMENT AND COMPENSATION FOR INJURY

If you are injured or have questions about injuries as a result of being in the study, please contact the doctors in the study clinic and/or Dr. Moses Kamya (telephone 0414-541188 or 0414-533200) at) at MU-UCSF Malaria Research Collaboration, behind Department of Anatomy, Makerere Medical School, Mulago Hospital, Kampala. The usual services offered at the study clinic and at Mulago Hospital will be available in case of any such injury. Care will be provided free of charge for injuries related to study participation using available funds.

QUESTIONS

Dr. Kamya and staff are available to explain this study to you and answer your questions. If you have questions about the study, you may call Dr. Kamya at (telephone 0414-541188 or 0414-533200) at MU-UCSF Malaria Research Collaboration, behind Department of Anatomy, Makerere Medical School, Mulago Hospital, Kampala.

You may also contact Dr. Elly Katabira (telephone 0414-530020) at Mulago Hospital to ask questions about your rights as a research subject.

JOINING OF YOUR OWN FREE WILL

PARTICIPATION IN RESEARCH IS VOLUNTARY. You and your child have the right to refuse to participate or to withdraw at any point in this study without penalty or loss of benefits to which you are otherwise entitled.

WHAT YOUR SIGNATURE OR THUMBPRINT MEANS

This consent form will be given to you. Your signature or thumbprint below means that you have had this study explained to you about your child's participation in the study and have had the opportunity to ask questions. If you wish

your child to participate in this study, you should sign or place your thumbprint below. You will also be asked to sign another informed consent forms for the use of stored specimens.

Name of Participant (printed)

Name of Parent/Guardian

Signature or Fingerprint * of Parent/Guardian Date/Time

Name of Investigator Administering Consent (printed) Position/Title

---

Signature of Investigator Administering Consent Date/Time

*If the parent or guardian is unable to read and/or write, an impartial witness should be present during the informed consent discussion. After the written informed consent form is read and explained to the participant and parent or guardian, and after they have orally consented to their child's participation in the trial, and have either signed the consent form or provided their fingerprint, the witness should sign and personally date the consent form. By signing the consent form, the witness attests that the information in the consent form and any other written information was accurately explained to, and apparently understood by the parent or guardian, and that informed consent was freely given by the patient and parent or guardian.

Name of Person Witnessing Consent (printed)

Signature of Person Witnessing Consent Date/Time

INFORMED CONSENT FOR FUTURE USE OF BIOLOGICAL SPECIMENS

**Protocol Title:**	Longitudinal Comparison of Combination Antimalarial Therapies in Ugandan Children: evaluation of safety, tolerability, and efficacy
**Funding Source:**	NIH 1 U01 AI052142
**IND Number:**	69,577
**DMID Protocol Number:**	04-068
**UCSF-CHR Number:**	H2397-25789-01
**Site of Research:**	Mulago Hospital, Kampala Uganda
**Principal Investigator**:	Philip J. Rosenthal, MD
**Date:**	12 October 2004

INTRODUCTION

While your child is in this study, there may be blood samples taken from them that may be useful for future research. These samples will be stored long-term at Makerere University Medical School and the University of California, San Francisco. Samples may also be shared with investigators at other institutions.

WHAT SAMPLES WILL BE USED FOR

Your child's blood will be used to study malaria and the response of this disease to treatment. Results of these studies will not affect your child's care.

1. These samples will be used for future research to learn more about malaria and other diseases.
2. Your child's samples will be used only for research and will not be sold or used for the production of commercial products.
3. Genetic research may be performed on samples. However, no genetic information obtained from this research will be placed in your child's medical records. These samples will be identified only by codes so that they cannot be readily identified with your child.

LEVEL OF IDENTIFICATION

Your child's samples will be coded so that your child's name cannot be readily identified. Reports about research done with your child's samples will not be put in their medical record and will be kept confidential to the best of our ability.

In the future, researchers studying your child's samples may need to know more about your child, such as their age, gender, and race. If this information is already available because of your child's participation in a study, it may be provided to the researcher. Your child's name or anything that might identify them personally will not be provided. You will not be asked to provide additional consent.

RISKS

There are few risks to your child from future use of their samples. A potential risk might be the release of information from your child's health or study records. Reports about research done with your child's samples will not be put in their health record, but will be kept with the study records. The study records will be kept confidential as far as possible.

BENEFITS

There will be no direct benefit to your child. From studying your child's samples we may learn more about malaria or other diseases: how to prevent them, how to treat them, how to cure them.

RESEARCH RESULTS/MEDICAL RECORDS

1. Results from future research using your child's samples may be presented in publications and meetings but patient names will not be identified.
2. Reports from future research done with your child's samples will not be given to you or your child's doctor. These reports will not be put in your child's medical record.

QUESTIONS

Dr. Kamya and staff are available to explain this study to you and answer your questions. If you have any other questions about the information here, you may call Dr. Kamya at (telephone 041-541188 or 041-533200) at Mulago Hospital.

FREEDOM TO REFUSE

You can change your mind at any time about allowing your child's samples to be used for future research. If you do, contact Dr. Moses Kamya (telephone 041-541188 or 041-533200). Then your child's samples will no longer be made available for research and will be destroyed. Whether or not you allow us to use your child's samples in future research will not have any effect on your child's participation in this study or future participation in other studies.

WHAT YOUR SIGNATURE OR THUMBPRINT MEANS

This consent form will be given to you. Your signature or thumbprint below means that it has been explained to you about your child's specimens to be used for future research and have had the opportunity to ask questions. If you wish to allow your child's specimens to be used for future research, you should sign or thumbprint below.

Name of Participant (printed)

Name of Parent/Guardian

Signature or Fingerprint * of Parent/Guardian　　　　Date/Time

Name of Investigator Administering Consent (printed)　　　　Position/Title

---

Signature of Investigator Administering Consent　　　　Date/Time

*If the parent or guardian is unable to read and/or write, an impartial witness should be present during the informed consent discussion. After the written informed consent form is read and explained to the participant and parent or guardian, and after they have orally consented to their child's participation in the trial, and have either signed the consent form or provided their fingerprint, the witness should sign and personally date the consent form. By signing the consent form, the witness attests that the information in the consent form and any other written information was accurately explained to, and apparently understood by, the patient, parent or guardian, and that informed consent was freely given by the patient, parent or guardian.

Name of Person Witnessing Consent (printed)

Signature of Person Witnessing Consent　　　　Date/Time

APPENDIX A2.

INFORMED CONSENT FOR IMMUNOLOGY STUDIES

**Protocol Title:**	Longitudinal Comparison of Combination Antimalarial Therapies in Ugandan Children: evaluation of safety, tolerability, and efficacy
**Funding Source:**	NIH 1 U01 AI052142
**IND Number:**	69,577
**DMID Protocol Number:**	04-068
**UCSF-CHR Number:**	H2397-25789-01
**Site of Research:**	Mulago Hospital, Kampala Uganda
**Principal Investigators**:	Philip J. Rosenthal, MD and Moses Kamya, MBBS, MMed, MPH
**Date:**	27 July 2005

Introduction
Your child is part of our study of treatment for malaria. In order to improve our understanding of your child's response to malaria, we will be performing additional laboratory studies on your child's blood. The results of the studies will be used for research purposes only. They will not affect how we treat your child.

Procedures
Currently in this study, we take blood from your child (about a teaspoon, from a blood vessel) to examine for malaria parasites, to measure blood counts, and to evaluate the liver. Blood is drawn at least every 3 months, and if malaria is diagnosed, at the time of diagnosis and again 2 weeks later. For these additional laboratory studies, each time that blood is drawn from your child, an additional 1 tablespoon of blood will be collected. No extra blood draws will be performed.

Level of Identification
Your child's samples will be coded so that your child's name cannot be readily identified. Reports about research done with your child's samples will not be put in their medical record and will be kept confidential to the best of our ability.

Risks
The additional blood drawn for this study is not expected to affect your child's health. A potential risk might be the release of information from your child's health or study records. The study records will be kept confidential as far as possible.

Benefits
There will be no direct benefit to your child. From studying your child's samples we may learn more about the response to malaria or other diseases: how to prevent them, how to treat them, or how to cure them.

Research results/Medical records
Results from using your child's samples may be presented in publications and meetings but patient names will not be identified. Reports from research done with your child's samples will not be given to you or your child's doctor. These reports will not be put in your child's medical record.

Questions
Dr. Kamya and staff are available to explain this study to you and answer your questions. If you have any questions, you may call Dr. Kamya at (telephone 041-541188 or 041-533200) at Mulago Hospital.
Freedom to Refuse
You can change your mind at any time about allowing your child's samples to be used for this study. If you do, your child's samples will no longer be made available for research and will be destroyed. Whether or not you

allow us to use your child's samples for this study will not affect your child's participation in the study on malaria treatment or future participation in other studies.

What your signature or thumbprint means
This consent form will be given to you. Your signature or thumbprint below means that this study has been explained to you and that you have had the opportunity to ask questions. If you agree to allow your child's specimens to be used for this research, you should sign or thumbprint below.

____

Name of Participant (printed)

____

Name of Parent/Guardian

____

Signature or Fingerprint * of Parent/Guardian Date/Time

____

Name of Investigator Administering Consent (printed) Position/Title

____

Signature of Investigator Administering Consent Date/Time

*If the parent or guardian is unable to read and/or write, an impartial witness should be present during the informed consent discussion. After the written informed consent form is read and explained to the participant and parent or guardian, and after they have orally consented to their child's participation in the trial, and have either signed the consent form or provided their fingerprint, the witness should sign and personally date the consent form. By signing the consent form, the witness attests that the information in the consent form and any other written information was accurately explained to, and apparently understood by, the patient, parent or guardian, and that informed consent was freely given by the patient, parent or guardian.

____

Name of Person Witnessing Consent (printed)

____

Signature of Person Witnessing Consent Date/Time

APPENDIX B1.WEIGHT-BASED ADMINISTRATION OF STUDY MEDICATION FOR PHASE I

AQ+SP: Weight-based administration of study medications

Weight (kg)	Amodiaquine (AQ) 200 mg tabs (Base)			SP 500mg /25mg tabs	Placebo		
C = clinic H = home	Day 0	Day 1	Day 2	Day 0	Day 0	Day 1	Day 2
	C	C	C	C	H	H	H
10	½	½	¼	½	1	1	1
11	½	½	¼	½	1	1	1
12	½	½	½	½	1	1	1
13	½	½	½	¾	1	1	1
14	¾	½	½	¾	1	1	1
15	¾	½	½	¾	2	2	2
16	¾	¾	½	¾	2	2	2
17	¾	¾	½	¾	2	2	2
18	1	¾	½	¾	2	2	2
19	1	¾	½	1	2	2	2
20	1	1	½	1	2	2	2
21	1	1	½	1	2	2	2
22	1 ¼	1	½	1	2	2	2
23	1 ¼	1	½	1 ¼	2	2	2
24	1 ¼	1 ¼	½	1 ¼	2	2	2
25	1 ¼	1 ¼	½	1 ¼	3	3	3
26-27	1 ¼	1	1	1 ½	3	3	3
28	1 ¼	1 ¼	1	1 ½	3	3	3
29-31	1 ¼	1 ¼	1	1 ½	3	3	3
32	1 ½	1 ½	1	1 ½	3	3	3
33	1 ½	1 ½	1	1 ½	3	3	3
34	1 ½	1 ½	1 ¼	1 ½	3	3	3
35	1 ½	1 ½	1 ¼	1 ½	4	4	4
36-37	1 ½	1 ½	1 ¼	1 ½	4	4	4
38	2	1 ½	1 ¼	2	4	4	4
39	2	1 ½	1 ¼	2	4	4	4
40-43	2	2	1	2	4	4	4
44-47	2	2	1 ½	2 ½	4	4	4
48-49	2	2	2	2 ½	4	4	4
50-59	2 ½	2	2	2 ¾	4	4	4
≥60	3	3	1 ½	3	4	4	4

Treatment Group	Day 0		Day 1		Day 2	
	Clinic	Home	Clinic	Home	Clinic	Home
AQ+SP	AQ	Placebo	AQ	Placebo	AQ	Placebo
	SP	-	-	-	-	-

AQ+AS: Weight-based administration of study medications

Weight (kg)	Amodiaquine (AQ) 200 mg tabs (Base)			Artesunate (AS) 50 mg tabs			Placebo tablets		
C = clinic H = home	Day 0 C	Day 1 C	Day 2 C	Day 0 C	Day 1 C	Day 2 C	Day 0 H	Day 1 H	Day 2 H
10	½	½	¼	¾	¾	¾	1	1	1
11	½	½	¼	1	1	1	1	1	1
12	½	½	½	1	1	1	1	1	1
13	½	½	½	1	1	1	1	1	1
14	¾	½	½	1 ¼	1 ¼	1 ¼	1	1	1
15	¾	½	½	1 ¼	1 ¼	1 ¼	2	2	2
16	¾	¾	½	1 ¼	1 ¼	1 ¼	2	2	2
17	¾	¾	½	1 ½	1 ½	1 ½	2	2	2
18	1	¾	½	1 ½	1 ½	1 ½	2	2	2
19	1	¾	½	1 ½	1 ½	1 ½	2	2	2
20	1	1	½	1 ¾	1 ¾	1 ¾	2	2	2
21	1	1	½	1 ¾	1 ¾	1 ¾	2	2	2
22	1 ¼	1	½	1 ¾	1 ¾	1 ¾	2	2	2
23	1 ¼	1	½	2	2	2	2	2	2
24	1 ¼	1 ¼	½	2	2	2	2	2	2
25	1 ¼	1 ¼	½	2	2	2	3	3	3
26-27	1 ¼	1	1	2 ¼	2 ¼	2 ¼	3	3	3
28	1 ¼	1 ¼	1	2 ¼	2 ¼	2 ¼	3	3	3
29-31	1 ¼	1 ¼	1	2 ½	2 ½	2 ½	3	3	3
32	1 ½	1 ½	1	2 ½	2 ½	2 ½	3	3	3
33	1 ½	1 ½	1	2 ¾	2 ¾	2 ¾	3	3	3
34	1 ½	1 ½	1 ¼	2 ¾	2 ¾	2 ¾	3	3	3
35	1 ½	1 ½	1 ¼	2 ¾	2 ¾	2 ¾	4	4	4
36-37	1 ½	1 ½	1 ¼	3	3	3	4	4	4
38	2	1 ½	1 ¼	3	3	3	4	4	4
39	2	1 ½	1 ¼	3 ¼	3 ¼	3 ¼	4	4	4
40-43	2	2	1	3 ¼	3 ¼	3 ¼	4	4	4
44-47	2	2	1 ½	3 ½	3 ½	3 ½	4	4	4
48-49	2	2	2	3 ¾	3 ¾	3 ¾	4	4	4
50-59	2 ½	2	2	4	4	4	4	4	4
≥60	3	3	1 ½	5	5	5	4	4	4

Treatment Groups	Day 0		Day 1		Day 2	
	Clinic	Home	Clinic	Home	Clinic	Home
AQ+AS	AQ	Placebo	AQ	Placebo	AQ	Placebo
	AS	-	AS	-	AS	-

Coartem: Weight-based administration of study medications

**Weight (kg)**	**Coartemether 20mg /120 mg tabs**					
**C = clinic H = home**	**Day 0**		**Day 1**		**Day 2**	
	C	H	C	H	C	H
10	1	1	1	1	1	1
11	1	1	1	1	1	1
12	1	1	1	1	1	1
13	1	1	1	1	1	1
14	1	1	1	1	1	1
15	2	2	2	2	2	2
16	2	2	2	2	2	2
17	2	2	2	2	2	2
18	2	2	2	2	2	2
19	2	2	2	2	2	2
20	2	2	2	2	2	2
21	2	2	2	2	2	2
22	2	2	2	2	2	2
23	2	2	2	2	2	2
24	2	2	2	2	2	2
25	3	3	3	3	3	3
26-27	3	3	3	3	3	3
28	3	3	3	3	3	3
29-31	3	3	3	3	3	3
32	3	3	3	3	3	3
33	3	3	3	3	3	3
34	3	3	3	3	3	3
35	4	4	4	4	4	4
36-37	4	4	4	4	4	4
38	4	4	4	4	4	4
39	4	4	4	4	4	4
40-43	4	4	4	4	4	4
44-47	4	4	4	4	4	4
48-49	4	4	4	4	4	4
50-59	4	4	4	4	4	4
≥60	4	4	4	4	4	4

**Treatment Groups**	**Day 0**		**Day 1**		**Day 2**	
	**Clinic**	**Home**	**Clinic**	**Home**	**Clinic**	**Home**
Coartemether	Coartem	Coartem	Coartem	Coartem	Coartem	Coartem

APPENDIX B2. WEIGHT-BASED ADMINISTRATION OF STUDY MEDICATION FOR PHASE II

AQ+AS: Weight-based administration of study medications (discontinued on February 1, 2008)

Weight (kg)	Amodiaquine (AQ) 200 mg tabs (Base)			Artesunate (AS) 50 mg tabs		
C = clinic H = home	Day 0	Day 1	Day 2	Day 0	Day 1	Day 2
	C	C	C	C	C	C
10	½	½	¼	¾	¾	¾
11	½	½	¼	1	1	1
12	½	½	½	1	1	1
13	½	½	½	1	1	1
14	¾	½	½	1 ¼	1 ¼	1 ¼
15	¾	½	½	1 ¼	1 ¼	1 ¼
16	¾	¾	½	1 ¼	1 ¼	1 ¼
17	¾	¾	½	1 ½	1 ½	1 ½
18	1	¾	½	1 ½	1 ½	1 ½
19	1	¾	½	1 ½	1 ½	1 ½
20	1	1	½	1 ¾	1 ¾	1 ¾
21	1	1	½	1 ¾	1 ¾	1 ¾
22	1 ¼	1	½	1 ¾	1 ¾	1 ¾
23	1 ¼	1	½	2	2	2
24	1 ¼	1 ¼	½	2	2	2
25	1 ¼	1 ¼	½	2	2	2
26-27	1 ¼	1	1	2 ¼	2 ¼	2 ¼
28	1 ¼	1 ¼	1	2 ¼	2 ¼	2 ¼
29-31	1 ¼	1 ¼	1	2 ½	2 ½	2 ½
32	1 ½	1 ½	1	2 ½	2 ½	2 ½
33	1 ½	1 ½	1	2 ¾	2 ¾	2 ¾
34	1 ½	1 ½	1 ¼	2 ¾	2 ¾	2 ¾
35	1 ½	1 ½	1 ¼	2 ¾	2 ¾	2 ¾
36-37	1 ½	1 ½	1 ¼	3	3	3
38	2	1 ½	1 ¼	3	3	3
39	2	1 ½	1 ¼	3 ¼	3 ¼	3 ¼
40-43	2	2	1	3 ¼	3 ¼	3 ¼
44-47	2	2	1 ½	3 ½	3 ½	3 ½
48-49	2	2	2	3 ¾	3 ¾	3 ¾
50-59	2 ½	2	2	4	4	4
≥60	3	3	1 ½	5	5	5

Treatment Group	Day 1	Day 2	Day 3
	Clinic	Clinic	Clinic
AQ+AS	AQ	AQ	AQ
	AS	AS	AS

Coartem: Weight-based administration of study medications

Weight (kg)	Coartemether 20mg /120 mg tabs					
C = clinic H = home	Day 0		Day 1		Day 2	
	C	H	C	H	C	H
10	1	1	1	1	1	1
11	1	1	1	1	1	1
12	1	1	1	1	1	1
13	1	1	1	1	1	1
14	1	1	1	1	1	1
15	2	2	2	2	2	2
16	2	2	2	2	2	2
17	2	2	2	2	2	2
18	2	2	2	2	2	2
19	2	2	2	2	2	2
20	2	2	2	2	2	2
21	2	2	2	2	2	2
22	2	2	2	2	2	2
23	2	2	2	2	2	2
24	2	2	2	2	2	2
25	3	3	3	3	3	3
26-27	3	3	3	3	3	3
28	3	3	3	3	3	3
29-31	3	3	3	3	3	3
32	3	3	3	3	3	3
33	3	3	3	3	3	3
34	3	3	3	3	3	3
35	4	4	4	4	4	4
36-37	4	4	4	4	4	4
38	4	4	4	4	4	4
39	4	4	4	4	4	4
40-43	4	4	4	4	4	4
44-47	4	4	4	4	4	4
48-49	4	4	4	4	4	4
50-59	4	4	4	4	4	4
≥60	4	4	4	4	4	4

Treatment Groups	Day 0		Day 1		Day 2	
	**Clinic**	**Home**	**Clinic**	**Home**	**Clinic**	**Home**
Coartemether	Coartem	Coartem	Coartem	Coartem	Coartem	Coartem

APPENDIX C.1 CLINICAL OUTCOME CLASSIFICATION

**ETF (Early Treatment Failure): Days 0, 1, 2, and 3**<br><br>• Development of danger signs or severe malaria on Days 0-3 in the presence of parasitemia<br>• Parasitemia on Day 2 higher than on Day 0, irrespective of temperature<br>• Parasitemia on Day 3 with temperature ≥ 38.0°C (tympanic)<br>• Parasitemia on Day 3 > 25% of count on Day 0
**LCF (Late Clinical Failure): Days 4 To 14**<br><br>• Development of danger signs or severe malaria after Day 3 in the presence of parasitemia, without previously meeting any of the criteria of early treatment failure<br>• Temperature ≥ 38.0°C (tympanic), or history of fever in past 24 hours, on Days 4 to 14 in the presence of parasitemia, without previously meeting any of the criteria of early treatment failure
**LPF (Late Parasitological Failure): Day 14**<br><br>• Presence of parasitemia on Day 14 and temperature < 38.0°C (tympanic), without previously meeting any of the criteria of early or late treatment failure
**ACPR (Adequate Clinical and Parasitological Response)**<br><br>• Absence of parasitemia on Day 14, irrespective of temperature, without previously meeting any of the criteria of early or late treatment failure

APPENDIX C.2 SEVERE FALCIPARUM MALARIA

A patient with severe falciparum malaria may present with confusion, or drowsiness with extreme weakness (prostration). In addition the following may develop (singly or in combination):

- Cerebral malaria - defined as unarousable coma not attributable to any other cause in a patient with falciparum malaria
- Generalized convulsions
- Severe normocytic anemia
- Hypoglycemia
- Metabolic acidosis with respiratory distress
- Fluid and electrolyte disturbances
- Acute renal failure
- Acute pulmonary edema and adult respiratory distress syndrome (ARDS)
- Circulatory collapse, shock, septicemia ("algid malaria")
- Abnormal bleeding
- Jaundice
- Hemoglobinuria
- High fever
- Hyperparasitemia

APPENDIX D. GRADING GUIDELINES

TABLE I. GUIDELINES FOR GRADING PATIENT SYMPTOMS†

	**Grade 1**<br>**MILD**	**Grade 2**<br>**MODERATE**	**Grade 3**<br>**SEVERE**	**Grade 4**<br>**LIFE THREATENING**
**Subjective fever in the past 24 hours**	N/A	Present (Yes)	N/A	N/A
**Weakness**	Mild decrease in activity; For children – weak, but still playing	Moderate decrease in activity; For children – weak, and playing limited	Not participating in usual activities; For children – not playing	Lethargy, prostration
**Headache***	Mild, "little", on and off; no therapy required	Intermittent and "too much", persistent and dull; therapy required	Persistent and severe; not responding to initial therapy	Intractable; requires repeated narcotic therapy and/or hospitalization
**Anorexia**	Decreased appetite, but still taking solid food	Decreased appetite, avoiding solid food	Refusing to breast feed, appetite very decreased, no solids or liquids taken (< 2 years < 24 hours; ≥ 2 years < 36 hours)	Refusing to breast feed, appetite very decreased, no solids or liquids taken (< 2 years ≥ 24 hours; ≥ 2 years ≥ 36 hours)
**Nausea***	Mild, transient feeling of impending vomiting; maintains reasonable intake	Moderate and/or constant feeling of impending vomiting; intake decreased	Severe, constant feeling of impending emesis; intake decreased significantly	N/A
**Vomiting**	Transient emesis (1-3 episodes/day)	Occasional or moderate vomiting (> 3 episodes/day)	Orthostatic hypotension or IV fluids required	Hypotensive shock or hospitalization required for IV fluid therapy
**Abdominal pain***	Mild, "little", on and off; no therapy required	Intermittent and "too much", persistent and dull; therapy required	Persistent and severe; not responding to initial therapy	Severe – hospitalized for treatment
**Diarrhea**	Transient 2-4 loose stools/day	5-7 loose stools/day	Orthostatic hypotension or > 7 loose stools/day or IV fluids required	Hypotensive shock or hospitalization for IV fluid therapy required
**Cough**	Transient / intermittent	Persistent / constant	Uncontrolled	Cyanosis, stridor, severe shortness of breath
**Pruritis**	Pruritis without rash	Pruritic rash, pruritis without rash that disturbs sleep	Mild urticaria	Severe urticaria, anaphylaxis, angioedema
**“Flu” (viral URI)**	Mild nasal congestion, mild rhinorrhea	Moderate nasal congestion, moderate rhinorrhea	N/A (if severe, classify individual symptoms)	N/A (if life-threatening, classify individual symptoms)
*** Assess only in children ≥ 3 years of age. Answer N/A for younger children and those unable to answer.**				

† Reference – Based on WHO Toxicity Grading Scale for Determining the Severity of Adverse Events

December 2004

TABLE II.
GUIDELINES FOR GRADING PHYSICAL EXAMINATION FINDINGS*

	Grade 1<br>MILD	Grade 2<br>MODERATE	Grade 3<br>SEVERE	Grade 4<br>LIFE-THREATENING
**Temperature (tympanic membrane)**	38.0-38.4°C	38.5-40.0°C	> 40.0°C	Sustained fever, equal or greater than 40.0°C for longer than 5 days
**Temperature (axillary)**	37.5-37.9°C	38.0-39.5°C	> 39.5°C	Sustained fever, equal or greater than 40.0°C for longer than 5 days
**Pallor**	Minimally pale conjunctiva, nail beds	Moderately pale conjunctiva, nail beds	Paper white conjunctiva, nail beds, palms.	N/A
**Eyes**	Redness, conjunctival injection, excessive tearing	Conjunctival discharge	Eye pain, periorbital edema, exophthalmos, blurred vision, miosis, mydriasis, double-vision	Blindness or visual field defects, signs of endophthalmitis, paralysis of extraocular muscles, papilledema
**Ears†**	Edema or hyperemia of pina, canal, or tympanic membrane	Tenderness of pina, red bulging tympanic membrane	Purulent discharge, perforated ear drum	N/A
**Oropharynx**	Hyperemia, pigmentation,	Pharyngeal exudates or erythema	Tonsillar swelling, gum bleeding, blisters, ulceration	Tonsillar obstruction
**Neck**	Non-tender lymphadenopathy, erythema	Tender lymphadenopathy, swelling, tenderness, glandular enlargement	Tracheal deviation	Nuchal rigidity, stridor
**Chest**	Mildly increased RR (for age, temperature), transient or localised adventitious sounds	Moderately increased RR, diffuse or persistent adventitious sounds	Rapid RR (< 2 months > 60, 2-12 months > 50, 1-5 years > 40, adults > 30)* nasal flaring, retractions	Cyanosis
**Cardio-vascular System (CVS)**	Grade 1 murmur	Asymptomatic change in rhythm or extra heart sounds (no treatment required); Grade 2 murmur	Recurrent/persistent change in rhythm or extra heart sounds (treatment required), Grade 3-4 murmur	Change in rhythm or extra heart sounds that require treatment and/or hospitalization; Grade 5-6 murmur
**Abdomen**	Normal bowel sounds, mild localised tenderness, and/or liver palpable 2-4 cm below the right costal margin (RCM), and/or spleen palpable, and/or umbilical hernia present	Normal or mildly abnormal bowel sounds, moderate or diffuse tenderness; and/or mild to moderately enlarged liver (4-6 cm below the RCM) and/or spleen palpable up to half-way between umbilicus and symphysis pubis	Severely abnormal bowel sounds, severe tenderness to palpation. Evidence of peritoneal irritation and/or significant enlargement of liver (> 6 cm below the RCM) and/or spleen palpable beyond half-way between umbilicus and symphysis pubis	Absent bowel sounds. Involuntary rigidity
**Skin**	Localised rash, erythema, or pruritis	Diffuse, maculopapular rash, dry desquamation	Vesiculation, moist desquamation, or ulceration	Exfoliative dermatitis, mucous membrane involvement or erythema multiforme or suspected Stevens-Johnson or necrosis requiring surgery
**† If both ear canals blocked by cerumen, record "N/A"**				

December 2004

TABLE II.
GUIDELINES FOR GRADING PHYSICAL EXAMINATION FINDINGS (cont.)

	**Grade 1 MILD**	**Grade 2 MODERATE**	**Grade 3 SEVERE**	**Grade 4 LIFE-THREATENING**
**Hearing**	*< 4 years: N/A*<br>≥ 4 years: Decreased hearing in one ear	*< 4 years: N/A*<br>≥ 4 years: Decreased hearing in both ears or severe impairment in one ear	*< 4 years: Any evidence of hearing impairment*<br>≥ 4 years: Severe impairment in both ears	N/A
**Tablet test**	Difficulty grasping tablet but able to pick up	Unable to pick up tablet without dropping	Unable to grasp tablet	N/A
**Clinical symptoms or signs that may be listed as "other"**				
**Behavioural changes**	Mild difficulty concentrating; mild confusion or agitation; activities of daily living unaffected; no treatment	Moderate confusion or agitation; some limitation of activities of daily living; minimal treatment	Severe confusion or agitation; Needs assistance for activities of daily living; therapy required	Toxic psychosis; hospitalization required
**Convulsion**	N/A	N/A	Localized or generalized seizure	Status epilepticus
**Dehydration**	Less than 2 of the following:<br>• Restless, irritable<br>• Sunken eyes<br>• Drinks eagerly, thirsty<br>• Skin pinch goes back slowly	2 of the following:<br>• Restless, irritable<br>• Sunken eyes<br>• Drinks eagerly, thirsty<br>• Skin pinch goes back slowly	Two of the following:<br>• Lethargic or unconscious<br>• Sunken eyes<br>• Not able to drink or drinking poorly<br>• Skin pinch goes back very poorly	Two of the following + shock:<br>• Lethargic or unconscious<br>• Sunken eyes<br>• Not able to drink or drinking poorly<br>• Skin pinch goes back very poorly
**Muscle and/or joint aches**	Mild localised complaints	Mild diffuse complaints	Objective weakness; function limited	N/A
**Tinnitus**	Mild ringing or roaring sound	Moderate ringing or roaring sound	Severe ringing or roaring sound with associated hearing loss	N/A
**Jaundice**	Slight yellowing of sclera and conjunctiva	Moderate yellowing of sclera and conjunctiva, yellowing of mucous membranes	Severe yellowing of sclera and conjunctiva, yellowing of skin	N/A
**Clinical symptoms / sign** *(not otherwise specified)*	No therapy; monitor condition	May require minimal intervention and monitoring	Requires medical care and possible hospitalization	Requires active medical intervention, hospitalization, or hospice care

*** References - DMID Pediatric Toxicity Tables, May 2001, The Harriet Lane Handbook, 15th edition, 2000, WHO Toxicity Grading Scale for Determining the Severity of Adverse Events**

December 2004

TABLE III.
GUIDELINES FOR GRADING LABORATORY RESULTS

	Grade 1<br>MILD	Grade 2<br>MODERATE	Grade 3<br>SEVERE	Grade 4<br>LIFE-THREATENING
**Absolute neutrophil count*** *(/mm^3)*	750-1200	400-749	250-399	< 250
**Hemoglobin** *(g/dL)*	9.0 – 9.9	7.0 – 8.9	5.0 – 6.9	< 5.0
**Platelets** *(/mm^3)**	N/A	50,000-75,000	25,000-49,999	< 25,000
**ALT** *(U/L)***	1.1-4.9 x ULN<br>(50 – 224)	5.0-9.9 x ULN<br>(225 – 449)	10.0-15.0 x ULN<br>(450 – 675)	> 15.0 x ULN<br>(> 675)
**Bilirubin** *(mg/dL)**	1.1-1.9 x ULN<br>(1.3 - 2.3)	2.0-2.9 x ULN<br>(2.4 - 3.5)	3.0-7.5 x ULN<br>(3.6 - 9.0)	> 7.5 x ULN<br>(> 9.0)
**Creatinine** *(mg/dl))**<br>**Age < 2 years**	0.6-0.8	0.9-1.1	1.2-1.5	> 1.5
**Creatinine** *(mg/dl))**<br>**Age ≥ 2 years**	0.7-1.0	1.1-1.6	1.7-2.0	> 2.0
**Laboratory values** *(not otherwise specified)*	Abnormal but requiring no immediate intervention; follow	Sufficiently abnormal to require evaluation as to causality and perhaps mild therapeutic intervention	Sufficiently severe to require evaluation and treatment	Life-threatening severity; requires immediate evaluation, treatment, and usually hospitalization

***Reference – DMID Pediatric Toxicity Tables, May 2001**
**** Reference – DAIDS Pediatric guidelines**

December 2004

APPENDIX E. GUIDELINES FOR CLASSIFYING EXPECTED* ADVERSE EVENTS

	Associated with malaria		Event reported in package insert			
**EVENT**	**Yes**	**No**	**AQ**	**AS**	**AL**	**SP**
**SYMPTOMS**						
Fever	√					√
Weakness (lethargy/tiredness)	√		√		√	√
Muscle/joint aches	√				√	
Headache	√				√	√
Anorexia	√				√	
Nausea	√		√		√	√
Vomiting	√		√		√	√
Abdominal pain	√				√	
Diarrhea	√		√		√	√
Cough		√			√	√
Pruritis		√			√	√
Tinnitus		√				
"Flu"		√				
**EXAM FINDINGS**						
Temperature *(ºC)*	√					√
Palor	√					
Jaundice	√					
Chest	√ (↑ RR)	√				
Abdomen	√					
Skin – see below		√				
Hearing		√				
Tablet test		√				
**LABS**						
Neutrophil decrease (Neutropenia)		√	√			√
Hemoglobin decrease (Anemia)	√		√			√
Platelet count decrease (Thrombocytopenia)	√					√
ALT elevation (Elevation of LFTs)	√ (1/2)	√ (3/4)	√	√	√	√
**OTHER events**						
Convulsion	√					√
Dizziness		√			√	√
Oral lesions/stomatitis		√				√
**OTHER events reported in package inserts**						
**GENERAL**						
Serum sickness		√				√
Hypersensitivity reaction		√			√	
**SKIN**						
Skin rash/drug rash		√			√	√
Urticaria		√				√
Erythema mutiforme		√				√
Stevens-Johnson		√				√
Lyell's syndrome		√				√
Photosensitization		√				√
Mild hair loss		√				√
Purpura		√				√
Bluish-gray pigmentation of fingernails/skin/hard palate		√	√			
**GASTROINTESTINAL**						
Feeling of fullness		√				√
Hepatitis		√	√			√
Liver cell damage		√				√
**LAB**						
Leukopenia		√				√

	Associated with malaria		Event reported in package insert			
**EVENT**	**Yes**	**No**	**AQ**	**AS**	**AL**	**SP**
Agranulocytosis		√	√			√
“Other blood dyscrasias”		√	√			
Megaloblastic anemia		√				√
Hemolytic anemia	√		√			
Reticulocyte count decrease		√		√		
**NEUROLOGIC**						
Sleep disturbance		√			√	
Insomnia		√			√	
Polyneuritis		√				√
Peripheral neuropathy		√	√			
Personality disorder		√			√	
Involuntary muscle contractions		√			√	
Paresthesia		√			√	
Hypoasthesia		√			√	
Abnormal gait		√			√	
Ataxia		√			√	
**PULMONARY**						
Pulmonary infiltrates Infiltrates “such as occur in eosinophilic or allergic alveolitis”		√				√
Dyspnea	√					√
**CARDIOVASCULAR**						
Allergic pericarditis		√				√
Asymptomatic QT prolongation		√			√	
Palpitations	√				√	
**OPHTHO**						
Corneal deposits		√	√			
Visual disturbance		√	√			
Retinopathy (irreversible)		√	√			
**OVERDOSE**					Monitor EKG and potassium	Headache Anorexia Nausea Vomiting Agitation Convulsion Heme changes

* **DMID Expected:** *Is this event an elevation of ALT?*

* **CHR Expected:** *Could this event be expected to occur in association with malaria, or is it reported on ANY package insert?*

APPENDIX F. HOME VISITOR SCRIPT FOR INITIAL CONTACT METHOD FOR STUDY RECRUITMENT

Phase I (October 2004 – April 2005)

Once home visitors have identified the person primarily responsible for health care decision making of selected households with at least one child between the ages of 1 to 10 years of age they will follow the script outlined below to determine if a formal screening appointment can be scheduled in our study clinic (see section 9.3.1 "Initial contact method- Phase I"):

"Your household has been selected for possibly participation in a study to compare different treatments for malaria in children between the ages of 1 and 10 years over the next 3 years. We would like to invite you to come to our study clinic and hear about the full details of the study from one of our study physicians. During the interview in our study clinic we will determine whether any of your children are eligible for participation in our study and whether you might be interested in participating. We will also be able to answer any questions you may have about the study. At this time we would like to schedule an appointment for you to come to our study clinic at Mulago Hospital. Transportation will be provided for you and your children to travel to the study clinic and back to your home."

Phase II (May 2006 - completion)

Home visitors will approach each of the households currently enrolled in the study and will follow the script outlined below to explain Phase II changes and to determine if a formal screening appointment can be scheduled in our study clinic (see section 9.3.1.1 "Initial contact method – Phase II"):

"Your household is currently participating in a study comparing different treatments for malaria in children from Mulago Hospital and we would like to inform you of some of the changes that we have made to this study. First, I would like to ask you a few questions regarding bed nets". [Complete bed net questions on Phase II Initiation form]

"We would now like to give one insecticide treated bed net to each one of the children enrolled in the study and instruct you on how to set up the net and how to care for it. This net is treated to be long-lasting so no further chemical treatment will need to be done for it."
[Complete demonstration of setting up net and record date on the Phase II Initiation form]

"Finally, we would like to ask you about other children in your household who are not currently and who have never been enrolled in the study who are between the ages of 1 and 10 years. Can you tell me how many children you are the guardian for that will be staying in the Mulago III parish for the next two years?"
[Record the additional children section of the Phase II Initiation form]

"We would like to invite you to come with these children to our study clinic and hear about the full details of the study from one of our study physicians. During the interview in our study clinic we will determine whether any of these children are eligible for participation in our study. We will also be able to answer any questions you may have about the study. At this time we would like to schedule an appointment for you to come to our study clinic at Mulago Hospital. Transportation can be arranged for you and the children who will be screened, to travel to the study clinic and back to your home."

APPENDIX G: HOUSEHOLD SURVEY (1)

**Household ID:**<br>**HH-** |___|___|___|___|	**Date of visit:** |___|___| / |___|___| / |___|___| *day month year*

PRIMARY CAREGIVER

1. *"How old are you?"*

|____|____| years

2. *"What is your tribe?"*

□Baganda □ Banyankole □ Banyole
□ Batoro □ Bagwere □ Bakiga
□ Bagisu □ Banyoro □ Lugbara
□ Batesu □ Basoga □ Acholi
□ Other or Mixed □ Bafumbira □ Banyorwanda
____________________________ □ Unknown

3. *"What is your religion?"*

□ Catholic □ Protestant
□ Muslim □ Saved/Born again
□ Seventh day adventist
□ Other (or more than one) ______________________

4. *"What is the highest level of school you completed?"*

□ None □ S 1-2
□ P 1-2 □ S 3-4
□ P 3-4 □ S 5-6
□ P 5-7 □ University
□ Other ______________________

5. *"Have you completed any other type of job training school?"*

□ Yes
□ No

6. *"Now I would like you to read this sentence to me. In what language do you read?"*

SHOW THE APPROPRIATE LITERACY CARD TO PRIMARY CAREGIVER

□ Cannot read at all *If cannot read, skip to question #8*
□ Able to read only parts of sentence
□ Able to read whole sentence
□ No card with required language
(specify language) __________________

7. *"Do you read a newspaper or magazine almost every day, at least once a week, less than once a week or not at all?"*

□ Almost every day
□ At least once a week
□ Less than once a week
□ Not at all

8. *"What is the main activity or job you do to provide your family with income OR "If you do not have regular employment, what other activities do you do to provide your family with income?"*

□ Teacher □ Trucker/driver/conductor
□ Student □ Government/clerical/secretarial
□ Military/police/security □ Mechanic
□ Construction worker □ Heath care worker
□ Housekeeper □ None/Unemployed#
□ Selling goods* □ Other ________________________

**If selling goods is the main job, go to question #9, otherwise, skip to question #10*
# If not currently employed, skip to question #11

9. *"Where are the goods sold?"*

□ From established shop
□ From a market stall or kiosk
□ From a mobile street vendor
□ From the home
□ Other ________________________

10. *How long have you done this main activity or job?"*

□ < 1 months □ 1-5 years
□ 1-5 months □ >5 years
□ 6-11 months □ Unemployed

11. *"Do you normally have work throughout the year, seasonally or only once in awhile?"*

□ Throughout the year
□ Seasonal/Part of the year
□ Once in a while
□ Not at all

12a. *"Have you lived in Kampala your entire life?*

□Yes *If yes, skip to question #13*
□ No

12b. *"How long have you lived in Kampala?"*

□ < 6 months □ 5-10 years
□ 6-11 months □ > 10 years
□ 1- 4 years □ Unknown

12c. *"Before living in Kampala, did you live in a city, in a town, or in the countryside?"*

□ City
□ Town
□ Village or Countryside

13. *IS THE PRIMARY CAREGIVER FEMALE?* □Yes *If yes, go to question #14*
□ No *If no, skip to question #27*

HOUSEHOLD SURVEY (2)

**Household ID:** **HH-** |___|___|___|___|	**Date of visit:** |___|___| / |___|___| / |___|___| *day month year*

WOMEN'S HEALTH QUESTIONS

"Now I want to ask a few questions about your health history. Some of these questions are about sensitive and personal issues. You do not have to answer any questions that make you feel uncomfortable, but any information you give will be very helpful."

14. *"Are you currently pregnant?"*
- □ Yes *If yes, go to question #15*
- □ No *If no, skip to question #20*

15. How many months pregnant are you?

|___|___| Completed Months

16. *"Do you sleep under a bednet?"*
- □ Yes
- □ No

17. *"Did you sleep under a bednet last night?"*
- □ Yes
- □ No

18. *"Now let's talk about the net you sleep under most often. Has the net been soaked or dipped in a liquid to repel mosquitoes or bugs?"*
- □ Yes - Perma net label
- □ Yes - Smart net label
- □ Yes - KO Net
- □ Yes - by report
- □ No *If no, skip to question #23*
- □ Unknown

19. *"How long ago was the net last treated with insecticide?"*
- □ <6months
- □ >6months
- □ Never
- □ Unknown

→ Skip to question #23

20. *"Have you ever been pregnant?"*
- □ Yes *If yes, go to question #21*
- □ No *If no, skip to question #26*

21. *"During your last pregnancy, did you sleep under a bednet?"*
- □ Yes
- □ No *If no, skip to question #23*

22. *"Now let's talk about the net you slept under most often. Was the net soaked or dipped in a liquid to repel mosquitoes or bugs?"*
- □ Yes - Perma net label
- □ Yes - Smart net label
- □ Yes - KO Net
- □ Yes - by report
- □ No
- □ Unknown

23. *"During your last or current pregnancy, did you take any drugs to prevent you from getting malaria?"*
- □ Yes
- □ No *If no, skip to question # 26*
- □ Unknown

24. *"Which drugs did you take to prevent malaria?"*
- □ Chloroquine
- □ Fansidar (SP)
- □ Unknown
- □Other__________________

25. *"How many times did you take these drugs?"*
- □ Once
- □ Twice
- □ Three or more times
- □ Unknown

"Now I would like to ask you about all the births you have had during your life."

26. *"Have you ever given birth to a live child?"*
- □ Yes
- □ No *If no, go to question #34*

"For EACH child YOU have given birth to, what is his/her…"

	Date of birth	*Gender*	*Is the child alive now?*	*If the child has died, HOW OLD was the child when s(he) died? (IF <1 MONTH AT DEATH, ENTER 0 YEARS, 0 MONTH)*
27. Child 1	Month |___|___| Year |___|___|	□ Male □ Female	□ Yes □ No	|___|___| Months |___|___| Years
28. Child 2	Month |___|___| Year |___|___|	□ Male □ Female	□ Yes □ No	|___|___| Months |___|___| Years
29. Child 3	Month |___|___| Year |___|___|	□ Male □ Female	□ Yes □ No	|___|___| Months |___|___| Years
30. Child 4	Month |___|___| Year |___|___|	□ Male □ Female	□ Yes □ No	|___|___| Months |___|___| Years
31. Child 5	Month |___|___| Year |___|___|	□ Male □ Female	□ Yes □ No	|___|___| Months |___|___| Years
32. Child 6	Month |___|___| Year |___|___|	□ Male □ Female	□ Yes □ No	|___|___| Months |___|___| Years
33. Child 7	Month |___|___| Year |___|___|	□ Male □ Female	□ Yes □ No	|___|___| Months |___|___| Years

If >7 children born to the primary caregiver, please continue on HOUSEHOLD SURVEY form (11)

Key for Child Births and Deaths

IF PRIMARY CAREGIVER STATES…	*ENTER….*
Beginning of the year	Month: **2** for February
Middle of the year	Month: **6** for June
End of year	Month: **11** for November

HOUSEHOLD SURVEY (3)

Household ID:

HH- |___|___|___|___|

Date of visit: |___|___| / |___|___| / |___|___|
day month year

HEAD OF HOUSEHOLD

"Now I would like to ask you some questions about your household."

34. *"Who is currently the head of this household?" (Define by relationship to the primary caregiver)*
□ Husband □ Father □ Brother □ Uncle □ Self If self, skip to question #47
□ Wife □ Mother □ Sister □ Aunt □ Other ___________________

35. WHAT IS THE GENDER OF THE HEAD OF THE HOUSEHOLD?
□ Male
□ Female

36. *"How old is the head of household?"*

|___|___| years

37. *"What is the head of household's tribe?*
□ Baganda □ Acholi
□ Banyankole □ Banyole
□ Batoro □ Bagwere
□ Bakiga □ Bagisu
□ Banyoro □ Lugbara
□ Batesu □ Basoga
□ Other or mixed □ Unknown
_________________ □ Munyorwanda

38. *"What is the head of household's religion?"*
□ Catholic □ Protestant
□ Muslim □ Saved/Born again
□ Seventh day adventist
□ Unknown
□ Other (or more than one)
____

39. *"What is the highest level of school completed by the head of household?"*
□ None □ S 1-2
□ P 1-2 □ S 3-4
□ P 3-4 □ S 5-6
□ P 5-7 □ University
□ Unknown
□ Other _______________________

40. *"Has the head of household completed any other type of job training school?"*
□ Yes
□ No
□ Unknown

41. *"In which of the following languages can the head of household read?"*
□ None
□ Luganda / Swahili / Tribal Dialect
□ English
□ Unknown
□ Other ______________________
(tick highest literacy level)

42. *"What is the main activity or job the head of the household does to provide your family with income?" OR "If he/she does not have regular employment, what other activities does he/she do to provide your family with income?"*
□ Teacher □ Trucker/driver/conductor
□ Student □ Government/clerical/secretarial
□ Military/police/security □ Mechanic
□ Construction worker □ Heath care worker
□ Housekeeper □ None/Unemployed #
□ Selling goods* □ Other _______________________

**If selling goods is the main job, go to question #43, otherwise skip to question #44*
# If not currently employed, skip to question #45

43. *"Where are the goods sold?"*
□ From established shop
□ From a market stall or kiosk
□ From a mobile street vendor
□ From the home
□ Other _______________________

44. *"How long has he/she done this main activity or job?" OR "If unemployed, how long since he/she last did an activity or job to provide your family with income?"*
□ < 1 months □ 1-5 years
□ 1-5 months □ >5 years
□ 6-11 months □ Unknown

45. *"Does he/she normally have work throughout the year, seasonally or only once in awhile?"*
□ Throughout the year
□ Seasonal/Part of the year
□ Once in a while
□ Not at all

46a. *"Has the head of household lived in Kampala his/her entire life?*
□Yes *If yes, skip to question #47*
□ No
□ Unknown

46b. *"How long has the head of household lived in Kampala?"*
□ < 6 months □ 5-10 years
□ 6-11 months □ > 10 years
□ 1- 4 years □ Unknown

46c. *"Before living in Kampala, did the head of household live in a city, in a town, or in the village?"*
□ City □ Village
□ Town □ Unknown

HOUSEHOLD INFORMATION

47. *"How many residents currently live in this household?"*
|___|___|

48. *"How many children in this household are directly under your care?"*
|___|___|

49. *"Does this household have any bednets that can be used while sleeping?"*
□Yes
□ No *If no, skip to question #52*

50. *"Have ANY of the nets been soaked or dipped in a liquid to repel mosquitoes or bugs?"*
□ Yes - Perma net label
□ Yes - Smart net label
□ Yes - KO Net
□ Yes - by report
□ No *If no, skip to question #52*
□ Unknown

51. *"How long ago was the net (or nets) last treated?*
□ <6months
□ >6months
□ Never
□ Unknown

52. *"Besides bednets, what do you do to prevent malaria in this household?" (tick all that apply)*
□ Nothing
□ Use sprays, coils, or burnings
□ Use mesh screens in house
□ Close windows/doors in evening
□ Empty/cover water sources
□ Other ______________________

HOUSEHOLD SURVEY (4)

Household ID:

HH- |___|___|___|___|

Date of visit: |___|___| / |___|___| / |___|___|
day month year

HOUSEHOLD ASSETS

53. *"Does the household or a member of the household own this home?"*

□ Own home
□ Rent home

____________________ **Ush per month**

□ Use without paying rent
□ No answer ↓

54. *"How many separate rooms are in the home?"*

|___|___| (# of rooms) ↓

55. *"What is the floor of the home made of?"*
□ Cement
□ Mud/dirt with covering
□ Mud/dirt
□ Other_______________________

56. *"What are the walls made of?"*
□ Mud/dirt (wattle)
□ Mud/clay bricks
□ Cement
□ Other_______________________

57. *"What is the roof made of?"*
□ Metal
□ Grass/thatch
□ Other_______________________

58. *"What kind of toilet facility does your household use?"*

□ None
□ Pan/bucket
□ Uncovered pit latrine
□ Covered pit latrine
□ VIP pit latrine
□ Flush to septic tank
□ Other_________________________

59. "*Is this toilet facility shared with other households?*

□ Yes
□ No

60. *"Does the household have electricity?"*

□ Yes
□ No

61. *"How do you compare the overall economic situation of the household with one year ago?"*

□ Better now
□ Same
□ Worse now
□ Don't know

62. *"Do the members of the household own any land?"*
□ Yes, in Kampala
□ Yes, up-country
□ Unknown
□ No *If no, skip to question #65*

63. *"Who owns the land?" (define by relationship to the primary caregiver)*
□ Mother □ Father
□ Aunt □ Uncle
□ Sister □ Brother
□ Wife □ Husband
□ Self
□ Other _______________________

64. *"How does the amount of land owned compare with one year ago?"*
□ Less now
□ Same
□ More now
□ Don't know

65. *"Do members of the household own cattle or other large livestock (either in Kampala or up-country)?"*

□ Yes
□ No *If no, skip to question #67*
□ Don't know

66. *"How does the number of large livestock owned now compare to one year ago?"*
□ Less now
□ Same
□ More now
□ Don't know

67. *"Do members of the household own any of the following items?" (Tick all that apply, including only those in working condition.)*
□ Iron
□ Watch or clock
□ Bed +/or factory-made mattress
□ Bicycle
□ Radio
□ Television
□ Sofa set
□ Refrigerator
□ Telephone / Mobile phone
□ Motorcycle
□ None of these items
□ Refused to answer

68. *"What is the main fuel used for cooking?"*
□ Charcoal
□ Firewood
□ Other______________________

69. *"Where is the cooking done?"*
□ Food cooked outside or in house NOT in kitchen
□ Kitchen (separate or attached)
□ Other______________________

70. *"Where do you get water?" (tick the primary water source)*
□ Tap in the house
□ Communal tap (closed)
□ Well
□ Protected spring (open)
□ Other _____________________

71. *"How far is your nearest source of water?"*
□ Inside house
□ Distance estimated in meters

|___|___| (meters)

72. "*Do you collect water and store water in or around your house?"*
□ Yes
□ No *If no, skip to question #74*

73. *"How is the water stored?"*
□ Jerry cans
□ Drums
□ Other_____________________

HOUSEHOLD SURVEY (5)

Household ID:

HH- |___|___|___|___|

Date of visit: |___|___| / |___|___| / |___|___|
day *month* *year*

TREATMENT SEEKING BEHAVIOR

"Now I would like to ask you about your experiences with illness in this household and about what you do when your child is sick."

74. *"Is malaria a problem in this household?"*

□ Yes
□ No *If no, skip to question # 76*
□ Unknown

→

75. *"Why do you think malaria is a problem?" (Tick all that apply)*

□ Adults frequently fall sick
□ Children frequently fall sick
□ Children suffer severe illness
□ Children have died
□ Expensive to prevent
□ Expensive to treat
□ Many mosquitoes
□ Unknown
□ Time lost from work/school due to illness or caring for sick child
□ Other ____

76. *"Has any child under your care ever had to stay overnight in a hospital?"*

□ Yes
□ No
□ Unknown

77. *"Has any child under your care ever died?"*

□ Yes
□ No
□ Unknown

78. *"Before your child was enrolled in the study, if your child had a fever, what medicines would you give or buy FIRST?"*

□ Panadol
□ Aspirin
□ Chloroquine (CQ)
□ Septrin
□ Fansidar (SP)
□ Amoxacillin
□ CQ+SP
□ Amodiaquine
□ Quinine
□ None
□ Go to clinic or hospital
□ Don't know
□ Other or more than one:
____

79. *" If your child does not improve with the first treatment, what medicines would you give or buy NEXT?"*

□ Panadol
□ Aspirin
□ Chloroquine (CQ)
□ Septrin
□ Fansidar (SP)
□ Amoxacillin
□ CQ+SP
□ Amodiaquine
□ Quinine
□ None/Go to clinic or hospital
□ Don't know
□ Other or more than one:
____

80. *"What treatment do you think that the Ministry of Health recommends as first-line (standard) therapy for malaria?"*

□ Panadol
□ Aspirin
□ Chloroquine (CQ)
□ Amodiaquine
□ Fansidar (SP)
□ Coartem
□ CQ+SP
□ Quinine
□ Don't know
□ Other_________________________

SATISFACTION WITH TREATMENT

81. *"Have you ever taken your child to a clinic or hospital?"*

□ Yes
□ No *If no, go to question #83*
□ Have not visited clinic or hospital
If not visited, go to question #83

82. *Have you experienced any problems obtaining treatment at a clinic or hospital? (Tick all that apply)*

□ None
□ Facilities not clean
□ Long wait time
□ No trained professionals
□ No drugs available
□ Too expensive
□ Treatment was unsuccessful
□ Provider was rude or unprofessional
□ Other _____________________

83. *"Have you ever gone to a drug shop or pharmacy to obtain treatment for your child?"*

□ Yes
□ No *If no, go to question #85*
□ Have not visited drug shop or pharmacy
If not visited, go to question #85

84. *"Have you experienced any problems obtaining treatment at a drug shop or pharmacy?" (Tick all that apply)*

□ None
□ Facilities not clean
□ Long wait time
□ No trained professionals
□ No drugs available
□ Too expensive
□ Treatment was unsuccessful
□ Provider was rude or unprofessional
□ Other _____________________

85. *"Do you think that herbs are helpful in treating fever or illness?"*

□ Yes
□ No
□ Unknown

86. *"Have you experienced any problems obtaining herbs or visiting a traditional healer?" (Tick all that apply)*

□ None
□ Facilities not clean
□ Long wait time
□ No trained professionals
□ No drugs available
□ Too expensive
□ Treatment was unsuccessful
□ Provider was rude or unprofessional
□ Never tried herbs or visited a traditional healer
□ Other _____________________

HOUSEHOLD SURVEY (6)

Household ID:

HH- |___|___|___|___|

Date of visit: |___|___| / |___|___| / |___|___|
day month year

EXPERIENCE WITH FEVER DURING THE PAST 2 WEEKS

87. *"Did any of the children under your care have fever in the 2 weeks before you came to clinic for the initial screening visit?"*
□ Yes
□ No *If no or unknown, skip to HOUSEHOLD SURVEY form (8)*
□ Unknown

FIRST ACTION

"Now we would like to get a detailed step by step description of everything you did to care for your child during this recent illness. Again, there is no right or wrong answer to these questions. We want you to be as open and honest as possible."

88. *"What did you do FIRST?" (choose one answer)*
□ Nothing
□ Tepid sponging
□ Gave herbs kept at home
□ Gave medicines kept at home
□ Other
*(specify):*____
□ Bought medicines from shop
□ Took child to traditional healer
□ Took child to clinic or hospital
If clinic or hospital, go to question #89

89. *"If you took your child to clinic or hospital, where did you go?"*
□ Private clinic
□ Mulago specialty clinic
□ Private hospital
□ Mulago hospital
□ Other________________

MEDICINES GIVEN AS FIRST TREATMENT

"If your child took medicine FIRST, what did he/she take?

Drug	Number of times given per day	Number of days given	How long had the child been ill when the drug was started?
90. **Panadol**			□ < 24 hrs □ 1-3 d □ 4-7 d □ > 7 d □ Unknown
91. **Chloroquine**			□ < 24 hrs □ 1-3 d □ 4-7 d □ > 7 d □ Unknown
92. **Fansidar** *(SP)*			□ < 24 hrs □ 1-3 d □ 4-7 d □ > 7 d □ Unknown
93. **Amodiaquine**			□ < 24 hrs □ 1-3 d □ 4-7 d □ > 7 d □ Unknown
94. **Quinine**			□ < 24 hrs □ 1-3 d □ 4-7 d □ > 7 d □ Unknown
95. **Septrin** *(Bactrim)*			□ < 24 hrs □ 1-3 d □ 4-7 d □ > 7 d □ Unknown
96. **Amoxacillin**			□ < 24 hrs □ 1-3 d □ 4-7 d □ > 7 d □ Unknown
97. **Other:**__________			□ < 24 hrs □ 1-3 d □ 4-7 d □ > 7 d □ Unknown
98. **Other:**__________			□ < 24 hrs □ 1-3 d □ 4-7 d □ > 7 d □ Unknown

IF ILLNESS RESOLVED, skip to question #121

SECOND ACTION

99. *"What did you do SECOND?" (choose one answer)*
□ Nothing
□ Tepid sponging
□ Gave herbs kept at home
□ Gave medicines kept at home
□ Other
*(specify):*____
□ Bought medicines from shop
□ Took child to traditional healer
□ Took child to clinic or hospital
If clinic or hospital, go to question #100

100. *"If you took your child to clinic or hospital, where did you go?"*
□ Private clinic
□ Mulago specialty clinic
□ Private hospital
□ Mulago hospital
□ Other________________

MEDICINES GIVEN AS SECOND TREATMENT

"If your child took medicine SECOND, what did he/she take?

Drug	Number of times given per day	Number of days given	How long had the child been ill when the drug was started?
101. **Panadol**			□ < 24 hrs □ 1-3 d □ 4-7 d □ > 7 d □ Unknown
102. **Chloroquine**			□ < 24 hrs □ 1-3 d □ 4-7 d □ > 7 d □ Unknown
103. **Fansidar** *(SP)*			□ < 24 hrs □ 1-3 d □ 4-7 d □ > 7 d □ Unknown
104. **Amodiaquine**			□ < 24 hrs □ 1-3 d □ 4-7 d □ > 7 d □ Unknown
105. **Quinine**			□ < 24 hrs □ 1-3 d □ 4-7 d □ > 7 d □ Unknown
106. **Septrin** *(Bactrim)*			□ < 24 hrs □ 1-3 d □ 4-7 d □ > 7 d □ Unknown
107. **Amoxacillin**			□ < 24 hrs □ 1-3 d □ 4-7 d □ > 7 d □ Unknown
108. **Other:**__________			□ < 24 hrs □ 1-3 d □ 4-7 d □ > 7 d □ Unknown
109. **Other:**__________			□ < 24 hrs □ 1-3 d □ 4-7 d □ > 7 d □ Unknown

IF ILLNESS RESOLVED, skip to question #121

HOUSEHOLD SURVEY (7)

Household ID:

HH- |___|___|___|___|

Date of visit: |___|___| / |___|___| / |___|___|
day *month* *year*

THIRD ACTION

110. *"What did you do THIRD?" (choose one answer)*

□ Nothing
□ Tepid sponging
□ Gave herbs kept at home
□ Gave medicines kept at home
□ Other
(specify):______________________________
□ Bought medicines from shop
□ Took child to traditional healer
□ Took child to clinic or hospital
If clinic or hospital, go to question #111

111. *"If you took your child to clinic or hospital, where did you go?"*

□ Private clinic
□ Mulago specialty clinic
□ Private hospital
□ Mulago hospital
□ Other________________

MEDICINES GIVEN AS THIRD TREATMENT

"If your child took medicine THIRD, what did he/she take?

Drug	Number of times given per day	Number of days given	How long had the child been ill when the drug was started?
112. **Panadol**			□ < 24 hrs □ 1-3 d □ 4-7 d □ > 7 d □ Unknown
113. **Chloroquine**			□ < 24 hrs □ 1-3 d □ 4-7 d □ > 7 d □ Unknown
114. **Fansidar** *(SP)*			□ < 24 hrs □ 1-3 d □ 4-7 d □ > 7 d □ Unknown
115. **Amodiaquine**			□ < 24 hrs □ 1-3 d □ 4-7 d □ > 7 d □ Unknown
116. **Quinine**			□ < 24 hrs □ 1-3 d □ 4-7 d □ > 7 d □ Unknown
117. **Septrin** *(Bactrim)*			□ < 24 hrs □ 1-3 d □ 4-7 d □ > 7 d □ Unknown
118. **Amoxacillin**			□ < 24 hrs □ 1-3 d □ 4-7 d □ > 7 d □ Unknown
119. **Other:**______________			□ < 24 hrs □ 1-3 d □ 4-7 d □ > 7 d □ Unknown
120. **Other:**______________			□ < 24 hrs □ 1-3 d □ 4-7 d □ > 7 d □ Unknown

IF ILLNESS RESOLVED, go to question #121, otherwise continue sequence of events on HOUSEHOLD SURVEY form (10)

121. *"Did you think the child's illness was severe?"*
□ Yes
□ No
□ Unknown

122. *"Did the child have any of the following problems?"(tick all that apply)*
□ Convulsions
□ Loss of consciousness/coma
□ Difficulty breathing
□ Unable to sit or stand
□ Vomiting everything/severe vomiting
□ Child not able to drink or breast feed
□ None of the above

123. *"How long did the child's illness last?"*

□ < 24 hours
□ 1-3 days
□ 4-7 days
□ > 7 days
□ Unknown
□ Ongoing at initial screening
If ongoing, skip to question #125

124. *"Do you think the treatment helped cure the illness?"*
□ Yes
□ No
□ Unknown

125. *"Did the child have any problems with the treatments?"*
□ Yes
□ No *If no, skip to question #127*
□ Unknown

126. *"What problems occurred?" (tick all that apply)*

□ No appetite, refusing to breast feed
□ Vomiting
□ Stomach pain
□ Diarrhea
□ Itching
□ Skin rash
□ Convulsions, fits
□ Other__________________________

127. *"Other than you, who helped make decisions about treating the illness?" (define by relationship to primary caregiver AND tick all that apply)*
□ Wife □ Husband
□ Mother □ Father
□ Aunt □ Uncle
□ Sister □ Brother
□ No one
□ Other______________________

128. *"Did you experience any delays in treating your child's illness?"*

□ Yes
□ No *If no, skip to question #130*
□ Unknown

129. *"What were the reasons for the delays?" (tick all that apply)*

□ No transport available
□ Not enough money available
□ Needed to arrange for child care
□ Needed to find coverage for work
□ Other ________________________

130. *"How much did you spend on management of this illness?"*

Cost of drugs	|____|____|____|____|____|	**Ush**
Fees (clinic, hospital, lab)	|____|____|____|____|____|	**Ush**
Transport	|____|____|____|____|____|	**Ush**
Other	|____|____|____|____|____|	**Ush**
**TOTAL**	|____|____|____|____|____|	**Ush**

131. *"Did caring for your child and managing his/her illness prevent you from doing your usual activities this month?"*
□ Yes
□ No If no, skip to question #133

132. *"If yes, how many days did you miss?"* |____|____|

HOUSEHOLD SURVEY (8)	
**Household ID:**<br>**HH-** |___|___|___|___|	**Date of visit:** |___|___| / |___|___| / |___|___| *day* *month* *year*

"Finally, I would like to ask a few questions about each child that may participate in the study."

Study participant #1	Study participant #2	Study participant #3
**U-** |___|___|___|	**U-** |___|___|___|	**U-** |___|___|___|
133. *"What is your relationship to the child?"*<br>□ Mother □ Father<br>□ Grandmother □ Grandfather<br>□ Sister □ Brother<br>□ Aunt □ Uncle<br>□ Other ____________________	143. *"What is your relationship to the child?"*<br>□ Mother □ Father<br>□ Grandmother □ Grandfather<br>□ Sister □ Brother<br>□ Aunt □ Uncle<br>□ Other ____________________	151. *"What is your relationship to the child?"*<br>□ Mother □ Father<br>□ Grandmother □ Grandfather<br>□ Sister □ Brother<br>□ Aunt □ Uncle<br>□ Other ____________________
134. *"What is the birth order of the child?"*<br>□ First born<br>□ Last born<br>□ In between	144. *"What is the birth order of the child?"*<br>□ First born<br>□ Last born<br>□ In between	154. *"What is the birth order of the child?"*<br>□ First born<br>□ Last born<br>□ In between
135. *"Has the child lived in Kampala his/her entire life?*<br>□Yes *If yes, skip to question #138*<br>□ No	145. *"Has the child lived in Kampala his/her entire life?*<br>□Yes *If yes, skip to question #148*<br>□ No	155. *"Has the child lived in Kampala his/her entire life?*<br>□Yes *If yes, skip to question, #158*<br>□ No
136. *"How long has the child lived in Kampala?"*<br>□ < 6 months □ 5-10 years<br>□ 6-11 months □ > 10 years<br>□ 1- 4 years □ Unknown	146. *"How long has the child lived in Kampala?"*<br>□ < 6 months □ 5-10 years<br>□ 6-11 months □ > 10 years<br>□ 1- 4 years □ Unknown	156. *"How long has the child lived in Kampala?"*<br>□ < 6 months □ 5-10 years<br>□ 6-11 months □ > 10 years<br>□ 1- 4 years □ Unknown
137. *"Before living in Kampala, did the child live in a city, in a town, or in the countryside?"*<br>□ City □ Village<br>□ Town □ Unknown	147. *"Before living in Kampala, did the child live in a city, in a town, or in the countryside?"*<br>□ City □ Village<br>□ Town □ Unknown	157. *"Before living in Kampala, did the child live in a city, in a town, or in the countryside?"*<br>□ City □ Village<br>□ Town □ Unknown
138. *"Does the child sleep under a bed net?"*<br>□ Yes<br>□ No *If no, skip to end*<br>139. *"Did the child sleep under a bednet last night?"*<br>□ Yes □Unknown<br>□ No	148. *"Does the child sleep under a bed net?"*<br>□ Yes<br>□ No *If no, skip to end*<br>149. *"Did the child sleep under a bednet last night?"*<br>□ Yes □ Don't know<br>□ No	158. *"Does the child sleep under a bed net?"*<br>□ Yes<br>□ No *If no, skip to end*<br>159. *"Did the child sleep under a bednet last night?"*<br>□ Yes □ Don't know<br>□ No
140. *"Now let's talk about the net the child sleeps under most often. Has the net been treated with insecticide?"*<br>□ Yes - Perma net label<br>□ Yes - Smart net label<br>□ Yes - KO Net<br>□ Yes - by report<br>□ No *If no, skip to question #142*<br>□ Unknown<br>141. How long ago was the net last treated?<br>□ <6months □ >6months<br>□ Never □ Unknown	150. *"Now let's talk about the net the child sleeps under most often. Has the net been treated with insecticide?"*<br>□ Yes - Perma net label<br>□ Yes - Smart net label<br>□ Yes - KO Net<br>□ Yes - by report<br>□ No *If no, skip to question #152*<br>□ Unknown<br>151. How long ago was the net last treated?<br>□ <6months □ >6months<br>□ Never □ Unknown	160. *"Now let's talk about the net the child sleeps under most often. Has the net been treated with insecticide?"*<br>□ Yes - Perma net label<br>□ Yes - Smart net label<br>□ Yes - KO Net<br>□ Yes - by report<br>□ No *If no, skip to question #162*<br>□ Unknown<br>161. How long ago was the net last treated?<br>□ <6months □ >6months<br>□ Never □ Unknown
142. *ASK TO SEE NET AND DESCRIBE:*<br>□ Intact □ Visible holes<br>□ Not observed □ Not a bed net	152. *ASK TO SEE NET AND DESCRIBE:*<br>□ Intact □ Visible holes<br>□ Not observed □ Not a bed net	162. *ASK TO SEE NET AND DESCRIBE:*<br>□ Intact □ Visible holes<br>□ Not observed □ Not a bed net

Home visitor	Observers present at interview	Date form completed
|___|___|	|___|___| |___|___| |___|___|	|___|___| / |___|___| / |___|___| *day* *month* *year*

APPENDIX H. SAE and Serious Event REPORTING FORMS

SERIOUS ADVERSE EVENT FORM – INITIAL REPORT (1)

For randomized participants

Protocol title: Longitudinal Comparison of Combination Antimalarial Therapies in Ugandan Children: evaluation of safety, tolerability, and efficacy

**Principal Investigators:**<br>**Dr. Philip J. Rosenthal (UCSF)**<br>**Dr. Moses Kamya (MU)**	**Funding Source: NIH 1 U01 AI052142**<br>**DMID Protocol Number: 04-068**<br>**FDA IND Number: 69,577**	**UCSF-CHR Number: H2397-25789-01A**<br>**UNCST Number: MV 913**

**Date of SAE Report:**<br>|___|___|/|___|___|/|___|___| *day month year*	**Study Number: U - |___|___|___| -**	**Malaria Episode: |___|___|**
**Gender:**<br>☐ Male<br>☐ Female	**Age: ______years ______months**<br>*(include months only if age < 5)*	**Weight** *(kg)*: |___|___|

Event description:____
(symptom, sign, or laboratory abnormality)

**Indicate reason for serious AE** *(tick all that apply):*<br>☐ Death<br>☐ Life-threatening<br>☐ Resulted in significant / persistent disability or incapacity<br>☐ Resulted in or prolonged hospitalization<br>☐ Required medical / surgical intervention to prevent serious outcome<br>☐ Other:_______________	**Date of event onset:**<br>|___|___|/|___|___|/|___|___| *day month year*<br>**Maximum event severity:**<br>☐ Mild<br>☐ Moderate<br>☐ Severe<br>☐ Life-threatening	**Date event reported:**<br>|___|___|/|___|___|/|___|___| *day month year*<br>**DMID expectedness:**<br>☐ Yes ? Expected<br>☐ No ? Unexpected<br>**CHR expectedness:**<br>☐ Yes ? Expected<br>☐ No ? Unexpected

EVENT SUMMARY *(include details of event, associated signs and symptoms, possible alternative etiologies, relevant past medical history, and medical management):*

____
____
____
____
____
____
____
____
____
____
____
____

**Study product name: BLINDED**	**Route of study product: ORAL**
**Dosing schedule at SAE onset:**<br>**Date study product first started** *(Day 0 of malaria episode A)*:<br>|___|___|/|___|___|/|___|___| *day month year*	**Date study product last taken prior to onset of SAE:**<br>|___|___|/|___|___|/|___|___| *day month year*
**Relationship to study product:**<br>☐ Not associated<br>☐ Associated: *(possible, probable, definite)*	**If not associated, is event related to:**<br>☐ Study procedure<br>☐ Other condition or illness<br>☐ Other medication<br>☐ Other _______________

SERIOUS ADVERSE EVENT FORM – INITIAL REPORT (2)

For randomized participants

Date of SAE Report: |___|___|/|___|___|/|___|___| day month year	Study Number: U - |___|___|___|	Malaria Episode: |___|___|

Study product status: *(tick all that apply)*

☐ No change in dose
☐ Study treatment held
☐ Study treatment discontinued permanently
☐ Other ___________________________

Patient management: *(tick all that apply)*

☐ Patient hospitalized
☐ Blood transfusion given
☐ Intravenous fluids given
☐ Parenteral quinine given
☐ Other ___________________________
☐ Other ___________________________
☐ Other ___________________________

RELEVANT LABORATORY TESTS

Test	Collection date (dd/mm/yy)	Result	Site normal range	Most recent value prior to SAE	Collection date (dd/mm/yy)
	|___|___|/|___|___|/|___|___|				|___|___|/|___|___|/|___|___|
	|___|___|/|___|___|/|___|___|				|___|___|/|___|___|/|___|___|
	|___|___|/|___|___|/|___|___|				|___|___|/|___|___|/|___|___|
	|___|___|/|___|___|/|___|___|				|___|___|/|___|___|/|___|___|

RELEVANT DIAGNOSTIC TESTS

Test	Collection date (dd/mm/yy)	Results/Comments
	|___|___|/|___|___|/|___|___|	
	|___|___|/|___|___|/|___|___|	
	|___|___|/|___|___|/|___|___|	

CONCOMITANT MEDICATIONS *(List relevant concomitant medications the subject was taking up to 1 month prior to SAE onset.)*

Medication	Start date (dd/mm/yy)	Stop date (dd/mm/yy)	Total daily dose	Indication	Suspect for SAE
	|___|___|/|___|___|/|___|___|	|___|___|/|___|___|/|___|___|			? Yes ? No
	|___|___|/|___|___|/|___|___|	|___|___|/|___|___|/|___|___|			? Yes ? No
	|___|___|/|___|___|/|___|___|	|___|___|/|___|___|/|___|___|			? Yes ? No
	|___|___|/|___|___|/|___|___|	|___|___|/|___|___|/|___|___|			? Yes ? No
	|___|___|/|___|___|/|___|___|	|___|___|/|___|___|/|___|___|			? Yes ? No
	|___|___|/|___|___|/|___|___|	|___|___|/|___|___|/|___|___|			? Yes ? No

Outcome of event:

☐ Ongoing: [SAE Follow Up Report to be completed and sent at later date]
☐ Resolved without sequelae
☐ Resolved with sequelae ___________________________
☐ Death

If resolved or died, indicate date:

\|___\|___\|/\|___\|___\|/\|___\|___\|
day month year

Completed by:
Name printed: ___________________________ **Signature:** ___________________________ **Date:** \|___\|___\|/\|___\|___\|/\|___\|___\| day month year

Investigator's
Name printed: ___________________________ **Signature:** ___________________________ **Date:** \|___\|___\|/\|___\|___\|/\|___\|___\| day month year

*** Form will no longer be used past August 3, 2007***

SERIOUS EVENT FORM – INITIAL REPORT (1)<br>*For non-randomized participants only*		
Protocol title: Longitudinal Comparison of Combination Antimalarial Therapies in Ugandan Children: evaluation of safety, tolerability, and efficacy		
**Principal Investigators:**<br>**Dr. Philip J. Rosenthal (UCSF)**<br>**Dr. Moses Kamya (MU)**	**Funding Source: NIH 1 U01 AI052142**<br>**DMID Protocol Number: 04-068**<br>**FDA IND Number: 69,577**	**UCSF-CHR Number: H2397-25789-01A**<br>**UNCST Number: MV 913**

**Date of Event Report:**<br>|___|___|/|___|___|/|___|___| *day month year*	**Study Number: U -** |___|___|___|	
**Gender:**<br>☐ Male<br>☐ Female	**Age:** ______**years** ______**months**<br>*(include months only if age < 5)*	**Weight** *(kg)*: |___|___|

**Event description:**____<br>*(symptom, sign, or laboratory abnormality)*		
**Indicate reason for serious event** *(tick all that apply)*:<br>☐ Death<br>☐ Life-threatening<br>☐ Resulted in significant / persistent disability or incapacity<br>☐ Resulted in or prolonged hospitalization<br>☐ Required medical / surgical intervention to prevent serious outcome<br>☐ Other:__________________	**Date of event onset:**<br>|___|___|/|___|___|/|___|___| *day month year*<br>**Maximum event severity:**<br>☐ Mild<br>☐ Moderate<br>☐ Severe<br>☐ Life-threatening	**Date event reported:**<br>|___|___|/|___|___|/|___|___| *day month year*<br>**DMID expectedness:**<br>☐ Yes ? Expected<br>☐ No ? Unexpected<br>**CHR expectedness:**<br>☐ Yes ? Expected<br>☐ No ? Unexpected

EVENT SUMMARY *(include details of event, associated signs and symptoms, possible alternative etiologies, relevant past medical history, and medical management)*:

____

SERIOUS EVENT FORM – INITIAL REPORT (2)<br>*For non-randomized participants only*	
**Date of Event Report:** |___|___|/|___|___|/|___|___| *day month year*	**Study Number: U -** |___|___|___|

Patient management: *(tick all that apply)*

☐ Patient hospitalized
☐ Blood transfusion given
☐ Intravenous fluids given
☐ Other_______________________________
☐ Parenteral quinine given
☐ Other_______________________________
☐ Other_______________________________
☐ Other_______________________________

RELEVANT LABORATORY TESTS

Test	Collection date *(dd/mm/yy)*	Result	Site normal range	Most recent value prior to SAE	Collection date *(dd/mm/yy)*
	|__|__|/|__|__|/|__|__|				|__|__|/|__|__|/|__|__|
	|__|__|/|__|__|/|__|__|				|__|__|/|__|__|/|__|__|
	|__|__|/|__|__|/|__|__|				|__|__|/|__|__|/|__|__|
	|__|__|/|__|__|/|__|__|				|__|__|/|__|__|/|__|__|

RELEVANT DIAGNOSTIC TESTS

Test	Collection date *(dd/mm/yy)*	Results/Comments
	|__|__|/|__|__|/|__|__|	
	|__|__|/|__|__|/|__|__|	
	|__|__|/|__|__|/|__|__|	

CONCOMITANT MEDICATIONS *(List relevant concomitant medications the subject was taking up to 1 month prior to SAE onset.)*

Medication	Start date *(dd/mm/yy)*	Stop date *(dd/mm/yy)*	Total daily dose	Indication	Suspect for SAE
	|__|__|/|__|__|/|__|__|	|__|__|/|__|__|/|__|__|			? Yes<br>? No
	|__|__|/|__|__|/|__|__|	|__|__|/|__|__|/|__|__|			? Yes<br>? No
	|__|__|/|__|__|/|__|__|	|__|__|/|__|__|/|__|__|			? Yes<br>? No
	|__|__|/|__|__|/|__|__|	|__|__|/|__|__|/|__|__|			? Yes<br>? No
	|__|__|/|__|__|/|__|__|	|__|__|/|__|__|/|__|__|			? Yes<br>? No
	|__|__|/|__|__|/|__|__|	|__|__|/|__|__|/|__|__|			? Yes<br>? No
	|__|__|/|__|__|/|__|__|	|__|__|/|__|__|/|__|__|			? Yes<br>? No

**Outcome of event:**	**If resolved or died, indicate date:**
☐ Ongoing: [Follow Up Report to be completed and sent at later date]<br>☐ Resolved without sequelae<br>☐ Resolved with sequelae ___________________________<br>☐ Death	|___|___|/|___|___|/|___|___| *day month year*

Completed by:
Name printed: ___________________________ **Signature:** ___________________________ **Date:** \|___\|___\|/\|___\|___\|/\|___\|___\|
day month year

Investigator's
Name printed: ___________________________ **Signature:** ___________________________ **Date:** \|___\|___\|/\|___\|___\|/\|___\|___\|
day month year

SAE / SERIOUS EVENT FOLLOW-UP REPORT FORM

For randomized and non-randomized participants

***Protocol title:** Longitudinal Comparison of Combination Antimalarial Therapies in Ugandan Children: evaluation of safety, tolerability, and efficacy*

**Principal Investigators:**<br>**Dr. Philip J. Rosenthal (UCSF)**<br>**Dr. Moses Kamya (MU)**	**Funding Source: NIH 1 U01 AI052142**<br>**DMID Protocol Number: 04-068**<br>**FDA IND Number: 69,577**	**UCSF-CHR Number: H2397-25789-01B**<br>**UNCST Number: MV 913**

**Date of report:** |___|___|/|___|___|/|___|___| *day month year*	**Study Number: U - |___|___|___| -**	**Malaria Episode: |___|___|**
**Date event was first reported:** |___|___|/|___|___|/|___|___| *day month year*	**Age:** ______ **years** _____ **months**<br>*(include months only if age < 5)*	**Weight** *(kg):* |___|___|

EVENT:____

(symptom, sign, or laboratory abnormality)

Follow-up Report:

____

____

____

____

____

____

____

____

____

____

____

____

____

____

____

____

**Outcome of event:**<br>☐ Resolved without sequelae<br>☐ Resolved with sequelae ____________________<br>☐ Death	**If resolved or died, indicate date:** |___|___|/|___|___|/|___|___| *day month year*

Completed by:
Name printed: ____________________ **Signature:** ____________________ **Date:** |___|___|/|___|___|/|___|___|
day month year

Investigator's Name printed: ____________________ **Signature:** ____________________ **Date:** |___|___|/|___|___|/|___|___|
day month year

APPENDIX I. CASE REPORT FORMS

SCREENING FORM	Date of screening: |___|___|/|___|___|/|___|___| *day month year*	Household Number: HH- |___|___|___|___|	
**Last name**	**First name**		**Initials***
**Gender:** _____**M** _____**F**	**Age:** _______**years**______**months** *(include months only if age < 5)*		
**Weight:** __________**kg**	**Height:** __________**cm**	**WHZ-score**:	**HAZ-score:**

**Initial of last name, followed by initial of first name*

ASSESS DURING SCREENING INTERVIEW		
**Selection criteria**	**Include**	**Exclude**
1. Age 1 - 10 years	□ Yes	□ No
2. Agreement to come to study clinic for any febrile episode or other illness	□ Yes	□ No
3. Agreement to avoid medications administered outside the study	□ Yes	□ No
4. History of any known serious chronic illness requiring frequent medical care (*including AIDS, sickle cell disease, cancer)*<br>If yes, please indicate illness ____________________________________	□ No	□ Yes
5. Intention to move from Kampala during the study period	□ No	□ Yes
6. History of serious side effects to study medications or sulfa drugs	□ No	□ Yes
If any boxes in the "Exclude" column are ticked, exclude from the study. If not, proceed to the next section.		

INFORMED CONSENT DISCUSSION		
**Selection criteria**	**Include**	**Exclude**
7. Willingness of parents or guardians to provide informed consent	□ Yes	□ No
If the box in the "Exclude" column are ticked, exclude from the study. If not, proceed to the next section.		

MEASURE HEIGHT AND WEIGHT		
**Selection criteria**	**Include**	**Exclude**
8. Weight <10kg	□ No	□ Yes
9. Severe malnutrition defined as weight-for-height or height-for-age Z-score < – 3	□ No	□ Yes
If any boxes in the "Exclude" column are ticked, exclude from the study. If not, proceed to the next section.		

ASSIGN STUDY NUMBER	U|___|___|___|

BASELINE EVALUATION - COMPLETE ENROLLMENT FORM		
**Selection criteria**	**Include**	**Exclude**
10. Homozygous hemoglobin SS (sickle cell) by hemoglobin electrophoresis	□ No	□ Yes
11. Life-threatening screening laboratory value in the absence of malaria		
• Absolute neutrophil count < 250/mm^3	□ No	□ Yes
• Hemoglobin < 5.0 g/dL	□ No	□ Yes
• Platelet count < 25,000/mm^3	□ No	□ Yes
• Creatinine $\geq$ 1.5 mg/dL (< 2 years), $\geq$ 2.0 mg/dL ($\geq$ 2 years)	□ No	□ Yes
• ALT $\geq$ 15 x ULN ($\geq$ 450 U/L)	□ No	□ Yes
• Bilirubin $\geq$ 7.5 x ULN	□ No	□ Yes
12. Absence of symptomatic malaria, or response to antimalarial treatment	□ Yes	□ No
If any boxes in the "Exclude" column are ticked, exclude from the study. If not, proceed to the next section.		

All criteria for study inclusion met?	Date of enrollment and distribution of Insecticide Treated Net *(date study begins)*
□ Yes<br>□ No *If no, exclude from the study*	|___|___|/|___|___|/|___|___| *day month year*

***Form Version:* 14 March 2006**

PHASE II INITIATION FORM	**Date of visit:** |___|___|/|___|___|/|___|___| *day month year*	**Study Number** U|___|___|___|
	**Patient Initials:**	**Household Number:** HH- |___|___|___|___|

BED NET QUESTIONAIRE	
1. How many bed nets in total does the household have?	
2. Does child currently sleep under a bed net? (If no, STOP here)	☐ Yes<br>☐ No
3 If yes, how did the child sleep under a bed net last night?	☐ Yes<br>☐ No
4. Has the net been treated with insecticide?	☐ Yes - Perma net label<br>☐ Yes - Smart net label<br>☐ Yes - KO Net<br>☐ Yes - by report<br>☐ No<br>☐ Unknown
5. How long ago was the net last treated?	☐ <6months<br>☐ >6months<br>☐ Never<br>☐ Unknown
6. Was Home Visitor able to physically observe the bed nets? (If no, skip question 7)	☐ Yes<br>☐ No
7. In what condition is the child's bed net?	☐ Intact<br>☐ Visible holes<br>☐ Not observed<br>☐ Not a bed net

**8. Was Insecticide Bed Net Distributed and instructions for use given?**	**9. Date Phase II Begins:**
☐ Yes<br>☐ No	|___|___|/|___|___|/|___|___| *day month year*

ADDITIONAL CHILDREN FROM HOUSEHOLD	
10. How many children from household not currently enrolled and have not been previously enrolled in the study between ages 1 – 10 years?	
11. Please list ages of those children:	
12. Date guardian will bring to the study clinic: |___|___|/|___|___|/|___|___| *day month year*	

HOSPITAL ADMISSION FORM

**Patient initials:**	**Study Number: U|___|___|___|**	**Age:_______years______months** *(include months only if age < 5)*

Date of admission : |___|___|/|___|___|/|___|___| *day month year*	Study Day (if malaria):
Date of discharge* : |___|___|/|___|___|/|___|___| *day month year*	Study Day (if malaria):
Reason for admission:	
History:	Exam:
Laboratory Results:	
Assessment/Plan: *(List all medications given during hospitalization on FOLLOW UP FORMS)*	

**** Record date when patient has been discharged from the hospital***

***Form Version:* 18 October 2004**

HOSPITAL FOLLOW-UP FORM

**Patient initials:**	**Study Number:** U|___|___|___|	**Admit Date:** |___|___|/|___|___|/|___|___| ***day month year***

Date of follow-up: |___|___|/|___|___|/|___|___| *day month year*	Study Day (if malaria):	Temp:
Progress Note:<br><br>**Initials:________**		
Date of follow-up: |___|___|/|___|___|/|___|___| *day month year*	Study Day (if malaria):	Temp:
Progress Note:<br><br>**Initials:________**		
Date of follow-up: |___|___|/|___|___|/|___|___| *day month year*	Study Day (if malaria):	Temp:
Progress Note:<br><br>**Initials:________**		

***Form Version:* 18 October 2004**

HOUSEHOLD CONTACT FORM

CONFIDENTIAL INFORMATION: COMPLETE FORM AND FILE SEPARATELY

**Patient Initials:**	**Study Number:** U|___|___|___|	**Household Number:** **HH-** |___|___|___|___|
**Date of enrollment:**	|___|___|/|___|___|/|___|___| ***day month year***	**Age:_______years______months** *(include months only if age < 5)*

Participant's name *(last, first):*	
Head of household's name:	
Relationship to participant:	
Primary caregiver's name:	
Relationship to participant:	
Father's name:	
Mother's name:	
Name of other household member enrolled in this study:	Study Number: **U**|___|___|___|
Name of other household member enrolled in this study:	Study Number: **U**|___|___|___|
Name of other household member enrolled in this study:	Study Number: **U**|___|___|___|
Name of other household member enrolled in this study:	Study Number: **U**|___|___|___|
Name of other household member enrolled in this study:	Study Number: **U**|___|___|___|
Name of other household member enrolled in this study:	Study Number: **U**|___|___|___|
Home parish:	LC1:
Home address:	

Phone number: ___Yes ___No ___Unknown

If yes: Number: ____________________________ Name of phone owner: ________________________

Number: ____________________________ Name of phone owner: ________________________

***Form Version:* 18 October 2004**

**TERMINATION FORM**	**Study ID: U - |____|____|____|**	**Household Number: HH - |___|___|___|___|**
**First day of follow up:** |___|___|/|___|___|/|___|___| *day month year*	**Last day of follow up:** |___|___|/|___|___|/|___|___| *day month year*	**Patient Initials**

**Reason:**	
1. Movement out of Kampala for > 60 consecutive days	☐
2. Inability to be located for > 60 consecutive days	☐
3. Withdrawal of informed consent	☐
4. SAE probably or definitely related to study medications<br>5. Treatment failure to quinine plus clindamycin	☐<br>☐
6. Non-compliance to study schedule or procedures	☐
7. Diagnosis of a serious chronic disease requiring frequent medical care	☐
8. Death	☐

**Additional Comments:**

**Form completed by:**	**Date:**

**MALARIA FOLLOW-UP CLINICAL RECORD FORM (1)**	**Study Number:** U - |___|___|___|___|	**Day 0 Date:** |___|___|/|___|___|/|___|___| *day* *month* *year*	**Treatment:** ☐ Study medication (uncomplicated malaria) ☐ Quinine for Treatment Failure ☐ Quinine for Complicated Malaria (NOT for treatment failure) ☐ Quinine for Study Medication Hold ☐ Quinine/Clindamycin
	**Patient Initials:**	**Malaria Episode:** |___|___|	**Age:** |___|___| **years** |___|___| **months** *(include months only if age ≤ 2)*

SYMPTOM RECORD
(Rank on scale of 0-4: absent = 0; mild = 1; moderate = 2; severe = 3, life-threatening = 4, N/A = unable to assess)

	DAY 0 *	DAY 1	DAY 2	DAY 3	DAY ___	DAY ___	DAY ___	DAY ___	DAY ___
**DATE**									
**SYMPTOMS**									
Fever *(Y/N)* [grade]	[ ]	[ ]	[ ]	[ ]	[ ]	[ ]	[ ]	[ ]	[ ]
Weakness									
Headache†									
Anorexia									
Nausea†									
Vomiting									
Abdominal pain†									
Diarrhea									
Cough									
Pruritis									
"Flu"									
Other___________									
Other___________									
**AE reported** *(Y/N)*	XXXXXX								
**CLINICAL HISTORY RECORD**									
Record clinically relevant details of history									
Initials									

* *Transfer Day 0 information from INITIAL VISIT FORM*

† *Only assess in children > 3 years of age.*

**MALARIA FOLLOW-UP CLINICAL RECORD FORM (2)**	**Patient Initials:**	**Day 0 Date:** |___|___|/|___|___|/|___|___| *day month year*	**Study Number:** U- |___|___|___|	**Malaria Episode:** |___|___|

PHYSICAL EXAM RECORD

(Rank on scale of 0-4: absent = 0; mild = 1; moderate = 2; severe = 3, life-threatening = 4, N/A = unable to assess)

	DAY 0 *	DAY 1	DAY 2	DAY 3	DAY ___	DAY ___	DAY ___	DAY ___	DAY ___
**DATE**									
**PHYSICAL EXAM**									
Temperature (°C) [grade]	[ ]	[ ]	[ ]	[ ]	[ ]	[ ]	[ ]	[ ]	[ ]
Oropharynx									
Chest									
CVS									
Abdomen									
Skin									
Hearing									
Tablet test									
Other___________									
Other___________									
Other___________									
**Adverse event reported** *(Y/N)*	XXXXXX								
**ABNORMAL EXAM RECORD**									
If abnormality noted on physical exam, describe all physical findings for the abnormal exam									
Initials									

**Transfer Day 0 information from INITIAL VISIT FORM*

MALARIA FOLLOW-UP CLINICAL RECORD FORM (3)	Patient Initials:	Day 0 Date: |___|___|/|___|___|/|___|___| *day month year*	Study Number: U - |___|___|___|	Malaria Episode: |___|___|

MICROSCOPY RECORD									
	DAY 0	DAY 1	DAY 2	DAY 3	DAY __	DAY __	DAY __	DAY __	DAY __
DATE									
Parasite density *(/ul)*									
Species									
Gametocytes *(Y/N)*									
Initials									

LABORATORY RECORD								
DATE								
	DAY 0		DAY __		DAY __		DAY __	
	RESULT	Initials	RESULT	Initials	RESULT	Initials	RESULT	Initials
WBC *(/mm3)*								
% Neutrophils								
Neutrophils *(/mm3)* [grade]	[ ]		[ ]		[ ]		[ ]	
Hemoglobin *(g/dL)* [grade]	[ ]		[ ]		[ ]		[ ]	
Platelets *(/mm3)* [grade]	[ ]		[ ]		[ ]		[ ]	
ALT *(IU/L)* [grade]	[ ]		[ ]		[ ]		[ ]	
Other ____________ [grade]	[ ]		[ ]		[ ]		[ ]	
Other ____________ [grade]	[ ]		[ ]		[ ]		[ ]	
**Adverse event reported** *(Y/N)*								

TREATMENT OUTCOME	
☐ COMPLETE	☐ INCOMPLETE *Last STUDY DAY seen:*________
☐ETF ☐LCF ☐LPF ☐ACPR<br>If ETF or LCF, STUDY DAY of clinical failure: _______<br>If ETF or LCF, was it due to SEVERE MALARIA: ☐ No ☐ Yes *Criteria:* __________	☐ WITHDREW INFORMED CONSENT<br>☐ LOST<br>☐ OTHER ANTIMALARIAL: *Describe:*____________________<br>☐ CONCOMITANT FEBRILE ILLNESS: *Describe:*____________________

MALARIA FOLLOW-UP CLINICAL RECORD FORM (4) DAY 28 VISIT	Patient Initials:	Date of visit: |___|___|/|___|___|/|___|___| *day month year*	Study Number: U- |___|___|___|	Malaria Episode: |___|___|

TO BE COMPLETED BY MEDICAL OFFICER OR STUDY COORDINATOR

(1) Did this episode result in an early treatment or late clinical failure (ETF or LCF)? ☐ No ☐ Yes *(If yes, stop here)*

(2) Did a new malaria episode occur on days 15 – 30 of this episode? ☐ No ☐ Yes *(If yes, stop here)*

(3) Was day 28 visit missed? ☐ No ☐ Yes, *Reason:* ____________________ *(If yes, stop here)*

(4) On day 28 visit:

☐ No new complaints → *Obtain thick blood smear and filter paper sample. Record results of blood smear on this form below.*

☐ New complaint reported → *Complete an INITIAL VISIT form*

- ☐ No history of fever or temperature ≥ 38.0 °C found → *Record results of blood smear on this form below only*
- ☐ History of fever or temperature > 38.0 °C found
  - ☐ Negative blood smear result → *Record results of blood smear on this form below* and on INITIAL VISIT form
  - ☐ Positive blood smear result → *A) Record blood smear results on INITIAL VISIT form and begin new MALARIA FOLLOW-UP episode; B) On DAY 28 form, complete question (2) above and do not complete rest of form*

TO BE COMPLETED BY HOME VISITOR OR MEDICAL OFFICER

Day 28 visit done: ☐ **Home** ☐ **Study Clinic**

Did the patient receive any medical care outside of the study since last seen? ☐ Yes ☐ No *(If no, skip below)*

Where treatment received	Date	Diagnosis	BS done?

Did the patient receive any medications outside of the study since last seen? ☐ Yes ☐ No *(If no, skip below)*

Medications	Dose/Duration	Date last given

History of fever in last 24 hours?	Temperature *(°C)*	*Initials*
☐ Yes * ☐ No	|___|___|•|___| *	

* *If history of fever or temperature ≥ 38.0 °C, BRING PARTICIPANT TO THE STUDY CLINIC*

BLOOD SMEAR RESULTS	
Parasite density *(/ul)*	
Gametocytes (Y/N)	
Initials	

**INITIAL VISIT FORM (1)**	**Patient Initials:**	**Date of visit:** |___|___|/|___|___|/|___|___| *day month year*	**Study Number:** U- |___|___|___|___|	**Age:** |___|___| **years** |___|___| **months** *(include months only if age ≤ 2)*
**Was patient brought to clinic for routine blood smear ≥ 100,000 parasites/mL?** ☐ Yes ☐ No			**Did the patient receive any medications outside of the study since the last visit?** ☐ Yes ☐ No *(If no, skip section on Outside Medications)*	

OUTSIDE MEDICATIONS *(List any medications taken outside of the study since last seen)*

Drug	Where treatment received?	Dose *(# of tablets/injections)*	Complete dose? *(Y/N or N/A)*	Last date given

VITAL SIGNS	SYMPTOMS	Severity†	Duration	Comment	PHYSICAL EXAM	Severity†	Comment
**Respiratory Rate**	**Fever** *(Y/N)* [grade]	[ ]			**Temperature** *(°C)* [grade]	[ ]	
|___|___|	Weakness				Pallor		
	Headache*				Eyes		
**Heart rate**	Anorexia				Ears		
	Nausea*				Oropharynx		
|___|___|___|	Vomiting				Neck		
	Abdominal pain*				Chest		
**Height** *(cm)*	Diarrhea				CVS		
	Cough				Abdomen		
|___|___|___|	Pruritis				Skin		
	"Flu"				Hearing		
**Weight** *(kg)*	Other ________				Tablet test		
|___|___|	Other ________				Other ________		
	**AE reported** *(Y/N)*				**AE reported** *(Y/N)*		
Initials	*Initials*				*Initials*		

*Only assess if > 3 yrs of age † Rank on scale of 0-4: absent = 0; mild =1; moderate =2; severe =3, life-threatening =4, N/A =unable to assess)

LAB TESTS				ADDITIONAL INVESTIGATIONS				DISPOSITION
Test	Ordered?	Results	Initials	Other Test	Ordered?	Results	Initials	**FOLLOW-UP**
**Parasite density** *(/ul)*	☐ Yes ☐ No				☐ Yes			☐ Malaria follow-up*
Gametocytes *(Y/N)*					☐ Yes			☐ Non-malaria follow-up
Urgent Hb								☐ Follow-up not indicated
WBC *(mm3)*					☐ Yes			**REFERRAL**
% Neutrophils	☐ Yes ☐ No							☐ To Acute Care Unit
Neutrophils *(mm3)*		[ ]			☐ Yes			☐ Other________
Hemoglobin *(g/dL)*		[ ]						☐ Other________
Platelets *(/mm3)*		[ ]			☐ Yes			
ALT *(IU/L)*	☐ Yes ☐ No	[ ]						*Initials*

* If MALARIA FOLLOW-UP is indicated, write "SEE MALARIA FOLLOW-UP" and record lab test result on MALARIA FOLLOW-UP FORM (3); otherwise list on this form.

INITIAL VISIT FORM (2)<br>DIAGNOSES / MEDICATIONS	**Patient Initials:**	**Date of initial visit:** |___|___|/|___|___|/|___|___| ***day month year***	**Study Number:** **U-** |___|___|___|

**Diagnoses**	**Code**	**Date Diagnosed**	***Initials***

**Medication**	**Code**	**Dose and Duration**	**Indication**	**Date Prescribed**	**Initials**

**NON-MALARIA FOLLOW-UP CLINICAL RECORD FORM (1)**	**Patient Initials:**	**Day 0 Date:** |___|___|/|___|___|/|___|___| *day month year*	**Study Number:** U-|___|___|___|
		**Age:** |___|___| **years** |___|___| **months** *(include months only if age ≤ 8)*	

SYMPTOM RECORD

(Rank on scale of 0-4: absent = 0; mild = 1; moderate = 2; severe = 3, life-threatening = 4, N/A = unable to assess)

	DAY 0 *	DAY ___	DAY ___	DAY ___	DAY ___	DAY ___	DAY ___	DAY ___	DAY ___
**DATE**									
**SYMPTOMS**									
Fever *(Y/N)* [grade]	[ ]	[ ]	[ ]	[ ]	[ ]	[ ]	[ ]	[ ]	[ ]
Weakness									
Headache†									
Anorexia									
Nausea†									
Vomiting									
Abdominal pain†									
Diarrhea									
Cough									
Pruritis									
"Flu"									
Other____________									
Other____________									
**Adverse event reported** *(Y/N)*									
**CLINICAL HISTORY RECORD**									
Record clinically relevant details of history									
Initials									

** Transfer Day 0 information from INITIAL VISIT FORM*

† Only assess in children ≥ 3 years of age.

**NON-MALARIA FOLLOW-UP CLINICAL RECORD FORM (2)**	**Patient Initials:**	**Date of initial visit:** |___|___|/|___|___|/|___|___| *day month year*	**Study Number:** **U-** |___|___|___|

**PHYSICAL EXAM RECORD** *(Rank on scale of 0-4: absent = 0; mild = 1; moderate = 2; severe = 3, life-threatening = 4, N/A = unable to assess)*									
	**DAY 0 ***	**DAY ___**	**DAY ___**	**DAY ___**	**DAY ___**	**DAY ___**	**DAY ___**	**DAY ___**	**DAY ___**
**DATE**									
**PHYSICAL EXAM**									
Temperature *(ºC)* [grade]	[ ]	[ ]	[ ]	[ ]	[ ]	[ ]	[ ]	[ ]	[ ]
Eyes									
Ears									
Oropharynx									
Chest									
CVS									
Abdomen									
Skin									
Hearing									
Tablet test									
Other____________									
Other____________									
Other____________									
**Adverse event reported** *(Y/N)*									
**ABNORMAL EXAM RECORD**									
If abnormality noted on physical exam,, describe all physical findings for the abnormal exam									
Initials									

**Transfer Day 0 information from INITIAL VISIT FORM*

NON-MALARIA FOLLOW-UP CLINICAL RECORD FORM (3)	Patient Initials:	Date of initial visit:|___|___|/|___|___|/|___|___| *day* *month* *year*	Study Number: U- |___|___|___|

LABORATORY TESTING									
**DATE**									
	**DAY 0**			**DAY____**			**DAY ____**		
**Test**	**Ordered?**	**Results**	**Initials**	**Ordered?**	**Results**	**Initials**	**Ordered?**	**Results**	**Initials**
**Parasite density *(/ul)***	□ Yes □ No			□ Yes □ No			□ Yes □ No		
Gametocytes *(Y/N)*									
Urgent Hb									
WBC *(/mm3)*	□ Yes □ No			□ Yes □ No			□ Yes □ No		
% Neutrophils									
Neutrophils *(/mm3)*		[ ]			[ ]			[ ]	
Hemoglobin *(g/dL)*		[ ]			[ ]			[ ]	
Platelets *(/mm3)*		[ ]			[ ]			[ ]	
ALT *(IU/L)*	□ Yes □ No	[ ]		□ Yes □ No	[ ]		□ Yes □ No	[ ]	
**AE reported** *(Y/N)*									

ADDITIONAL INVESTIGATIONS				NOTES	DISPOSITION
**Other Test**	**Ordered?**	**Results**	**Initials**		**FOLLOW-UP**<br>□ Begin Malaria Follow-up<br>|___|___|/|___|___|/|___|___| ***day*** ***month*** ***year***
	□ Yes				
	□ Yes				
	□ Yes				**REFERRAL**<br>□ To Acute Care Unit<br>|___|___|/|___|___|/|___|___| ***day*** ***month*** ***year***<br>□ Other:__________________<br>□ Other:__________________
	□ Yes				
	□ Yes				
	□ Yes				
	□ Yes				
	□ Yes				***Initials***

**ENROLLMENT FORM (1)**	**Patient Initials:**	**Study Number:** U- |___|___|___|	**Household Number:** HH- |___|___|___|___|
		**Today's Date:** |___|___|/|___|___|/|___|___| *day month year*	**Age:** |___|___| **years** |___|___| **months** *(include months only if age ≤ 8)*

PAST MEDICAL HISTORY	KNOWN DRUG ALLERGIES:		
**Prior illnesses** *(include dates, if available)*<br>___<br>___<br>___<br>___<br>___	**KNOWN DRUG ALLERGIES:** □ Yes □ No □ Unknown<br>*If yes, describe*:___		
	**VACCINATION HISTORY***		
	**Vaccine** (full course)	**Received (Y/N/U)†**	**Dates received / Notes****
**Prior surgeries** *(include dates, if available)*<br>___<br>___<br>___	BCG	□ Y □ N □ U	
	Polio (OPV)	□ Y □ N □ U	
	Tetanus (DPT)	□ Y □ N □ U	
**Medications taken chronically** *(include indication)*<br>___<br>___<br>___	Measles	□ Y □ N □ U	
	Other___	□ Y □ N □ U	
	Other___	□ Y □ N □ U	

** Review immunization card if available.* *†Y = yes, N = no, U = unknown* ***If full course given, write "FULL COURSE", otherwise record dates of vaccine doses received*

VITAL SIGNS	SYMPTOMS	Severity†	Duration	Comment	PHYSICAL EXAM	Severity†	Comment
**Weight** *(kg)*	**Fever** *(Y/N)* [grade]	[ ]			**Temperature** *(ºC)* [grade]	[ ]	
|___|___|	Weakness				Pallor		
	Headache*				Eyes		
**Height** *(cm)*	Anorexia				Ears		
	Nausea*				Oropharynx		
|___|___|___|	Vomiting				Neck		
	Abdominal pain*				Chest		
**HAZ-score (circle + or -):**	Diarrhea				CVS		
	Cough				Abdomen		
+ / - |___| • |___|___|	Pruritis				Skin		
	"Flu"				Hearing		
**WHZ-score (circle + or -):**	Other ___				Tablet test		
	Other ___				Other ___		
+ / - |___| • |___|___|	Other ___				Other ___		
	Other ___				Other ___		
Initials	***Initials***				***Initials***		

** Only assess in children ≥ 3 years of age.* *† Rank on scale of 0-4: absent = 0; mild = 1; moderate = 2; severe = 3, life-threatening = 4, N/A = unable to assess)*

**ENROLLMENT FORM (2)**	**Patient Initials:**	**Today's Date:** |___|___|/|___|___|/|___|___| *day month year*	**Study Number:** U- |___|___|___|

**DIAGNOSES***		**ADDITIONAL MEDICATION RECORD†**				
Diagnosis	Code	Medication	Code	Indication	Date Prescribed	Initials

** List all diagnoses made during screening/enrollment visits.*

† List all medications prescribed during screening/enrollment visits.

**LABORATORY TESTS**		
	**Result**** [grade]	**Initials**
Parasite density *(/ul)* *†		
Gametocytes (Y/N) *†		
WBC *(/mm3)*		
% Neutrophils		
Neutrophils *(/mm3)*	[ ]	
Hemoglobin *(g/dL)*	[ ]	
Platelets *(/mm3)*	[ ]	
ALT *(IU/L)*	[ ]	
Bilirubin *(mg/dL)*	[ ]	
Creatinine *(mg/dL)*	[ ]	
Hb electrophoresis		
G-6-PD		

Thick blood smear notes:

** If history of fever in previous 24 hours or temperature ≥ 38.0ºC, do URGENT blood smear.*

† If urgent blood smear is POSITIVE, treat with quinine and repeat blood smear in 7 days. Complete MALARIA AT ENROLLMENT FORM and attach to the ENROLLMENT FORM.

Laboratory test notes:

*** If there is no history of fever and temperature < 38.0ºC, evaluate results of screening labs within 72 hours. If labs meet exclusion criteria (life-threatening), exclude.*

Date to return for lab results: |___|___|/|___|___|/|___|___|
day month year

DISPOSITION

□ **Malaria at Enrollment follow up†**

□ **Enrolled**

Date of enrollment: |___|___|/|___|___|/|___|___|
day month year

□ **Excluded**

Date of exclusion: |___|___|/|___|___|/|___|___|
day month year

MALARIA AT ENROLLMENT FORM (1)	Patient Initials:	Day 0 Date: |___|___|/|___|___|/|___|___| day month year	Study Number: U- |___|___|___|

For malaria at enrollment treated with QUININE, follow-up on Days 0-7. If patient requires treatment with QUININE + CLINDAMYCIN, follow-up on Days 8-14.

SYMPTOM RECORD (Rank on scale of 0-4: absent = 0; mild = 1; moderate = 2; severe = 3, life-threatening = 4, N/A = unable to assess)									
	**DAY 0***	**DAY ____**	**DAY ____**	**DAY ____**	**DAY ___**	**DAY ____**	**DAY ____**	**DAY ____**	**DAY ___**
**DATE**									
**SYMPTOMS**									
Fever *(Y/N)* [grade]	[ ]	[ ]	[ ]	[ ]	[ ]	[ ]	[ ]	[ ]	[ ]
Weakness									
Headache†									
Anorexia									
Nausea†									
Vomiting									
Abdominal pain†									
Diarrhea									
Cough									
Pruritis									
"Flu"									
Other____________									
Other____________									
**CLINICAL HISTORY RECORD**									
Record clinically relevant details of history									
Initials									

**Transfer Day 0 information from ENROLLMENT FORM* *† Only assess in children ≥ 3 years of age*

MALARIA AT ENROLLMENT FORM (2)	Patient Initials:	Day 0 Date: |___|___|/|___|___|/|___|___| *day month year*	Study Number: U- |___|___|___|

For malaria at enrollment treated with QUININE, follow-up on Days 0-7. If patient requires treatment with QUININE + CLINDAMYCIN, follow-up on Days 8-14.umu

PHYSICAL EXAM RECORD<br>***(Rank on scale of 0-4: absent = 0; mild = 1; moderate = 2; severe = 3, life-threatening = 4, N/A = unable to assess)***									
	**DAY 0***	**DAY ___**	**DAY ___**	**DAY ___**	**DAY ___**	**DAY ___**	**DAY ___**	**DAY ___**	**DAY ___**
**DATE**									
**PHYSICAL EXAM**									
Temperature *(ºC)* [grade]	[ ]	[ ]	[ ]	[ ]	[ ]	[ ]	[ ]	[ ]	[ ]
Oropharynx									
Chest									
CVS									
Abdomen									
Skin									
Hearing									
Tablet test									
Other____________									
Other____________									
Other____________									
**ABNORMAL EXAM RECORD**									
If abnormality noted on physical exam,, describe all physical findings for the abnormal exam									
Initials									

**Transfer Day 0 information from ENROLLMENT FORM*

MALARIA AT ENROLLMENT FORM (3)	Patient Initials:	Day 0 Date: |___|___|/|___|___|/|___|___| day month year	Study Number: U- |___|___|___|

THICK BLOOD SMEAR RECORD	URGENT BLOOD SMEAR*		REPEAT BLOOD SMEAR (DAY 7)		REPEAT BLOOD SMEAR (DAY 14)	
	DATE		DATE		DATE	
	Parasite density†		Parasite density‡		Parasite density**	
	Initials		*Initials*		*Initials*	
BLOOD SMEAR INSTRUCTIONS	* Transfer day of screening information from ENROLLMENT FORM.<br><br>† If POSITIVE → *Treat with quinine and repeat blood smear in 7 days.*		‡ If NEGATIVE → *Check baseline screening labs.*<br><br>‡ If POSITIVE → *Treat with quinine and clindamycin and repeat blood smear in 7 days (on Day 14).*		** If NEGATIVE → *Check baseline screening labs.*<br><br>** If POSITIVE → *Exclude.*<br><br>*Date of exclusion:* |___|___|/|___|___|/|___|___| day month year	

ANTIMALARIAL TREATMENT RECORD	TREATMENT (1)		REPEAT TREATMENT (2)		
	Medication	**Quinine**	Medication	**Quinine**	**Clindamycin**
	Date prescribed		Date prescribed		
	Dose		Dose		
	Initials		*Initials*		

LABORATORY TEST RECORD	BASELINE SCREENING LAB TESTS*‡			REPEAT LAB TESTS ††		
	DATE			DATE		
	Test	**Result** [grade]	Initials	Test	**Result** [grade]	Initials
	WBC *(/mm3)*			WBC *(/mm3)*		
	% Neutrophils			% Neutrophils		
	Neutrophils *(/mm3)*	[ ]		Neutrophils *(/mm3)*	[ ]	
	Hemoglobin *(g/dL)*	[ ]		Hemoglobin *(g/dL)*	[ ]	
	Platelets *(/mm3)*	[ ]		Platelets *(/mm3)*	[ ]	
	ALT *(IU/L)*	[ ]		ALT *(IU/L)*	[ ]	
	Bilirubin *(mg/dL)*	[ ]		Bilirubin *(mg/dL)*	[ ]	
	Creatinine *(mg/dL)*	[ ]		Creatinine *(mg/dL)*	[ ]	
	Other ________________	[ ]		Other ________________	[ ]	
	Other ________________	[ ]		Other ________________	[ ]	
LABORATORY INSTRUCTIONS	* Transfer day of screening information from ENROLLMENT FORM.<br><br>‡ Are LIFE-TREATENING lab results present?<br><br>□ NO → Complete Screening Form<br><br>□ YES → *Repeat labs and have patient return in 48-72 hours for lab results.*			†† Are LIFE-TREATENING lab results present?<br><br>□ NO → Complete Screening Form<br><br>□ YES → Exclude<br>*Date of exclusion:* |___|___|/|___|___|/|___|___| day month year		

ROUTINE VISIT FORM	Patient Initials:	Study Number: U- |___|___|___|	Date of visit: |___|___|/|___|___|/|___|___| *day* *month* *year*

TO BE COMPLETED BY HOME VISITOR OR MEDICAL OFFICER						
**Did the patient receive any medical care outside of the study since last seen?** ☐ Yes ☐ No (If no, skip below)				**Did the patient receive any medications outside of the study since last seen?** ☐ Yes ☐ No (If no, skip below)		
**Where treatment received**	**Date**	**Diagnosis**	**BS done?**	**Medications**	**Dose/Duration**	**Date last given**

**History of fever in last 24 hours?** ☐ Yes * ☐ No	**Temperature** *(°C)* |___|___|•|___| *	Last night the child slept under:	☐ ITN bed net<br>☐ Untreated bed net<br>☐ Bed net, unknown if treated<br>☐ Did not use a net	*Initials*
** If history of fever or temperature ≥ 38.0 °C, EVALUATE IN CLINIC*				

TO BE COMPLETED ONLY BY MEDICAL OFFICER IN CLINIC

ARE THERE ANY NEW COMPLAINTS AT THIS VISIT? ☐ Yes ☐ No **IF YES, COMPLETE SYMPTOMS & PHYSICAL EXAM BELOW**

**VITAL SIGNS**	**SYMPTOMS**	**SEVERITY†**	**DURATION**	**COMMENT**	**PHYSICAL EXAM**	**SEVERITY†**	**COMMENT**
**Height** *(cm)* |___|___|___|	**Fever** *(Y/N)* [grade]	[ ]			**Temperature** *(°C)* [grade]	[ ]	
	Weakness				Pallor		
	Headache*				Eyes		
**Weight** *(kg)* |___|___|	Anorexia<br>Nausea*				Ears<br>Oropharynx		
	Vomiting				Neck		
	Abdominal pain*				Chest		
Initials	Diarrhea				CVS		
	Cough				Abdomen		
	Pruritis				Skin		
	"Flu"				Hearing		
	Other __________				Tablet test		
	Other __________				Other __________		
	**AE reported** *(Y/N)*				**AE reported** *(Y/N)*		
	Initials				*Initials*		

LAB TESTS				ADDITIONAL INVESTIGATIONS		
**Test**	**Ordered?**	**Results**	**Initials**	**Other Test**	**Results**	**Initials**
Parasite density *(/ul)*	☐ Yes ☐ No					
Gametocytes *(Y/N)*						
WBC *(/mm3)*	☐ Yes ☐ No					
% Neutrophils						
Neutrophils *(/mm3)*		[ ]				
Hemoglobin *(g/dL)*		[ ]				
Platelets *(/mm3)*		[ ]				
ALT *(IU/L)*	☐ Yes ☐ No	[ ]				
	AE reported *(Y/N)*					

** Only assess in children > 3 years of age* *† Rank on scale of 0-4: absent = 0; mild = 1; moderate = 2; severe = 3, life-threatening = 4, N/A = unable to assess)*

VERSION 14 AUGUST 2006

**ADVERSE EVENT RECORD FORM**	**Patient Initials:**	**Study Number:** U|___|___|___|	**Adverse Event Record Form Page #** _______

To be completed by MEDICAL OFFICER											***To be completed by STUDY COORDINATOR***			
**Event description**	**Date event onset**	**Date event reported**	**Maximum severity***	**Relationship** *(0-3)†*	**Serious** *(Y/N)*	DMID Expected ‡ ***(Y/N)***	CHR Expected** ***(Y/N)***	Explanation ***(Y/N)*** ††	**Expedited reporting? *(Y/N)* ‡‡**	**Initials of person reporting**	Reviewed **Relationship†**	**Outcome** Δ	**Date outcome assigned**	**Initials**

* **Severity:** *Rank on scale of 1-4: mild = 1; moderate = 2; severe = 3, life-threatening = 4*

† **Relationship:** *Rank on scale of 0-3: none = 0; possible = 1; probable = 2; definite = 3*

‡ **DMID Expected:** *Is this event an elevation of ALT? Yes or No*

** **CHR Expected:** *Could this event be expected to occur in association with malaria, or is it reported on ANY package insert (Appendix E)? Yes or No*

†† **Explanation:** *Is there an alternative explanation for this event? Yes or No*

‡‡ **Expedited reporting:** *Events that are serious or CHR unexpected with no alternative explanation (CHR Expected = No and Explanation = No) should be reported promptly. Refer to protocol or SOP for guidelines on reporting to DMID and IRBs.*

Δ **Outcome:** *Rank on scale of 1-5: resolved without sequelae = 1; resolved with sequelae = 2; AE still present at study end/discontinuation, but improving = 3; subject died = 4; unknown = 5*

APPENDIX J SEIZURE 6-MONTLY SAFETY LOG

Date Prepared: _______________ Prepared by: ______________

MU-UCSF UO1 MULAGO III COHORT STUDY: SEIZURE 6-MONTHLY SAFETY LOG									
Subject ID	Age	Treatment Group	Onset Date	Index case	Last date study meds taken prior to event	Outcome	Date of resolution / Duration	Was convulsion associated with fever?	Clinical History

* Key to Abbreviations: PMH = past medical history, GTC = generalized tonic-clonic, BS = blood smear, CBC = complete blood count, WNL = within normal limits, I+D = incision and drainage, ABX = antibiotics, D/c'ed = discharged.

DMID Protocol # 04-068
